# HMGB1 bound to cisplatin–DNA adducts undergoes extensive acetylation and phosphorylation *in vivo*†
†Electronic supplementary information (ESI) available. See DOI: 10.1039/c4sc03650f


**DOI:** 10.1039/c4sc03650f

**Published:** 2014-12-15

**Authors:** Yafeng He, Yin Ding, Dan Wang, Wanjun Zhang, Weizhong Chen, Xichun Liu, Weijie Qin, Xiaohong Qian, Hao Chen, Zijian Guo

**Affiliations:** a State Key Laboratory of Coordination Chemistry , State Key Laboratory of Analytical Chemistry for Life Science , School of Chemistry and Chemical Engineering , Nanjing University , No. 22 Hankou Road , Nanjing , 210093 P. R. China . Email: chenhao@nju.edu.cn ; Email: zguo@nju.edu.cn; b National Center for Protein Sciences Beijing , State Key Laboratory of Proteomics , Beijing Proteome Research Center , Institute of Radiation Medicine , 33 Life Science Park Road, Changping District , Beijing , 102206 P. R. China

## Abstract

Cisplatin, one of the most effective anticancer drugs, is a DNA-damaging agent that induces cell death primarily by apoptosis. For many years, HMGB1 has been known to be a recognition protein for cisplatin–DNA lesions. Here, an application of a biomolecular probe based on a peptide–oligonucleotide conjugate is presented as a novel method for investigating this recognition process *in vivo*. Proteins known to be involved in the recognition of cisplatin-damaged DNA were pulled down and identified, including members of the HMGB family and a number of other proteins. Interestingly, at least 4 subforms of HMGB1 bind to cisplatin–DNA adducts. These proteins were further identified as post-translationally acetylated or phosphorylated forms of HMGB1. These results provide a rich pool of protein candidates whose roles in the mechanism of action of platinum drugs should be explored. These newly discovered molecular components of the DNA damage signalling cascade could serve as novel links between the initial cell responses to DNA damage and the downstream apoptotic or DNA repair pathways.

## Abstract 2

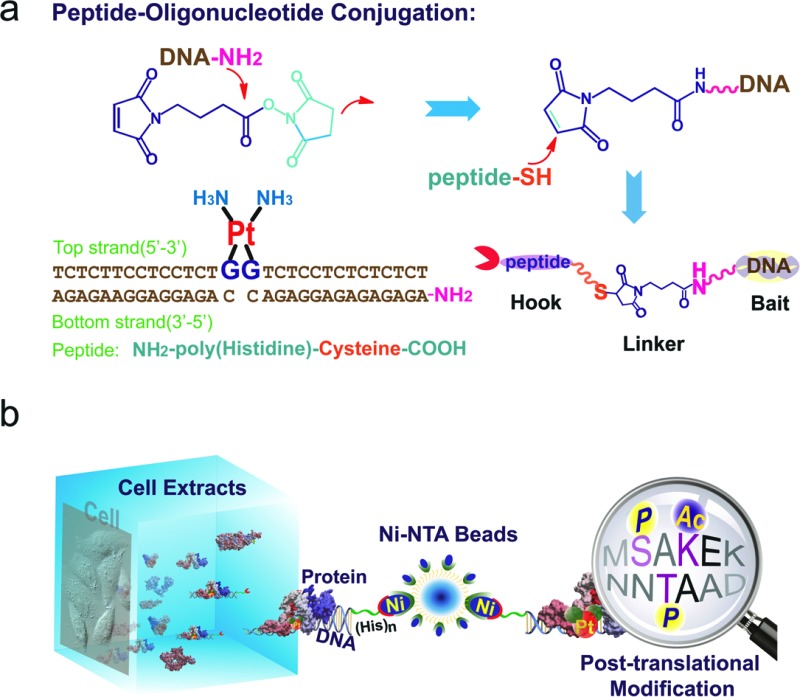
Here, an application of a biomolecular probe based on a peptide–oligonucleotide conjugate is presented as a novel method for investigating the recognition of cisplatin–DNA lesions by HMGB1 *in vivo*.

## Introduction

Cisplatin, one of the most widely used anticancer drugs, binds DNA and primarily forms 1,2-d(G*pG*) and 1,2-d(A*pG*) intra-strand cross-links; less frequently, 1,3-d(G*pTpG*) cross-links and inter-strand cross-links are formed.^1^ Similar to most clinical anticancer drugs targeting DNA, it is believed that the cisplatin–DNA adducts initiate a series of cellular events, such as blocking DNA replication and gene transcription, triggering diverse signalling pathways. Together, these effects eventually lead to apoptosis or systematic cell death.^2^–^5^ To counteract these effects, DNA repair proteins in the nucleus form a self-defence system against this DNA damage. The removal of certain DNA lesions *via* DNA repair pathways provides an opportunity for the cancer cell to survive.^6^ Regardless of the type of DNA damage, the recognition of these cisplatin–DNA adducts by certain proteins is the first step in the induction of most downstream cellular events. The direct interaction of Pt–DNA adducts with sensor proteins stimulates the nucleus to generate diverse types of functional machinery that perform standard cell crisis responses. However, the molecular basis for this signalling cascade is still under investigation. Various methods have been used to identify many proteins as Pt–DNA adduct-binding factors.^7^,^8^ These proteins include DNA damage repair proteins, such as nucleotide excision repair proteins (NER),^9^ mismatch repair proteins (MMR),^10^ DNA-dependent protein kinases (DNA-PK), HMG-domain proteins, and several other cellular factors, such as TBP, p53, hUBF, and PARP-1.^11^–^15^ Among the most studied of these factors, HMG domain proteins were found to bind preferentially to cisplatin-modified DNA.^16^

The HMGB proteins, which belong to the large family of HMG domain proteins, contain three primary members: HMGB1, HMGB2 and HMGB3. HMGB1, which has been considered to be a primary recognition factor of cisplatin–DNA adducts, shows a remarkably high affinity to cisplatin–DNA cross-links.^17^ As a multifunctional non-chromatin nuclear protein, HMGB1 acts as a molecular chaperon between distorted DNA and various proteins.^18^ In contrast to the stabilisation of chromatin by histones, HMGB1 binds with linker DNA between the chromatin cores and destabilises the compact chromatin DNA. This effect provides access for DNA repair proteins or transcription factors to their cognate DNA sites.^19^ Through this interaction with HMGB1, proteins that are involved in DNA repair or proapoptotic pathways can respond to cisplatin-induced DNA damage.^20^,^21^ The phosphorylation of p53 at Ser9 and Ser15 22 and the phosphorylation of γ-H2AX have been considered DNA damage hallmarks of chemotherapy treatment.^23^ These biological modifications of key proteins are reduced in HMGB1-deficient cells. This fact suggests that HMGB1 is an essential activator of early cellular responses to genotoxicity. Moreover, there is solid evidence that HMGB1 interacts directly with p53 24 (enhanced by phosphorylation of p53 25), and phosphorylated p53 was also shown to co-localise with γ-H2AX.^26^ These proteins are well known for their critical roles in both apoptosis induction and DNA repair protein recruitment. However, the precise role of the HMG recognition of Pt–DNA adducts is still obscure despite being extensively studied. As an active area of current research, the modulatory mechanisms of DNA damage detection and signal transduction are extremely critical for our understanding of cisplatin pharmacology. Our results could also provide major insight into cellular self-defence systems and cisplatin resistance.

## Results

### Probe construction and pull-down

**Fig. 1:**
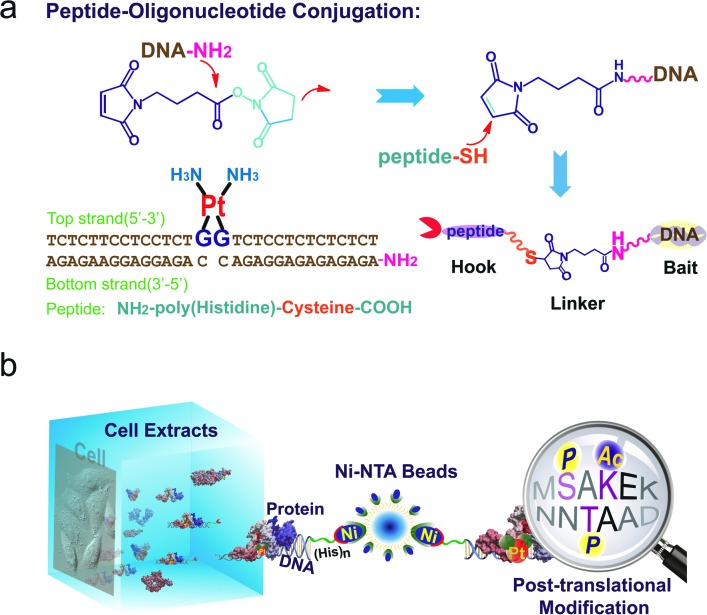
(a) Construction of the peptide–DNA conjugate. Oligonucleotides (bottom strand) with a primary amino group on the 5′ end (DNA–NH_2_) are conjugated to the peptide *via* a 2-step reaction with a bifunctional linker. The conjugate is annealed with the top strand DNA harbouring the cisplatin 1,2-GG cross-link. (b) A pull-down experiment is conducted with cell extracts using agarose beads conjugated to the DNA probes. The proteins that are captured are digested into peptide fragments and identified using mass spectrometry.

To study the recognition of cisplatin-damaged DNA by cellular proteins, we constructed a peptide–oligonucleotide conjugate (POC)-based^27^ biomolecular probe to capture Pt–DNA–protein ternary complexes and to isolate these complexes intact from cell extracts. For the design of this probe, a poly-His peptide, which acts as an immobilisation hook, and an oligonucleotide, which contains a site-specific cisplatin cross-link and acts as protein binding bait, are covalently linked with a hetero-bifunctional cross-linker (Fig. 1a).^28^,^29^ This probe was fully characterised using MALDI-TOF MS, chromatography and a thermal stability assay (Fig. S1–S3†). With non-cisplatin-modified POC as a control probe, we established a well-defined control panel that could discriminate between proteins contributing to cisplatin–DNA adduct recognition rather than general DNA-binding proteins (*e.g.*, histone, zinc finger proteins). The addition of a poly-histidine tail to the DNA resulted in a conjugate that binds to Ni^2+^–NTA beads (*K*_d_ = 10^–13^ M) at a pH of approximately 8.0.^30^ This coordination bond could be precisely tuned with a low concentration of imidazole independent of the ionic strength. A suitable amount of imidazole as a competitor was able to reduce the non-specific binding remarkably, in turn increasing the sensitivity of the method (Fig. S4†). SKOV3 is a cisplatin-insensitive ovarian cancer cell line that rapidly becomes resistant upon continuous treatment with the compound. Thus, this cell line provides numerous protein candidates with potential roles in cisplatin toxicity and drug resistance. As shown in Fig. 1b, a pull-down experiment is conducted with cell extracts. Cell disruption should be performed under mild conditions to maintain the integrity of the proteins and protein complexes. We primarily focused on results obtained from SKOV3 cells. In addition, several other cell lines were studied using the same procedure to prove the generalisability of this method. These data are not shown here because of their similarity to the data from the SKOV3 cells.

### HMGB1 protein subforms bind to cisplatin–DNA cross-links

**Fig. 2:**
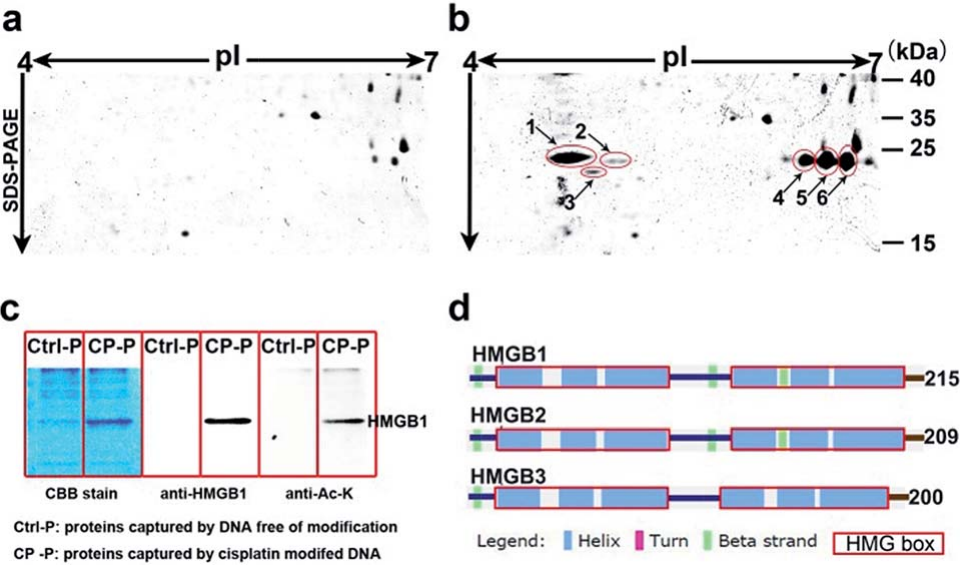
Display of proteins captured using cisplatin–DNA adducts. Compared with the control panel (a), 6 spots were marked and identified using mass spectrometry (b); the same proteins pulled down using probes were resolved with SDS-PAGE, stained with Coomassie bright blue and immunoblotted with anti-HMGB1 and anti-acetylated lysine antibodies (c); schematic illustration of the secondary structures of the HMGB proteins (d).

Based on well-reproducible 2-dimensional electrophoresis (2-DE) gel analysis, protein spots corresponding to cisplatin–DNA adducts can be clearly distinguished from other non-specific proteins after matching with the control 2-DE map. Combined with MALDI-TOF MS and immunoblotting, a number of protein candidates are identified (Fig. 2a and b, Table 1, Fig. S5,† and Table S1†). Given the high-quality MS spectra obtained from these protein spots, peptide mass fingerprinting (PMF) searches can be conducted using the Mascot online server to identify the different proteins. It is unsurprising that HMGB1/2, the most prominent cisplatin–DNA binding proteins, were identified by this novel probe. Another member of the HMGB family, HMGB3, was also identified and validated using western blotting (Fig. S6†).

**Table 1:** Proteins identified with affinity to cisplatin-containing probe after isolation from cell extracts

No.	Protein ID	MW	pI^*a*^	Score^*b*^
1	High-mobility group protein B1	25 049	5.45	60
2	High-mobility group protein B2	24 190	7.94	95
3	High-mobility group protein B3	22 980	8.48	43^*c*^
4	High-mobility group protein B1	25 049	5.45	62
5	High-mobility group protein B1	25 049	5.45	109
6	High-mobility group protein B1	25 049	5.45	94

^*a*^Theoretical pI from ProMoST.

^*b*^Mascot online server PMF score.

^*c*^HMGB3 is identified through its unique peptide fragment MS/MS ions.

Surprisingly, most of the HMGB proteins that bind to the cisplatin–DNA adduct were post-translationally modified (PTM) forms, including HMGB1, HMGB2 and HMGB3. Using the isoelectric focusing technique, at least four HMGB1 spots (Fig. 2b) and two HMGB2 spots (Fig. S7†) with essentially the same molecular weights but different pIs (isoelectrical points) were identified using peptide mass fingerprinting. The apparent pI of HMGB3 is approximately 4.8, which is very different from its canonical pI of 8.48 (Fig. 2b). These results were verified through 2-DE followed by western blotting (2D-WB). The theoretical pIs predicted using the protein modification screening tool (ProMoST)^31^ for canonical HMGB1/2 are 5.45 and 7.94, respectively. Both of these proteins that were captured and identified with the described probe exhibit multiple types of modification, and the protein spot on the acidic end of the 2-DE strip shows a remarkable pI shift. These results suggest the presence of different forms of HMGB protein that vary in their global charge distribution. As HMGB1, HMGB2 and HMGB3 exhibit over 80% amino acid sequence identity and possess the same DNA binding domains,^19^ (Fig. 2d), further detailed studies focused on the forms of HMGB1 with diverse pI isoforms and their important biological functions. According to a previous study, HMGB1 might be acetylated *in vivo*. Therefore, the HMGB1 protein pulled down with our probe was immunoblotted with a specific anti-acetylated lysine antibody (Fig. 2c). The result clearly shows that at least a portion of the HMGB1 that binds to the Pt–DNA adduct is acetylated in the cell.

**Fig. 3:**
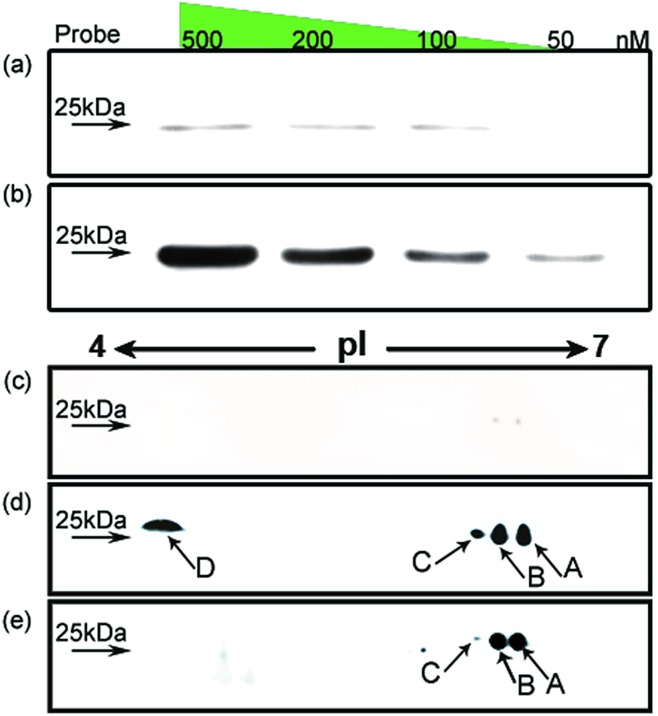
Investigation of HMGB1 isoforms in the cell. HMGB1 is captured from SKOV3 cell extracts using the probe with (b) or without (a) cisplatin cross-link; the concentration of the probe is varied from 50 to 500 nM. The analysis using 2DE–WB shows the HMGB1 isoforms trapped with the cisplatin probe (d) or immunoprecipitated with an anti-HMGB1 antibody (e); (c) probe without cisplatin cross-link acts as a control.

Using HMGB1 as an internal marker, the pull-down results indicate that this protein could bind to cisplatin-damaged DNA at a DNA concentration of 50 nM (Fig. 3b). This binding is stronger than the *in vitro* measured binding affinity (*K*_d_ = 120 nM) of HMGB1 for the cisplatin–DNA cross-link.^32^ To survey the natural distribution of the PTM isoforms of HMGB1 *in vivo*, an anti-HMGB1 antibody was used to immunoprecipitate (IP) HMGB1 in the same cell extract used in the capturing experiments with the probe. The IP proteins were resolved with 2-DE and then immunoblotted with the same antibody. As shown in Fig. 3e, there is a unique distribution of HMGB1 PTM isoforms in the cell, and isoforms A and B can be recognised using two different methods with similar binding affinities. However, isoforms C and D exhibit higher binding affinities to the probe containing the cisplatin lesion than to the traditional antibody. The protein spots pattern of HMGB1 in Fig. 3d is consistent with the pattern in Fig. 2b. The HMGB1 subform D exhibits a highly significant pI difference compared to the subforms A–C, suggesting its unique modification status. In addition, the four subforms captured with our probe are found in markedly different abundances. These variable abundances might suggest their different binding affinities to the cisplatin–DNA cross-link.

### Mapping the acetylation and phosphorylation sites of the HMGB1 subforms

**Fig. 4:**
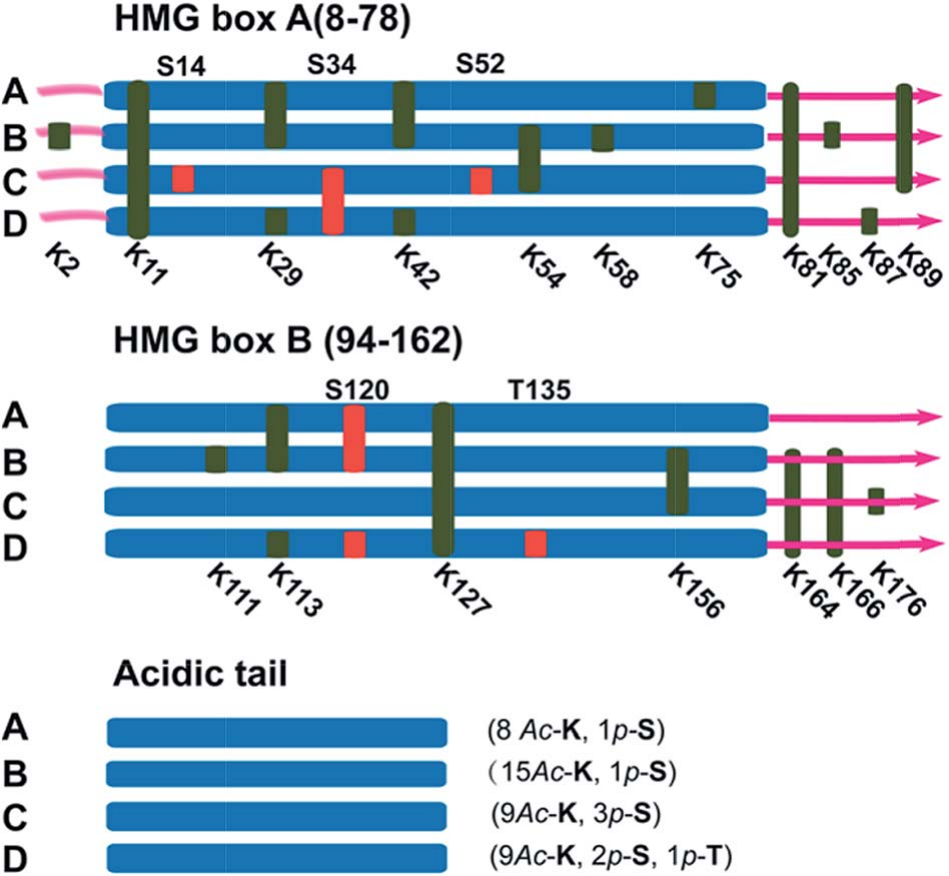
Illustration of the post-translational modification sites of the HMGB1 isoforms. Acetylated lysine sites are marked with K (green ribbon), and phosphorylated serine or threonine sites are abbreviated as S or T (red ribbon).

It has been reported in protein translocation studies that several HMGB1 subforms in the calf thymus or activated human monocytes correspond to different acetylation statuses.^33^ Two isoforms of HMGB1 that possess a small number of acetylation sites were reported more than three decades ago.^34^ Here, we show that there are at least four post-translationally modified forms of HMGB1 that recognise the cisplatin–DNA 1,2-(GpG) cross-link. To determine the precise modification sites, all isoforms were separated on a 2-DE gel and fully characterised using high-resolution LC-MS/MS. As shown in Fig. 4, a total of 23 modification sites were identified (Table S2, Fig. S8†). Surprisingly, both of the HMG boxes of HMGB1, which are known DNA binding domains, are hyper-acetylated. In addition, four acetylated lysines are located in the basic linker sequence between the two HMG boxes, and three acetylated lysines are present in the linker sequence between HMG box B and the acidic tail. However, no modifications were detected in the acidic tail, indicating its unique conserved property in this protein. In total, 16 acetylated lysine sites were detected in both the A and B isoforms of HMGB1. For the C and D isoforms, three phosphorylation sites were observed for each. For isoform C, three phosphorylated serines are crowded within HMG box A. Isoform D has one phosphorylated serine in each HMG box and contains a phosphorylated threonine in HMG box B. These two phosphorylation patterns of HMGB1 have never been detected before. Considering the low abundance of phosphorylated peptides in the mass spectrum data, we believe there could be more phosphorylated sites than were detected in isoform D, resulting in its significant pI change.

## Discussion

Post-translational modifications of proteins are among the most important cellular events involved in signal transduction. As the most common reversible modifications of proteins *in vivo*, acetylation and phosphorylation at distinct amino acids alter both the local molecular structure and the charge distribution of the parental protein; further changing the entire protein conformation to regulate interactions with different binding partners.^35^ The study of HMGB1 acetylation has primarily focused on lys2. *In vitro* experiments have demonstrated that acetylated lys2 enhances the HMGB1 binding affinity to distorted DNA substrates (*e.g.*, UV- and cisplatin-damaged DNA, 4-way junctions).^36^ Nevertheless, it is difficult to explain how acetylated lysines affect the HMGB1 binding affinity to cisplatin–DNA lesions without further insight into this protein. The newly identified acetylation sites (Table S2†) could provide a wide variety of patterns that regulate HMGB1 interactions with DNA or companion proteins.^36^,^37^ For example, as one of the most important proteins interacting with HMGB1, p53 is believed to control several key biological processes along with HMGB1. Our preliminary Co-IP assay results (Fig. S9†) indicate that the p53 protein indeed interacts directly with acetylated HMGB1, and p53 phosphorylated at ser20 could be recognized but not that phosphorylated at ser15. This distinction suggests that the acetylation pattern of HMGB1 discussed above might play a critical role in the p53 pathway which is related to the DNA damage response.^38^ Even more importantly, two phosphorylated HMGB1 subforms have also been identified. The phosphorylation of certain amino acids alters the local charge much more than acetylation, an effect that directly results in the large pI shift of the protein. It is interesting that all phosphorylation sites are located in the two HMG boxes, which are assumed to have different functions based on recent evidence. Box A may interact with other transcription factors as a protein chaperone, and box B may act as a DNA-bending factor.^18^,^25^ The interplay of the HMG boxes and the acidic tail is believed to modulate the binding affinity between HMGB1 and its substrates.^39^,^40^ The HMG box modification sites revealed here may participate in the interaction with the acidic tail. Specific acetylation and phosphorylation events neutralise the basic HMG boxes and their short basic linker. This change in the local charge distribution disrupts the mask effect of the acidic tail, reinforcing DNA binding and bending abilities. The possible biological relevance of this discovery may be rooted in the local conformational change that occurs after phosphorylation. Increased binding and bending abilities of phosphorylated HMGB1 could facilitate the exposure of damaged DNA to repair proteins or other signal factors. It could be hypothesised that HMGB1 both (i) enhances the interaction between cisplatin–DNA lesions and other repair proteins and (ii) recognises DNA damage sites and recruits repair proteins through protein–protein interactions. In both situations, the binding strength can be subtly modulated with post-translational modifications, as discussed above. Nevertheless, the cellular events that occur after the recognition of damage sites are unclear; studies of these downstream molecular mechanisms are required.

## Conclusions

In summary, we have developed a systematic method for the discovery of proteins that are correlated with cisplatin pharmacology. Interestingly, we found that certain HMGB1 subforms within the cell are post-translationally modified. First, unexpected hyper-acetylation was detected on the four HMGB1 isoforms. Furthermore, the PTM isoforms C and D, which are differentially phosphorylated, exhibit fairly high-affinity binding to the cisplatin–DNA adduct. These results provide an in-depth view of the cisplatin–DNA–protein interaction *in vivo*. Furthermore, these findings may provide new clues towards the improvement of existing chemotherapies in terms of efficiency and overcoming resistance.

## Supplementary Material

Supplementary information
