# Supplementary material for: HMGB1 bound to cisplatin–DNA adducts undergoes extensive acetylation and phosphorylation in vivo
Source: Chem Sci. 2014 Dec 15;6(3):2074–8. doi: 10.1039/c4sc03650f (PMC5810237; doi:10.1039/c4sc03650f)
Supplement: Supplementary file 1 [file SC-006-C4SC03650F-s001.pdf]

Electronic Supplementary Material (ESI) for Chemical Science.
This journal is © The Royal Society of Chemistry 2015

HMGB1 bound to Cisplatin-DNA Adduct Undergoes Extensive Acetylation and Phosphorylation in Vivo

Yafeng He[a], Yin Ding[a], Dan Wang[a], Wanjun Zhang[b], Weizhong Chen[a], Xichun Liu[a], Weijie Qin[b], Xiaohong Qian[b], Hao Chen[a*] and Zijian Guo[a*]

[a] State Key Laboratory of Coordination Chemistry, Coordination Chemistry Institute, State Key Laboratory of Analytical Chemistry for Life Science, School of Chemistry and Chemical Engineering, Nanjing University, 22 Hankou Road, Nanjing, 210093 (P. R. China);

[b] National Center for Protein Sciences Beijing, State Key Laboratory of Proteomics, Beijing Proteome Research Center, Institute of Radiation Medicine, 33 Life Science Park Road, Changping District, Beijing, 102206 (P. R. China)

*Corresponding Author E-mail: chenhao@nju.edu.cn; zguo@nju.edu.cn

Supporting information

Table of Content

1. Experiment procedures S3

1.1 Chemical sources and instruments S3

1.2 Cell culture S3

1.3 Cell lysis S3

1.4 Site-specific DNA platination and purification S3

1.5 Oligonucleotide-peptide conjugation S4

1.6 Annealing double strand DNA probe S4

1.7 Thermal stability of dsDNA probe S4

1.8 Affinity isolation of proteins S5

1.9 Sample preparation for 2-dimensional electrophoresis S5

1.10 2-Dimensional electrophoresis S5

1.11 Peptide mass fingerprinting and nanoLC-MS/MS S6

1.12 Western blotting S6

1.13 Co-immunoprecipitation assay S6

2. Results S7

2.1 Probe characterization S7

2.2 Imidazole as a competitor to reduce non-specific binding to Ni-NTA beads. S8

2.3 Two dimensional electrophoresis gel image and protein identification S9

2.4 Westen blot validation S10

2.5 Post-translational modification (PTM) identification S10

2.6 Raw LC-MS/MS data for PTM profiling of HMGB1 subform A S17

2.7 Raw LC-MS/MS data for PTM profiling of HMGB1 subform B S23

2.8 Raw LC-MS/MS data for PTM profiling of HMGB1 subform C S29

2.9 Raw LC-MS/MS data for PTM profiling of HMGB1 subform D S33

2.10 Acetylated HMGB1 interacts with p53 and its phosphorylated forms S39

1. Experiment procedures

1.1 Chemical sources and instruments

The 5'- or 3'-amino-modified oligonucleotides were purchased from Invitrogen; cisplatin and potassium tetrachloroplatinate were provided by Shandong Boyuan Pharmaceutical Corporation, Shandong province in China; GMBS was purchased from SpeedChemical Corporation. The peptide was synthesised using standard Fmoc-based chemistry by the Genscript Corporation, Nanjing City, China. The Ni-NTA agarose beads and Histrap HP affinity column were purchased from GE Healthcare. The 2-DE experiments were performed on the Ettan IPGphor 3 system, made by GE Healthcare. The antibodies uesd for western blotting were purchased from Abcam. Real-time PCR detection was conducted in a CFX96 Touch™ system using reagents provided by BioRad. A reverse-phase C18 column was purchased from GLscience. All of the other solvents were HPLC-grade and were used as received.

1.2 Cell culture

We purchased the human ovarian carcinoma cell line SKOV3 from American Type Culture Collection. The cells were maintained at 5% CO_2 at 37 °C in RPMI-1640 with 10% FBS. The cells were treated with variable concentrations of platinum complexes.

1.3 Cell lysis

The cells were harvested by first digesting with trypsin, followed by washing twice with PBS at 4 °C after low-speed centrifugation, the cell pellet was flashfrozen in liquid nitrogen. The cell lysis buffer formulation was as follows: 20 mM Tris, pH 7.4, 1 mM Na_3VO_4, 250 mM NaCl, 0.5% NP-40, 2 mM NaF, 1 mM PMSF Protease Inhibitor Cocktail (Sigma), 5 mM DTT. We used a Dounce homogeniser, with 15 strokes every 10 min on ice to homogenise the cells. Cell homogenisation was performed by centrifugation at 12000 rpm for 10 min at 4 ºC. Supernatants were transferred to clean tubes and used as the cell extract for the next step.

1.4 Site-specific DNA platination and purification

To prepare the diaqua cisplatin derivative, we mixed 1.97 equiv. of $AgNO_3$ with cis-[$Pt(NH_3)_2Cl_2$] in water at 37 °C in the dark. After 5 h, the reaction mixture was filtered to remove the AgCl. The filtrate was incubated at 37 °C for an additional 2 h filtered again, and stored at -20 °C in the dark. The activated cisplatin solution was mixed with deoxyoligonucleotides (ODN1) in 10 mM HEPES (pH 6.0) at 37 °C in the

dark for 8 h. The platinated oligonucleotides were purified using an InertSustain C18 column (GLscience). The purification buffer included A) 20 mM NH_4Ac, pH=7.0 as the equilibrium condition and B) 80% methanol in buffer A to generate the elution condition. The oligonucleotide mixture was lyophilised and dissolved in 100μl buffer A for sample injection. The sample loaded on the column was washed with 12.5% buffer B. We then generated a gradient of 12.5-48% buffer B to wash away unplatinated oligonucleotides. A hold of several column volumes of 50% buffer B was used to remove most of the multiply-platinated oligonucleotides. We then eluted monoadducts of cisplatin with 100% buffer B. All of the Pt-DNA adducts were fully characterised by chromatography and MALDI-TOF.

1.5 Oligonucleotide-peptide conjugation

Oligonucleotides (ODNs) with a 5'-or 3' aminolinker were synthesised by Invitrogen and supplied in lyophilised powder. We dissolved this lyophilised powder in ultrapure water and determined its concentration. Approximately 280 μM ODNs was allowed to react with 20 equiv. GMBS dissolved in acetonitrile (usually 50 mM) in PBS (pH=7.4) at 30 °C for 40 min. The mixture was desalted using Sephadex G-25 (GE Healthcare) to remove excess GMBS. The fraction was lyophilised and dissolved in ultrapure water. The final conjugation step was carried out with 80 μM modified ODNs in PBS (pH=7.0). Next, 0.5 equiv. peptide was mixed with ODNs every 15 min at 30 °C; this step was repeated 6-7 times. After buffer exchange(20mM NH_4Ac) through G-25, the excess peptide was removed. We used a syringe to load samples on a Histrap HP affinity column (GE Healthcare). The column was washed with 90 mM imidazole and then eluted with 500 mM imidazole. The fraction (POC) was purified using a G25 column to remove imidazole, and the fraction was then lyophilised.

1.6 Annealing double strand DNA probe

Platinated ODNs were hybridised with 1 equiv. POC in an annealing buffer including 20 mM Tris (pH 8.0), 100 mM NaCl, 50 mM KCl and 5 mM $MgCl_2$. Double-strand ODN-annealing was conducted by first heating the mixture to 95 °C and then cooling slowly to 4°C (approximately 2 h). In the required control experiments, double-strand ODNs without platinum compound modifications were prepared in the same way.

1.7 Thermal stability of dsDNA probe

The probes were divided into 10 mL aliquots and mixed with 1:1000 diluted SYBR green I dye solution, ensuring that the final composite of this solution was 20 mM Tris (pH 7.4), 150 mM NaCl, 5 mM $MgCl_2$. The Experiments were performed on CFX96 Touch™ Real-Time PCR Detection System, with the temperature increasing from 30 °C to 95 °C at 0.5 °C step per minute. The fluorescent signal was recorded

with the increasing temperature. The derivatives of the curve gave the dsDNA melting temperature.

1.8 Affinity isolation of proteins

Approximately 50 μl Ni-NTA Sepharose beads were incubated with 100 μg dsODNs (with or without incorporated cisplatin) at 4 °C for 30 min in binding buffer (20 mM Tris (pH 7.4), 150 mM NaCl). This incubation allowed the dsODNs to conjugate to the sepharose beads. Freshly prepared whole cell extracts (4-10 mg protein, 80 mM imidazole, pH 7.4) were added, and this mixture was rotated gently at 4 °C for 4 h. The supernatant was removed by centrifuging at 5000 rpm for 1 min. The beads were washed at least 5 times with PBS containing 80 mM imidazole. SDS (200 μl, 2%) was added to the beads, followed by heating to 95 °C. As much supernatant was collected as possible.

1.9 Sample preparation for 2-dimensional electrophoresis

Proteins isolated from cell extract in 2% SDS were precipitated to remove surfactant contaminants with a 2-DE protein clean up kit (GE Healthcare). The protein pellet was air-dried and dissolved in 2-DE rehydration buffer (6 M urea, 2 M thiourea, 2% CHAPS and approximately 0.1% bromophenol blue). Appropriate DTT and IPG buffer (GE Healthcare) concentrations were added to the solution immediately before dry strip rehydration.

1.10 2-Dimensional electrophoresis

DTT (18 mM for IPG3-10, 50 mM for IPG4-7) and IPG buffer (IPG4-7 or 3-10) were added to the sample solution at appropriate concentrations. The IPG dry strip (7 cm) for the first dimension isoelectric focusing (IEF) was passively rehydrated in sample solution for at least 10 h. The rehydrated strip was then isoelectric focused using the following protocol: 2 h at 100 V, 1 h at 500 V, 1 h gradient increase to 1000 V, 1 h gradient to 4000 V, and a hold at 4000 V for a total of 22000 Vhr. Then, the IPG strips were reduced using a 1% DTT solution and alkylated using 2.5% iodoacetamide for 15 min. The SDS-PAGE was performed on a 12% SDS resolving gel and stained with Coomassie Brilliant Blue R250. The gels were scanned with a gel scanner (GE Healthcare)

1.11 Peptide mass fingerprinting and nanoLC-MS/MS

The gel spots were removed from the gel using a MiniTip. The proteins were digested with trypsin in gel. The peptides were extracted from the gel pieces first by 0.1%

formic acid, followed by extraction buffer (60% acetonitrile, 35% H_2O, 5% formic acid); sonication was used twice in this process. All of the solutions that were generated in the peptide extraction were combined and concentrated for protein identification. Peptide fingerprint mapping was performed on an ABSCIEX 4800 MALDI-TOF/TOF instrument. The peptide mass lists were searched against the UniProt human protein database with Mascot distiller.

LC-MSn experiments were performed on a QE mass spectrometer (Thermo Fisher) equipped with a nanospray source and Eksigent high-performance liquid chromatography. The peptide mixture was separated on a fused silica microcapillary column with an internal diameter of 75 μm and an in-house prepared needle tip with an internal diameter of approximately 15 μm. Peptide separation was achieved using a mobile phase of 1.95% ACN, 97.95% H_2O, 0.1% FA (phase A) and 79.95% ACN, 19.95% H_2O, 0.1% FA (phase B). The linear gradient was from 5% to 50% buffer B for 80 min at a flow rate of 300 nL/min. The database search parameters included up to 2 missed cleavages for full tryptic digestion, a precursor ion mass tolerance of 6 ppm, a product ion mass tolerance of 0.02 m/z units, cysteine carbamidomethylation as a fixed modification, and methionine oxidation as variable modifications. For all of the peptide and protein identifications, the estimated false discovery rate (FDR) was fixed at a maximum of 1%.

1.12 Western blotting

Proteins that were resolved by 2-DE and SDS-PAGE were transferred to PVDF (Millipore, 0.22 μm) in Towbin buffer containing 0.033% SDS. PVDF membranes were blocked for 1 h at ambient temperature in blocking buffer (5% skim milk/0.1% Tween-20/PBS). The primary antibodies in appropriate dilutions were incubated with the membranes at 4°C overnight. The blots were washed with PBST (0.1% Tween-20/PBS) and incubated with peroxidase-conjugated secondary antibody in washing buffer for 1 h. After washing with PBST, the blots were visualised by enhanced chemiluminescence kit from Millipore.

1.13 Co-immunoprecipitation assay

Total protein was extracted from SKOV-3 cell pellet using homogenizer with NP40 lysis buffer. Approximately 3.6 mg of total protein was precleared with 20 μl of 50% protein A/G slurry at 4 °C for 15 min. Precleared lysate was then immunoreacted with p53 polyclonal antibody, anti-p53 (phospho S15) antibody and anti-p53 (phospho S20) antibody, respectively, at 4 °C for 8 h. The protein-antibody complexes were then immunoprecipitated by adding 50 μl of 50% protein A/G sepharose slurry at 4 °C for 4 h. The beads were washed 3 times with PBS and then eluted with SDS-PAGE loading buffer at 90 °C for 3 min. Samples were subjected to western detection and

blotted with anti-lysine antibody and anti-HMGB1 successively.

2. Results

2.1 Probe characterization

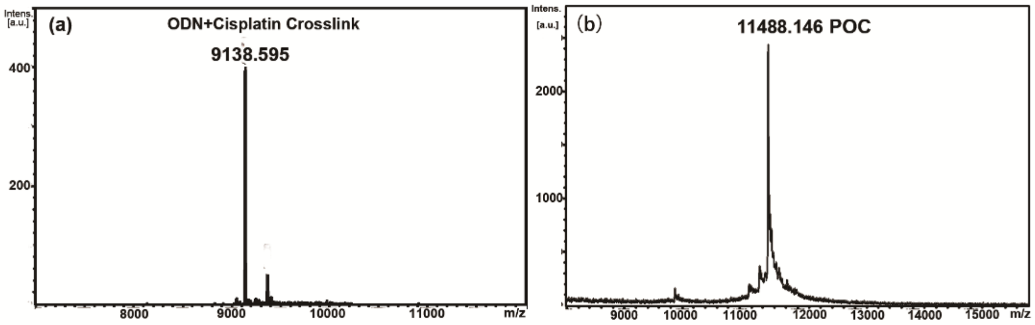

Figure S1. MALDI-TOF mass spectrum of Peptide-DNA conjugation probe.(a) DNA is platinated site specifically by cisplatin on GpG, (b)complementary strand was conjugated with polyhistidine.

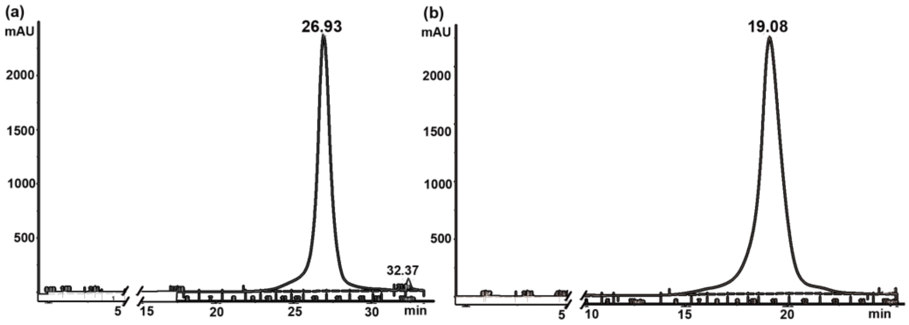

Figure S2. Probes with(b) or without (a) cisplatin modification were purified and characterized by monoQ column under native condition after annealing.

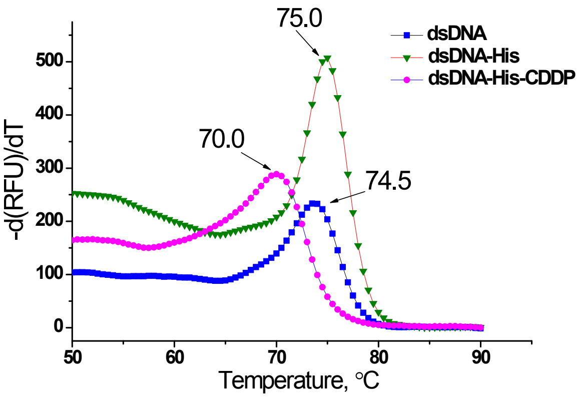

Figure S3. Thermal stability of dsDNA probes.

2.2 Imidazole as a competitor to reduce non-specific binding to Ni-NTA beads

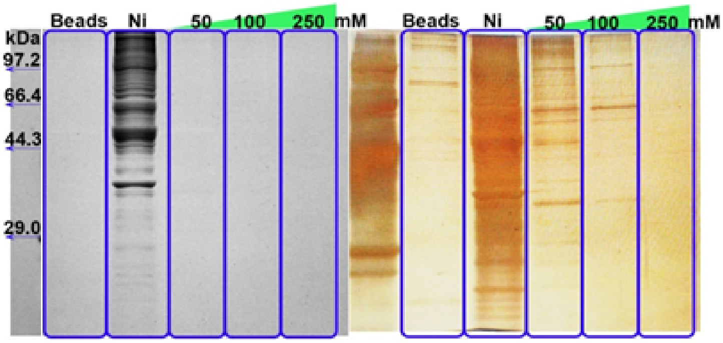

Figure S4. Evaluation of different amount of imidazole in reducing background binding proteins toward Ni-NTA beads with whole cell extract. Lane 1: Sepharose beads without Ni^{2+}, lane 2: Sepharose beads with Ni^{2+}, lane 3-5: imidazole concentration of 50mM, 100mM, 250mM respectively.

2.3 Two dimensional electrophoresis gel image and protein identification

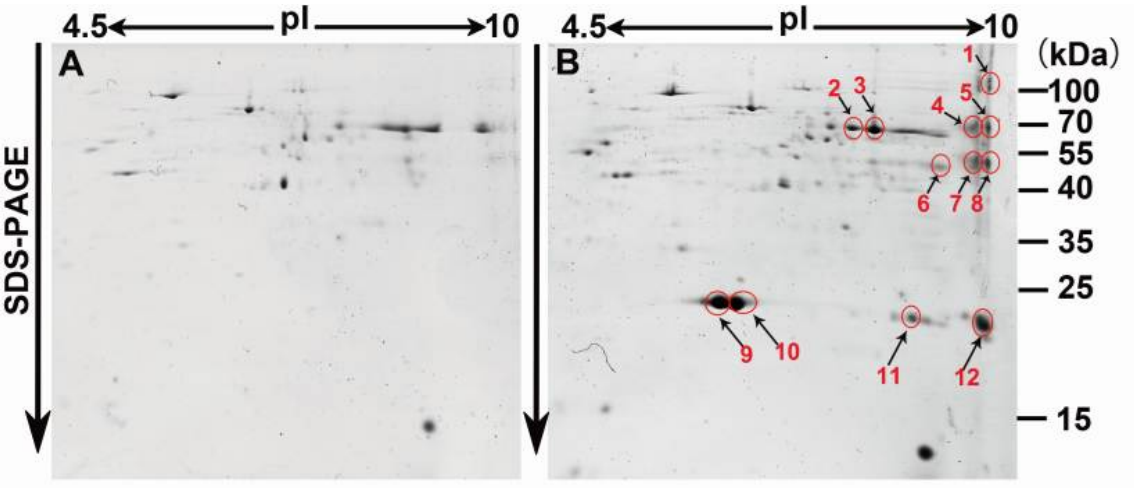

Figure S5. Native isolation of binding proteins in skov-3 cell extract using DNA probes with(B) or without (A) cisplatin crosslink. Proteins were separated on strips with pH range 4.5-10. Differential spots which were remarkably enriched on gel (B) were marked in red circles and numbered.

Table S1. Proteins identified by cisplatin containing Probe affinity isolation from Cell Extraction under native condition.

**NO.**	**Protein ID**	**Score**
**1**	SFPQ, Splicing factor, proline- and glutamine-rich, Nominal mass(Mr)76216	**82**

	Calculated pI: 9.45	
**2**	KPYM, Pyruvate kinase isozymes M1/M2, Nominal mass (Mr): 58470 Calculated pI: 7.96	**234**
**3**	KPYM, Pyruvate kinase isozymes M1/M2, Nominal mass (Mr): 58470 Calculated pI: 7.96	**282**
**4**	NONO, Non-POU domain-containing octamer-binding protein, Nominal mass (Mr): 54311 Calculated pI: 9.01	**86**
**5**	NONO, Non-POU domain-containing octamer-binding protein, Nominal mass (Mr): 54311 Calculated pI: 9.01	**66**
**6**	NONO, Non-POU domain-containing octamer-binding protein, Nominal mass (Mr): 54311 Calculated pI: 9.01	**53**
**7**	NONO, Non-POU domain-containing octamer-binding protein, Nominal mass (Mr): 54311 Calculated pI: 9.01	**114**
**8**	NONO, Non-POU domain-containing octamer-binding protein, Nominal mass (Mr): 54311 Calculated pI: 9.01	**78**
**9**	HMG1, High mobility group protein B1, Nominal mass (Mr): 25049, Calculated pI: 5.62	**79**
**10**	HMG1, High mobility group protein B1, Nominal mass (Mr): 25049, Calculated pI: 5.62	**84**
**11**	HMG2, High mobility group protein B2, Nominal mass (Mr):24190, Calculated pI: 7.62	**95**
**12**	HMG1, High mobility group protein B1, Nominal mass (Mr): 25049, Calculated pI: 5.62	**74**

2.4 Westen blot validation

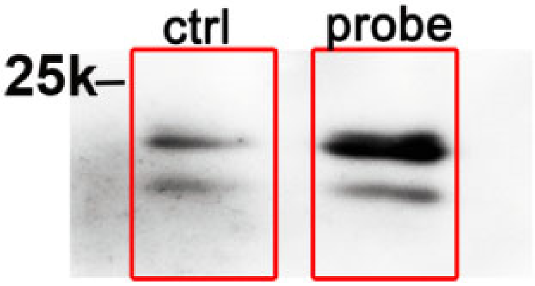

Figure S6. Validation of HMGB3 through western blot with their specific antibodies. **Ctrl** sample is isolated with probe without cisplatin lesion, and **probe** sample is isolated with probe with cisplatin modification.

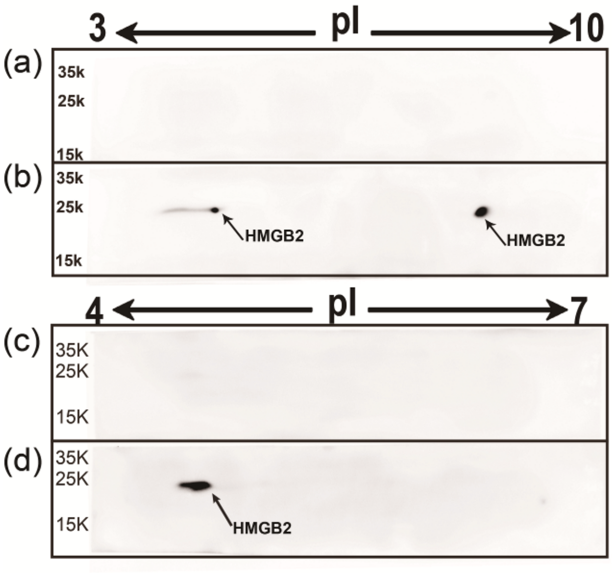

Figure S7. western blot validation of isolated HMGB2 using probe modified with cisplatin crosslink. Proteins are separated with pH range 3-10 and 4-7 respectively. Probe without cisplatin modification (a,c) are designed as control.

2.5 Post-translational modification (PTM) identification

Table S2. Post-translational modified sites of HMGB1 isomers distinguished by 2-DE.

No.	ID	Position	Modified Sequence
**1**	B	K2	GK(ac)GDPKKPR
**2**	A,B,C,D	*K11	GK(ac)MSSYAFFVQTCR
**3**	C	S14	MSS(p)YAFFVQTCR
**4**	A,B,D	*K29	K(ac)HPDASVNFSEFSK

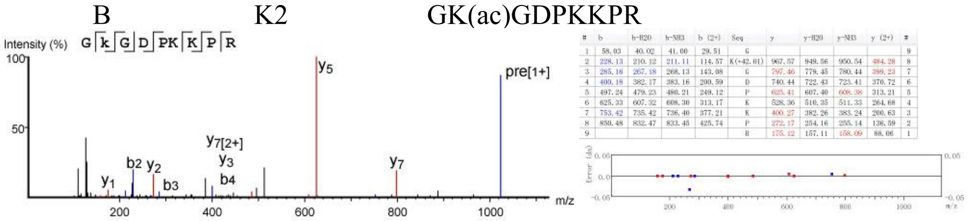

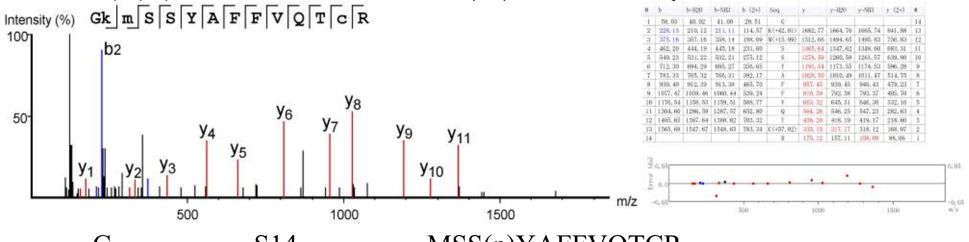

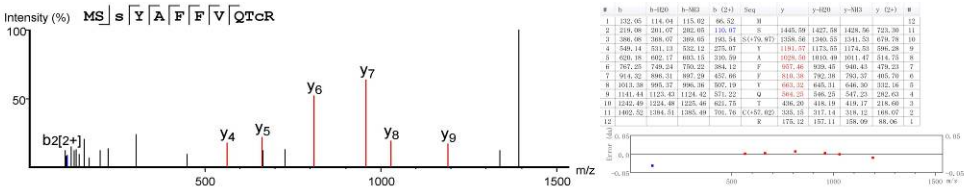

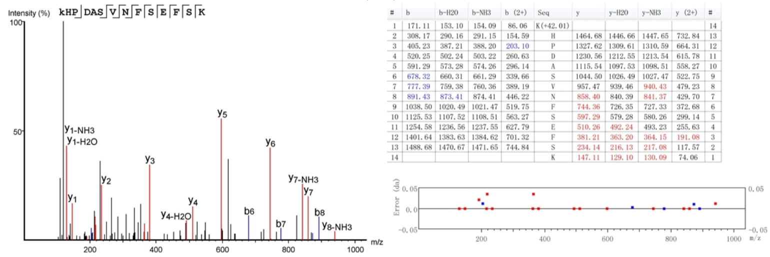

5 C,D *S34 KHPDAS(p)VNFSEFSK

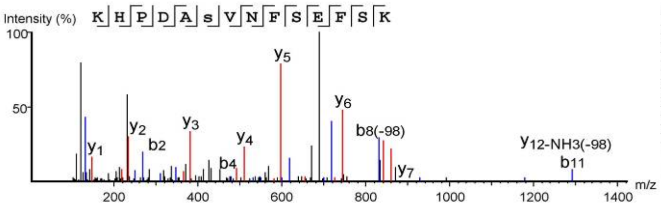

#	b	b-H2O	b-NH3	b (2+)	Seq	y	y-H2O	y-NH3	y (2+)	#
1	129.10	111.09	112.08	65.05	K					14
2	266.16	248.15	249.13	133.58	H	1544.64	1526.63	1527.61	772.82	13
3	363.21	345.20	346.14	182.11	P	1407.58	1389.57	1390.56	704.29	12
4	478.24	460.23	461.21	239.62	D	1310.53	1292.52	1293.50	655.77	11
5	549.27	531.27	532.25	275.14	A	1195.50	1177.50	1178.48	598.29	10
6	716.28	698.27	699.25	358.64	S(+79.97)	1124.47	1106.46	1107.44	562.73	9
7	815.35	797.33	798.32	408.17	V	957.47	939.46	940.44	479.23	8
8	929.39	911.38	912.36	465.19	N	858.40	840.39	841.38	429.70	7
9	1076.46	1058.45	1059.43	538.73	F	744.35	726.34	727.33	372.68	6
10	1163.49	1145.48	1146.46	582.24	S	597.29	579.28	580.26	299.14	5
11	1292.58	1274.52	1275.50	646.77	E	510.25	492.24	493.23	255.63	4
12	1439.60	1421.59	1422.57	720.30	F	381.21	363.20	364.15	191.11	3
13	1526.63	1508.62	1509.60	763.82	S	234.14	216.13	217.08	117.57	2
14					K	147.11	129.10	130.09	74.06	1

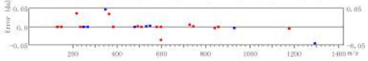

6 A,B,D *K42 KHPDASVNFSEFSK(ac)

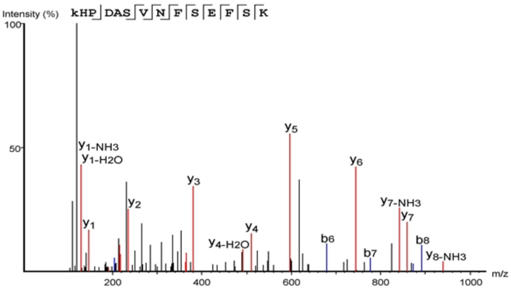

#	b	b-H2O	b-NH3	b (2+)	Seq	y	y-H2O	y-NH3	y (2+)	#
1	171.11	153.10	154.09	86.06	K(+42.01)					14
2	308.17	290.16	291.15	154.59	H	1464.68	1446.66	1447.65	732.84	13
3	405.23	387.21	388.20	203.10	P	1327.62	1309.61	1310.59	664.31	12
4	520.25	502.24	503.22	260.63	D	1230.56	1212.55	1213.54	615.78	11
5	591.29	573.28	574.26	296.14	A	1115.54	1097.53	1098.51	558.27	10
6	678.32	660.31	661.29	339.66	S	1044.50	1026.49	1027.47	522.75	9
7	777.39	759.38	760.36	389.19	V	957.47	939.46	940.43	479.23	8
8	891.43	873.41	874.41	446.22	N	858.40	840.39	841.37	429.70	7
9	1038.50	1020.49	1021.47	519.75	F	744.36	726.35	727.33	372.68	6
10	1125.53	1107.52	1108.51	563.27	S	597.29	579.28	580.26	299.14	5
11	1254.58	1236.56	1237.55	627.79	E	510.26	492.24	493.23	255.63	4
12	1401.64	1383.63	1384.62	701.32	F	381.21	363.20	364.15	191.08	3
13	1488.68	1470.67	1471.65	744.84	S	234.14	216.13	217.08	117.57	2
14					K	147.11	129.10	130.09	74.06	1

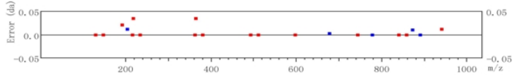

7 C S52 TMS(p)AKEK

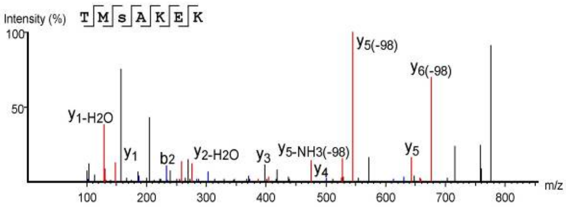

#	b	b-H2O	b-NH3	b (2+)	Seq	y	y-H2O	y-NH3	y (2+)	#
1	102.06	84.04	85.03	51.53	T					7
2	233.10	215.09	216.07	117.05	M	773.33	755.32	756.30	387.16	6
3	400.09	382.08	383.07	200.55	S(+79.97)	642.29	624.28	625.26	321.64	5
4	471.13	453.12	454.10	236.07	A	475.29	457.28	458.26	238.14	4
5	599.23	581.22	582.20	300.11	K	404.25	386.24	387.22	202.63	3
6	728.27	710.26	711.24	364.63	E	276.16	258.14	259.13	138.58	2
7					K	147.11	129.10	130.09	74.06	1

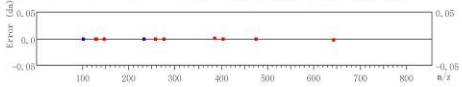

8 B,C *K54 TMSAK(ac)EK

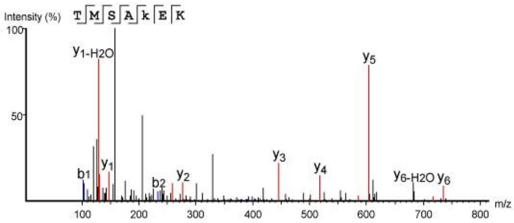

#	b	b-H2O	b-NH3	b (2+)	Seq	y	y-H2O	y-NH3	y (2+)	#
1	102.06	84.04	85.03	51.53	T					7
2	233.10	215.09	216.07	117.05	M	735.37	717.35	718.36	368.19	6
3	320.13	302.12	303.10	160.56	S	604.33	586.32	587.30	302.67	5
4	391.20	373.15	374.14	196.08	A	517.30	499.27	500.27	259.15	4
5	561.27	543.26	544.24	281.14	K(+42.01)	446.26	428.25	429.23	223.63	3
6	690.31	672.30	673.29	345.66	E	276.15	258.14	259.13	138.58	2
7					K	147.11	129.10	130.09	74.06	1

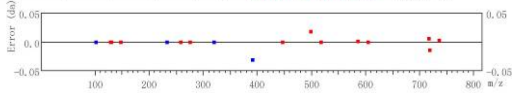

9 B *K58 GK(ac)FEDMAK

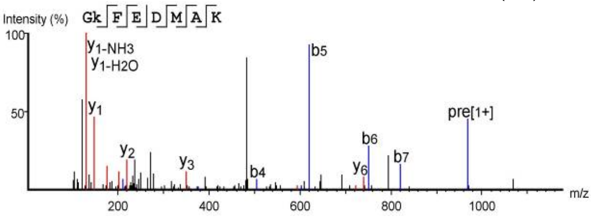

#	b	b-H2O	b-NH3	b (2+)	Seq	y	y-H2O	y-NH3	y (2+)	#
1	58.03	40.02	41.00	29.51	G					8
2	228.13	210.12	211.12	114.57	K(+42.01)	910.43	892.42	893.41	455.72	7
3	375.21	357.19	358.18	188.10	F	740.33	722.32	723.30	370.66	6
4	504.25	486.24	487.22	252.62	E	593.27	575.25	576.23	297.13	5
5	619.28	601.26	602.26	310.14	D	464.22	446.21	447.19	232.61	4
6	750.32	732.30	733.29	375.66	M	349.19	331.18	332.16	175.12	3
7	821.36	803.34	804.32	411.18	A	218.15	200.14	201.12	109.57	2
8					K	147.11	129.10	130.09	74.06	1

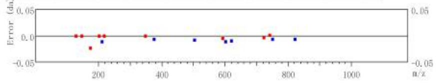

10 A K75 EMK(ac)TYIPPKGETK

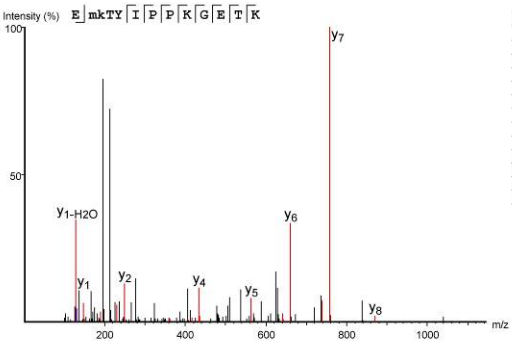

#	b	b-H2O	b-NH3	b (2+)	Seq	y	y-H2O	y-NH3	y (2+)	#
1	130.09	112.04	113.02	65.53	E					13
2	277.09	259.08	260.06	139.04	M(+15.99)	1450.76	1432.75	1433.73	725.88	12
3	447.19	429.18	430.16	224.10	K(+42.01)	1303.73	1285.72	1286.70	652.36	11
4	548.24	530.23	531.21	274.62	T	1133.62	1115.61	1116.59	567.31	10
5	711.30	693.29	694.28	356.15	Y	1032.57	1014.56	1015.55	516.79	9
6	824.39	806.38	807.36	412.69	I	869.51	851.50	852.48	435.23	8
7	921.44	903.43	904.41	461.22	P	756.42	738.41	739.42	378.71	7
8	1018.49	1000.48	1001.46	509.75	P	659.37	641.36	642.36	330.19	6
9	1146.59	1128.58	1129.56	573.79	K	562.32	544.31	545.29	281.66	5
10	1203.61	1185.60	1186.58	602.30	G	434.22	416.21	417.20	217.61	4
11	1332.65	1314.64	1315.62	666.83	E	377.20	359.19	360.18	189.09	3
12	1433.70	1415.69	1416.67	717.35	T	248.16	230.15	231.13	124.58	2
13					K	147.11	129.10	130.09	74.06	1

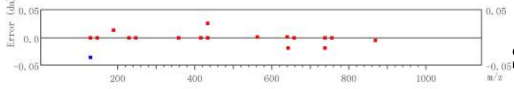

11 A,B,C,D *K81 TYIPPK(ac)GETK

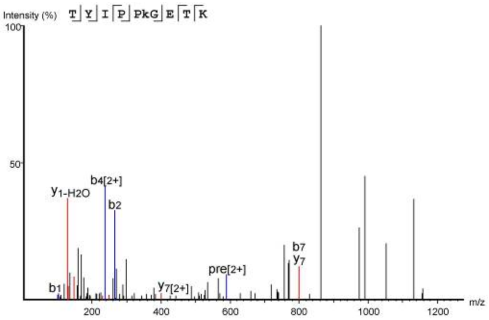

#	b	b-H2O	b-NH3	b (2+)	Seq	y	y-H2O	y-NH3	y (2+)	#
1	102.06	84.04	85.03	51.53	T					10
2	265.12	247.11	248.09	133.06	Y	1074.58	1056.57	1057.56	537.79	9
3	378.20	360.19	361.18	189.60	I	911.52	893.51	894.49	456.26	8
4	475.26	457.25	458.23	238.13	P	798.44	780.42	781.41	399.72	7
5	572.31	554.30	555.28	286.65	P	701.38	683.37	684.36	351.19	6
6	742.41	724.40	725.39	371.71	K(+42.01)	604.33	586.32	587.30	302.67	5
7	799.43	781.42	782.41	400.22	G	434.22	416.21	417.20	217.61	4
8	928.48	910.47	911.45	464.74	E	377.20	359.19	360.18	189.10	3
9	1029.53	1011.52	1012.50	515.26	T	248.16	230.15	231.13	124.58	2
10					K	147.11	129.10	130.09	74.06	1

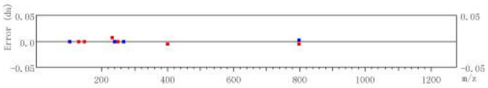

12 B K85 TYIPPKGETK(ac)K

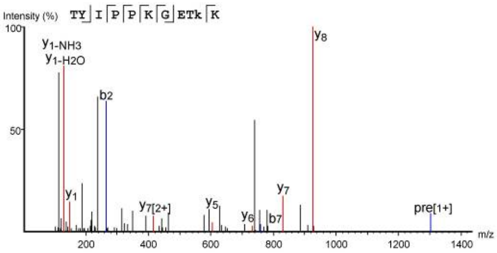

#	b	b-H2O	b-NH3	b (2+)	Seq	y	y-H2O	y-NH3	y (2+)	#
1	102.06	84.04	85.03	51.53	T					11
2	265.12	247.11	248.09	133.06	Y	1202.68	1184.67	1185.65	601.84	10
3	378.20	360.19	361.18	189.60	I	1039.61	1021.60	1022.59	520.31	9
4	475.26	457.25	458.23	238.13	P	926.55	908.52	909.50	463.77	8
5	572.31	554.30	555.28	286.65	P	829.47	811.47	812.45	415.24	7
6	700.40	682.39	683.38	350.70	K	732.41	714.41	715.40	366.71	6
7	757.43	739.41	740.40	379.21	G	604.33	586.32	587.30	302.67	5
8	886.47	868.46	869.44	443.73	E	547.31	529.30	530.28	274.15	4
9	987.52	969.50	970.49	494.26	T	418.27	400.26	401.24	209.63	3
10	1157.62	1139.61	1140.59	579.31	K(+42.01)	317.22	299.21	300.19	159.11	2
11					K	147.11	129.10	130.09	74.06	1

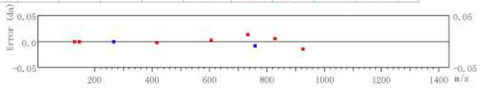

13 D K87 K(ac)FKDPNAPK

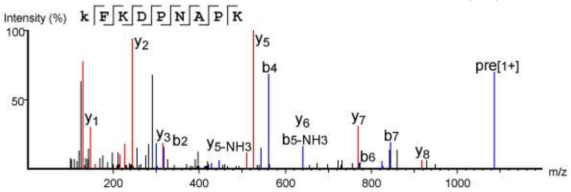

#	b	b-H2O	b-NH3	b (2+)	Seq	y	y-H2O	y-NH3	y (2+)	#
1	171.11	153.10	154.09	86.06	K(+42.01)					9
2	318.18	300.16	301.15	159.59	F	916.48	898.48	899.46	458.74	8
3	446.28	428.27	429.25	223.64	K	769.42	751.41	752.39	385.21	7
4	561.30	543.29	544.30	281.15	D	641.32	623.31	624.30	321.16	6
5	658.36	640.35	641.32	329.68	P	526.30	508.29	509.27	263.65	5
6	772.39	754.39	755.37	386.70	N	429.25	411.24	412.22	215.14	4
7	843.45	825.42	826.42	422.22	A	315.20	297.19	298.18	158.09	3
8	940.49	922.48	923.46	470.74	P	244.17	226.15	227.18	122.58	2
9					K	147.11	129.10	130.09	74.06	1

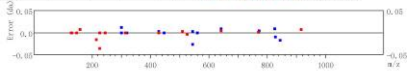

14 A,B,C K89 FK(ac)DPNAPK

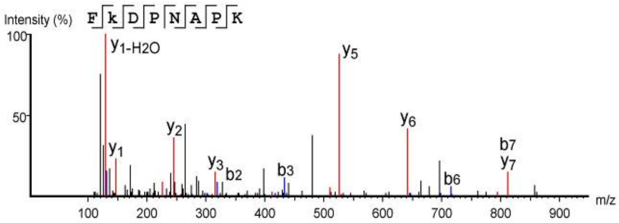

#	b	b-H2O	b-NH3	b (2+)	Seq	y	y-H2O	y-NH3	y (2+)	#
1	148.08	130.09	131.05	74.54	F					8
2	318.18	300.17	301.15	159.59	K(+42.01)	811.42	793.42	794.40	406.22	7
3	433.21	415.20	416.21	217.10	D	641.33	623.31	624.30	321.16	6
4	530.26	512.25	513.23	265.63	P	526.30	508.29	509.27	263.65	5
5	644.30	626.29	627.28	322.65	N	429.25	411.24	412.22	215.12	4
6	715.34	697.35	698.31	358.17	A	315.20	297.19	298.18	158.10	3
7	812.43	794.38	795.37	406.72	P	244.17	226.16	227.14	122.58	2
8					K	147.11	129.10	130.09	74.06	1

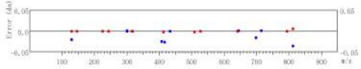

15 B K111 RPK(ac)IKGEHPGLSIGDVAK

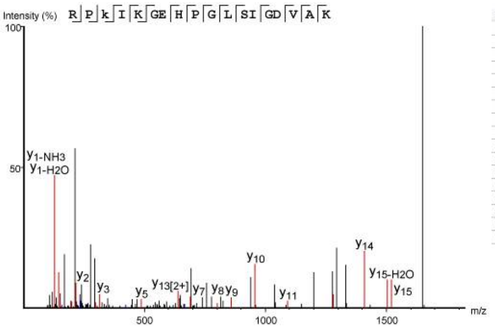

#	b	b-H2O	b-NH3	b (2+)	Seq	y	y-H2O	y-NH3	y (2+)	#
1	157.10	139.10	140.08	79.05	R					18
2	254.16	236.15	237.12	127.58	P	1788.00	1769.99	1770.97	894.50	17
3	424.24	406.26	407.24	212.63	K(+42.01)	1690.95	1672.94	1673.92	845.97	16
4	537.35	519.34	520.32	269.18	I	1520.84	1502.82	1503.82	760.92	15
5	665.41	647.44	648.42	333.22	K	1407.75	1389.75	1390.73	704.38	14
6	722.47	704.46	705.44	361.73	G	1279.65	1261.65	1262.64	640.33	13
7	851.51	833.50	834.48	426.26	E	1222.64	1204.63	1205.62	611.82	12
8	988.57	970.56	971.54	494.78	H	1093.58	1075.59	1076.57	547.30	11
9	1085.62	1067.61	1068.59	543.31	P	956.54	938.53	939.51	478.77	10
10	1142.64	1124.63	1125.62	571.82	G	859.49	841.48	842.46	430.24	9
11	1255.73	1237.72	1238.70	628.36	L	802.47	784.46	785.44	401.73	8
12	1342.76	1324.75	1325.73	671.88	S	689.38	671.37	672.36	345.19	7
13	1455.84	1437.83	1438.82	728.42	I	602.35	584.34	585.32	301.68	6
14	1512.86	1494.85	1495.84	756.93	G	489.26	471.26	472.24	245.13	5
15	1627.89	1609.88	1610.86	814.45	D	432.25	414.23	415.22	216.62	4
16	1726.96	1708.95	1709.93	863.98	V	317.22	299.21	300.19	159.09	3
17	1798.00	1779.99	1780.97	899.50	A	218.15	200.14	201.12	109.57	2

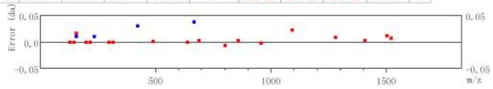

16 A,B,D K113 IK(ac)GEHPGLSIGDVAK

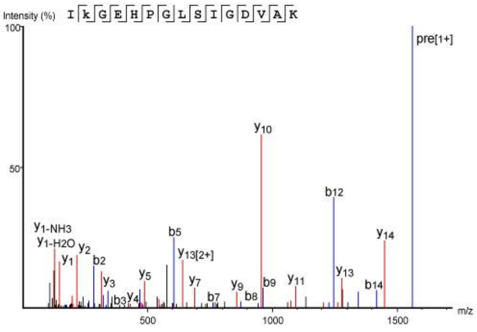

#	b	b-H2O	b-NH3	b (2+)	Seq	y	y-H2O	y-NH3	y (2+)	#
1	114.09	96.08	97.06	57.55	I					15
2	284.20	266.15	267.17	142.60	K(+42.01)	1449.76	1431.76	1432.74	725.38	14
3	341.22	323.21	324.19	171.11	G	1279.67	1261.64	1262.64	640.33	13
4	470.26	452.25	453.23	235.63	E	1222.64	1204.64	1205.62	611.82	12
5	607.32	589.31	590.29	304.16	H	1093.59	1075.59	1076.57	547.30	11
6	704.37	686.36	687.35	352.69	P	956.54	938.53	939.51	478.77	10
7	761.41	743.38	744.37	381.20	G	859.48	841.48	842.46	430.24	9
8	874.47	856.47	857.45	437.74	L	802.47	784.46	785.44	401.73	8
9	961.50	943.49	944.48	481.26	S	689.38	671.37	672.36	345.19	7
10	1074.59	1056.58	1057.57	537.80	I	602.35	584.34	585.32	301.68	6
11	1131.62	1113.61	1114.59	566.31	G	489.27	471.26	472.25	245.13	5
12	1246.64	1228.63	1229.62	623.82	D	432.24	414.23	415.22	216.62	4
13	1345.68	1327.70	1328.68	673.36	V	317.22	299.21	300.19	159.11	3
14	1416.72	1398.74	1399.72	708.87	A	218.15	200.14	201.12	109.57	2
15					K	147.11	129.10	130.09	74.06	1

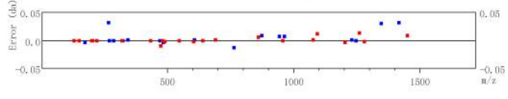

17 A,B,D *S120 IKGEHPGLS(p)IGDVAK

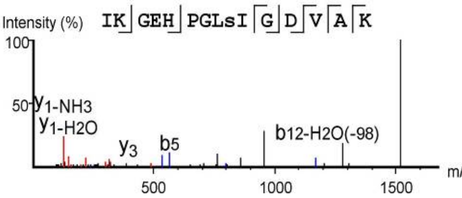

#	b	b-H20	b-NH3	b (2+)	Seq	y	y-H20	y-NH3	y (2+)	#
1	114.09	96.08	97.06	57.55	I					15
2	242.19	224.18	225.16	121.59	K	1487.73	1469.71	1470.70	744.36	14
3	299.21	281.20	282.18	150.10	G	1359.63	1341.62	1342.60	680.32	13
4	428.25	410.24	411.22	214.63	E	1302.61	1284.60	1285.58	651.80	12
5	565.31	547.30	548.28	283.15	H	1173.57	1155.56	1156.54	587.28	11
6	662.36	644.35	645.34	331.68	P	1036.51	1018.50	1019.48	518.75	10
7	719.38	701.37	702.36	360.19	G	939.45	921.44	922.43	470.23	9
8	832.47	814.46	815.44	416.73	L	882.43	864.42	865.41	441.72	8
9	999.47	981.46	982.44	500.23	S(+79.97)	769.35	751.34	752.32	385.17	7
10	1112.55	1094.54	1095.52	556.78	I	602.35	584.34	585.32	301.68	6
11	1169.57	1151.56	1152.54	585.29	G	489.27	471.26	472.24	245.13	5
12	1284.60	1266.59	1267.57	642.80	D	432.25	414.23	415.22	216.62	4
13	1383.67	1365.66	1366.64	692.33	V	317.22	299.21	300.16	159.09	3
14	1454.70	1436.69	1437.68	727.85	A	218.15	200.14	201.12	109.57	2
15					K	147.11	129.10	130.09	74.06	1

18 A,B,C,D K127 K(ac)LGEMWNNTAADDKQPYEK

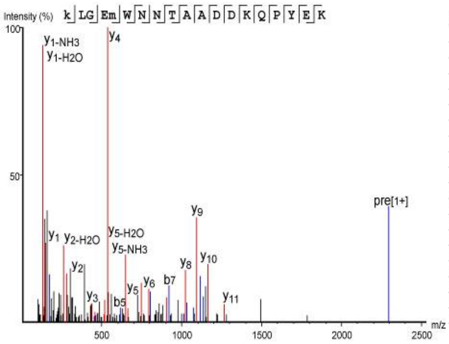

#	b	b-H20	b-NH3	b (2+)	Seq	y	y-H20	y-NH3	y (2+)	#
1	171.11	153.10	154.09	86.06	K(+42.01)					19
2	284.20	266.19	267.17	142.60	L	2125.95	2107.94	2108.92	1063.47	18
3	341.22	323.21	324.19	171.11	G	2012.87	1994.85	1995.84	1006.93	17
4	470.26	452.25	453.23	235.63	E	1955.84	1937.83	1938.82	978.42	16
5	617.31	599.29	600.27	309.15	M(+15.99)	1826.80	1808.79	1809.77	913.90	15
6	803.40	785.37	786.35	402.19	W	1679.77	1661.76	1662.74	840.38	14
7	917.41	899.41	900.39	459.17	N	1493.69	1475.68	1476.66	747.34	13
8	1031.46	1013.43	1014.44	516.23	N	1379.64	1361.63	1362.62	690.32	12
9	1132.52	1114.49	1115.48	566.75	T	1265.58	1247.59	1248.57	633.30	11
10	1203.55	1185.54	1186.52	602.27	A	1164.54	1146.54	1147.53	582.78	10
11	1274.58	1256.57	1257.56	637.79	A	1093.52	1075.49	1076.49	547.26	9
12	1389.61	1371.60	1372.58	695.31	D	1022.47	1004.47	1005.45	511.74	8
13	1504.64	1486.63	1487.61	752.82	D	907.44	889.44	890.42	454.23	7
14	1632.73	1614.72	1615.71	816.87	K	792.42	774.41	775.40	396.71	6
15	1760.79	1742.78	1743.76	880.90	Q	664.33	646.32	647.31	332.67	5
16	1857.84	1839.83	1840.82	929.42	P	536.27	518.26	519.25	268.64	4
17	2020.91	2002.90	2003.88	1010.95	Y	439.22	421.21	422.19	220.11	3
18	2149.95	2131.94	2132.92	1075.49	E	276.15	258.14	259.13	138.58	2
19					K	147.11	129.10	130.07	74.06	1

19 D T135 KLGEMWNNT(p)AADDKQPYEK

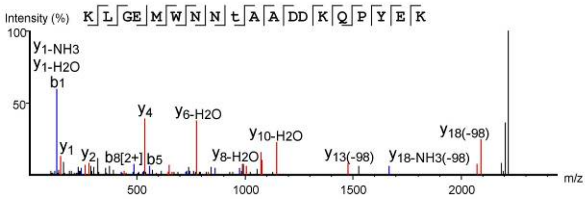

#	b	b-H20	b-NH3	b (2+)	Seq	y	y-H20	y-NH3	y (2+)	#
1	129.10	111.09	112.08	65.05	K					19
2	242.19	224.18	225.16	121.59	L	2189.92	2171.91	2172.89	1095.46	18
3	299.21	281.20	282.18	150.10	G	2076.84	2058.83	2059.81	1038.92	17
4	428.25	410.24	411.22	214.63	E	2019.82	2001.80	2002.79	1010.41	16
5	559.29	541.28	542.26	280.15	M	1890.77	1872.76	1873.75	945.89	15
6	745.37	727.36	728.34	373.19	W	1759.73	1741.72	1742.71	880.37	14
7	859.41	841.40	842.39	430.21	N	1573.65	1555.64	1556.63	787.33	13
8	973.44	955.45	956.43	487.23	N	1459.61	1441.60	1442.58	730.30	12
9	1154.47	1136.46	1137.44	577.74	T(+79.97)	1345.57	1327.56	1328.54	673.28	11
10	1225.51	1207.50	1208.48	613.25	A	1164.55	1146.54	1147.53	582.78	10
11	1296.54	1278.53	1279.52	648.77	A	1093.52	1075.51	1076.50	547.26	9
12	1411.57	1393.56	1394.54	706.29	D	1022.48	1004.46	1005.45	511.74	8
13	1526.60	1508.59	1509.57	763.80	D	907.45	889.44	890.42	454.23	7
14	1654.69	1636.68	1637.67	827.85	K	792.42	774.40	775.40	396.71	6
15	1782.75	1764.74	1765.73	891.88	Q	664.32	646.31	647.30	332.67	5
16	1879.80	1861.79	1862.78	940.40	P	536.27	518.26	519.24	268.64	4
17	2042.87	2024.86	2025.84	1021.93	Y	439.22	421.21	422.19	220.11	3

20 B,C *K156 YEK(ac)DIAAYR

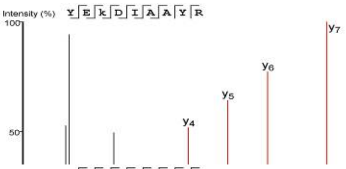

#	b	b-H20	b-NH3	b (2+)	Seq	y	y-H20	y-NH3	y (2+)	#
1	164.07	146.06	147.04	82.54	Y					9
2	293.11	275.10	276.09	147.04	E	1007.52	989.50	990.51	504.26	8
3	463.22	445.21	446.19	232.11	K(+42.01)	878.47	860.46	861.45	439.74	7
4	578.24	560.24	561.22	289.62	D	708.37	690.36	691.34	354.68	6
5	691.34	673.32	674.30	346.17	I	593.34	575.33	576.31	297.17	5
6	762.39	744.36	745.34	381.68	A	480.26	462.25	463.22	240.63	4
7	833.40	815.39	816.38	417.20	A	409.22	391.21	392.19	205.11	3
8	996.47	978.46	979.44	498.73	Y	338.18	320.17	321.15	169.59	2
9					R	175.12	157.11	158.09	88.06	1

21

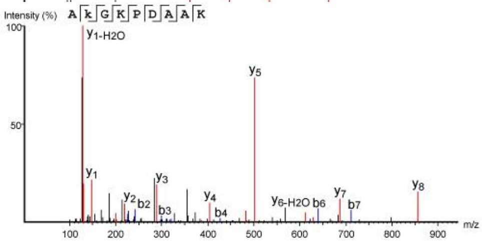

#	b	b-H20	b-NH3	b (2+)	Seq	y	y-H20	y-NH3	y (2+)	#
1	72.04	54.03	55.02	36.52	A					9
2	242.15	224.14	225.12	121.58	K(+42.01)	856.49	838.48	839.46	428.74	8
3	299.17	281.16	282.14	150.09	G	686.38	668.37	669.36	343.69	7
4	427.26	409.26	410.24	214.13	K	629.36	611.35	612.33	315.18	6
5	524.32	506.31	507.29	262.66	P	501.27	483.25	484.26	251.13	5
6	639.34	621.33	622.34	320.18	D	404.21	386.20	387.19	202.61	4
7	710.38	692.37	693.36	355.69	A	289.19	271.18	272.16	145.09	3
8	781.42	763.41	764.39	391.21	A	218.15	200.14	201.12	109.57	2
9					K	147.11	129.10	130.09	74.06	1

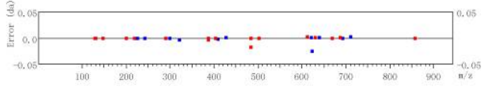

22 B,C,D K166 GK(ac)PDAAK

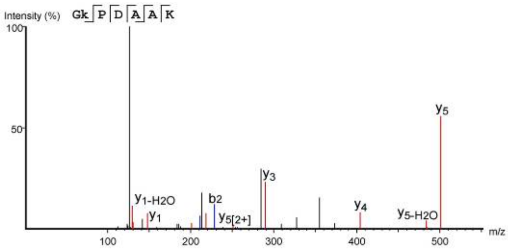

#	b	b-H20	b-NH3	b (2+)	Seq	y	y-H20	y-NH3	y (2+)	#
1	58.03	40.02	41.00	29.51	G					7
2	228.13	210.12	211.11	114.57	K(+42.01)	671.37	653.36	654.35	336.19	6
3	325.19	307.18	308.16	163.09	P	501.27	483.26	484.26	251.14	5
4	440.21	422.20	423.19	220.61	D	404.21	386.20	387.19	202.61	4
5	511.25	493.24	494.22	256.13	A	289.19	271.18	272.16	145.09	3
6	582.29	564.28	565.26	291.64	A	218.15	200.14	201.12	109.57	2
7					K	147.11	129.10	130.09	74.06	1

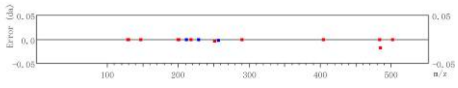

**23**	C	K176	GVVK(ac)AEK

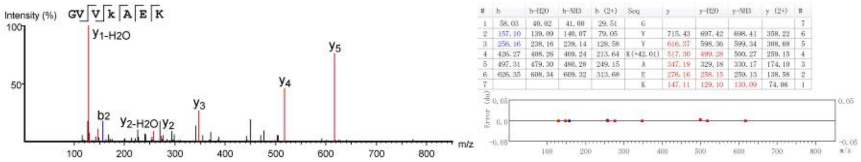

#	b	b-H2O	b-NH3	b (2+)	Seq	y	y-H2O	y-NH3	y (2+)	#
1	58.03	40.02	41.00	29.51	G					7
2	157.10	139.09	140.07	79.05	V	715.43	697.42	698.41	358.22	6
3	256.16	238.16	239.14	128.58	V	616.37	598.36	599.34	308.68	5
4	426.27	408.26	409.24	213.64	K(+42.01)	517.30	499.28	500.27	259.15	4
5	497.31	479.30	480.28	249.15	A	347.19	329.18	330.17	174.10	3
6	626.35	608.34	609.32	313.68	E	276.16	258.15	259.13	138.58	2
7					K	147.11	129.10	130.09	74.06	1

*Known modification sites

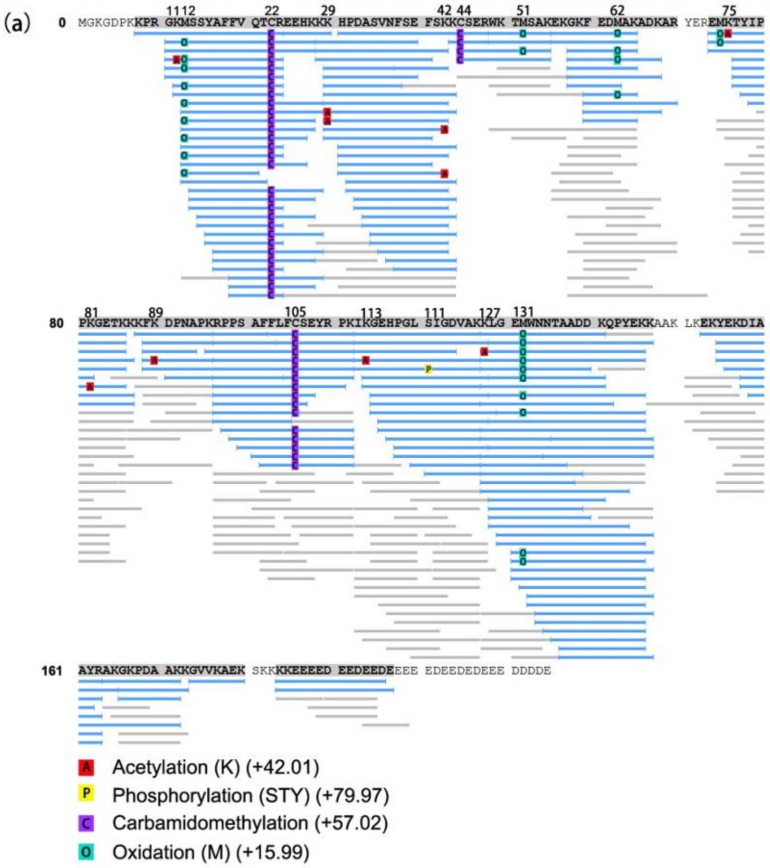

(b)

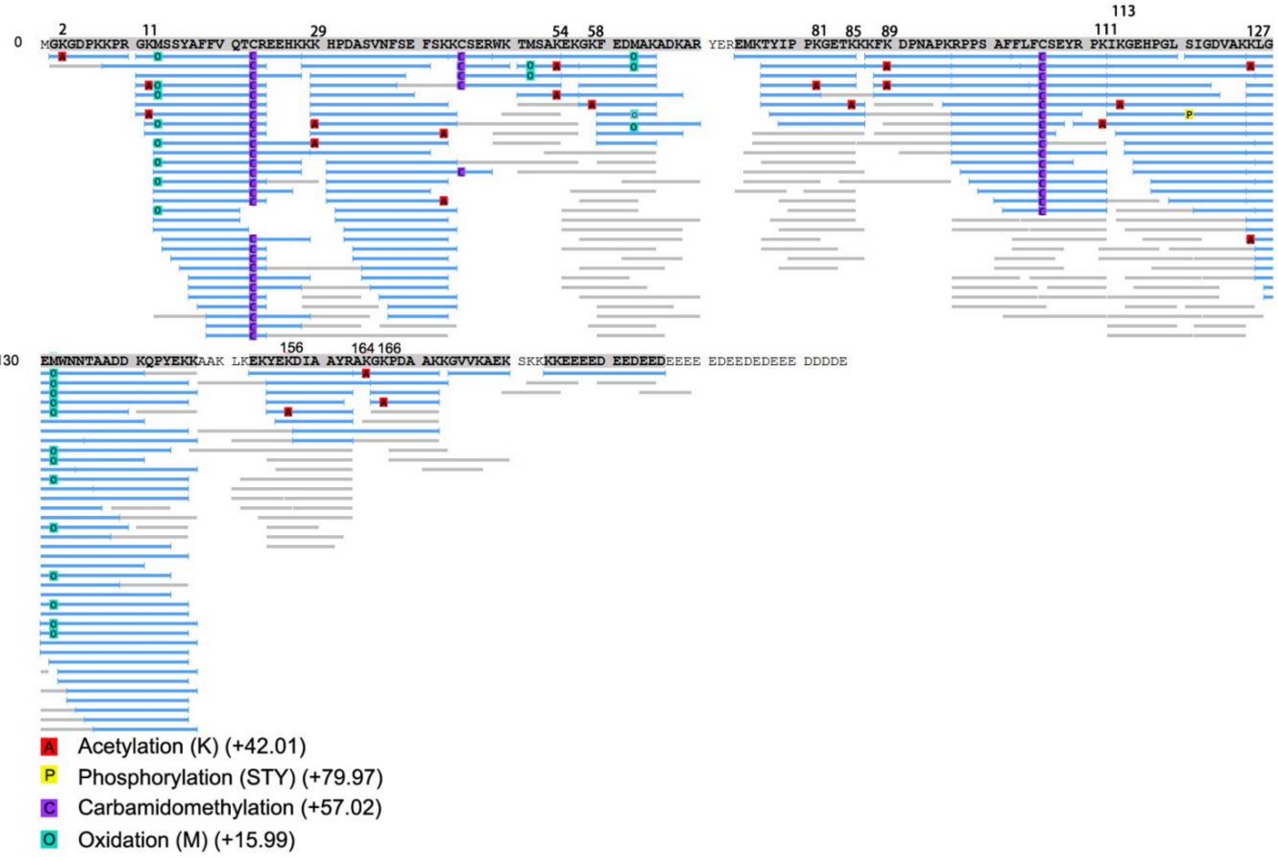

Acetylation (K) (+42.01)
Phosphorylation (STY) (+79.97)
Carbamidomethylation (+57.02)
Oxidation (M) (+15.99)

(c)

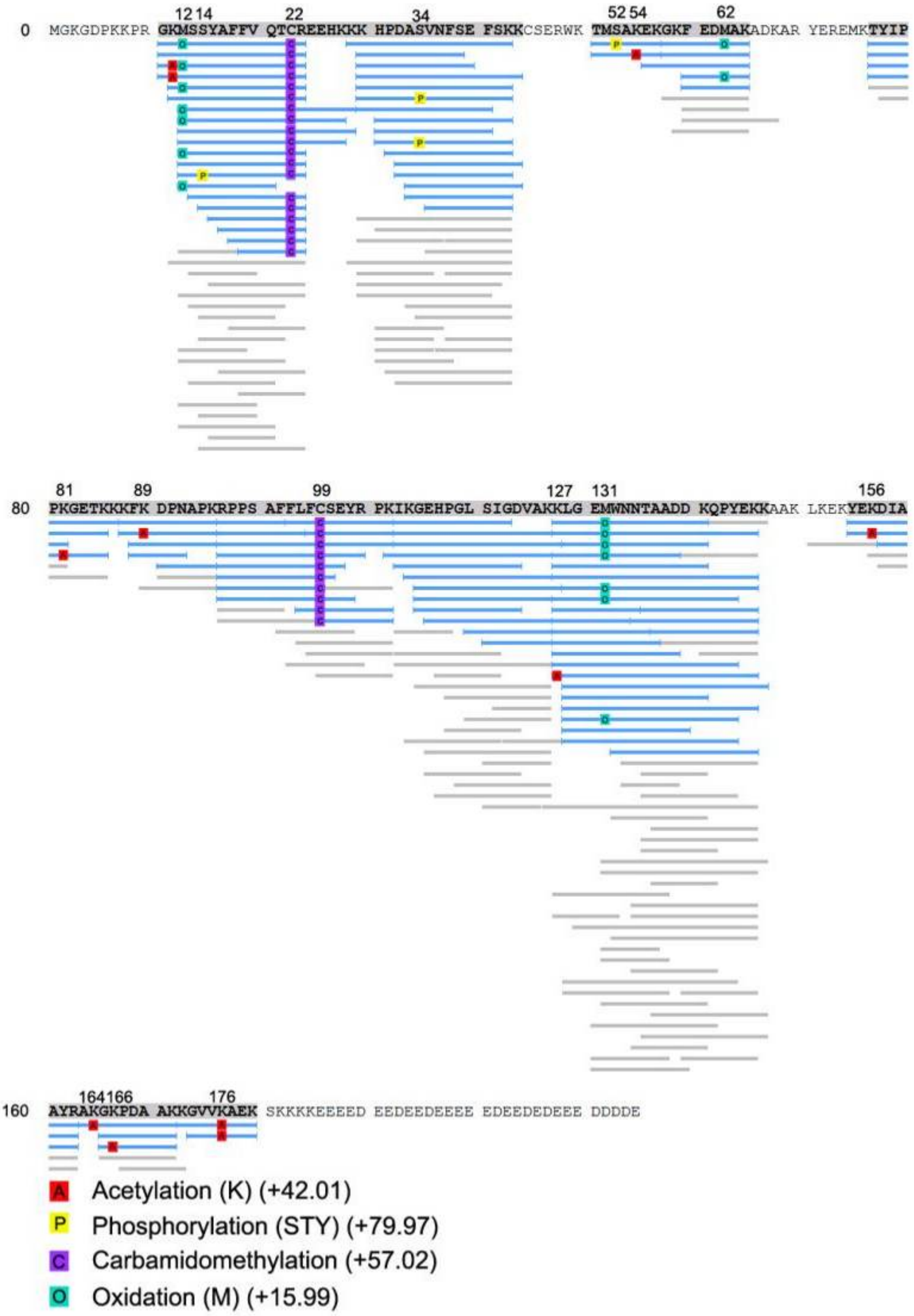

0 MGKGDPKKPR GKMSSYAFFV QTCREEHKKK HPDASVNFSE FSKKCSERWK TMSAKEKGKF EDMAKADKAR YEREMKTYIP
12 14
22
34
52 54
62
80 PKGETKKKFK DPNAPKRPPS AFFLFCSEYR PKIKGEHPGL SIGDVAKKLG EMWNNTAADD KQPYEKKAAK LKEKYEKDIA
81
89
99
127
131
156
160 AYRAKGKPDA AKKGVVKAEK SKKKKEEEED EEDEEDEEEE EDEEDEDEEE DDDDE
164166
176
A Acetylation (K) (+42.01)
P Phosphorylation (STY) (+79.97)
C Carbamidomethylation (+57.02)
O Oxidation (M) (+15.99)

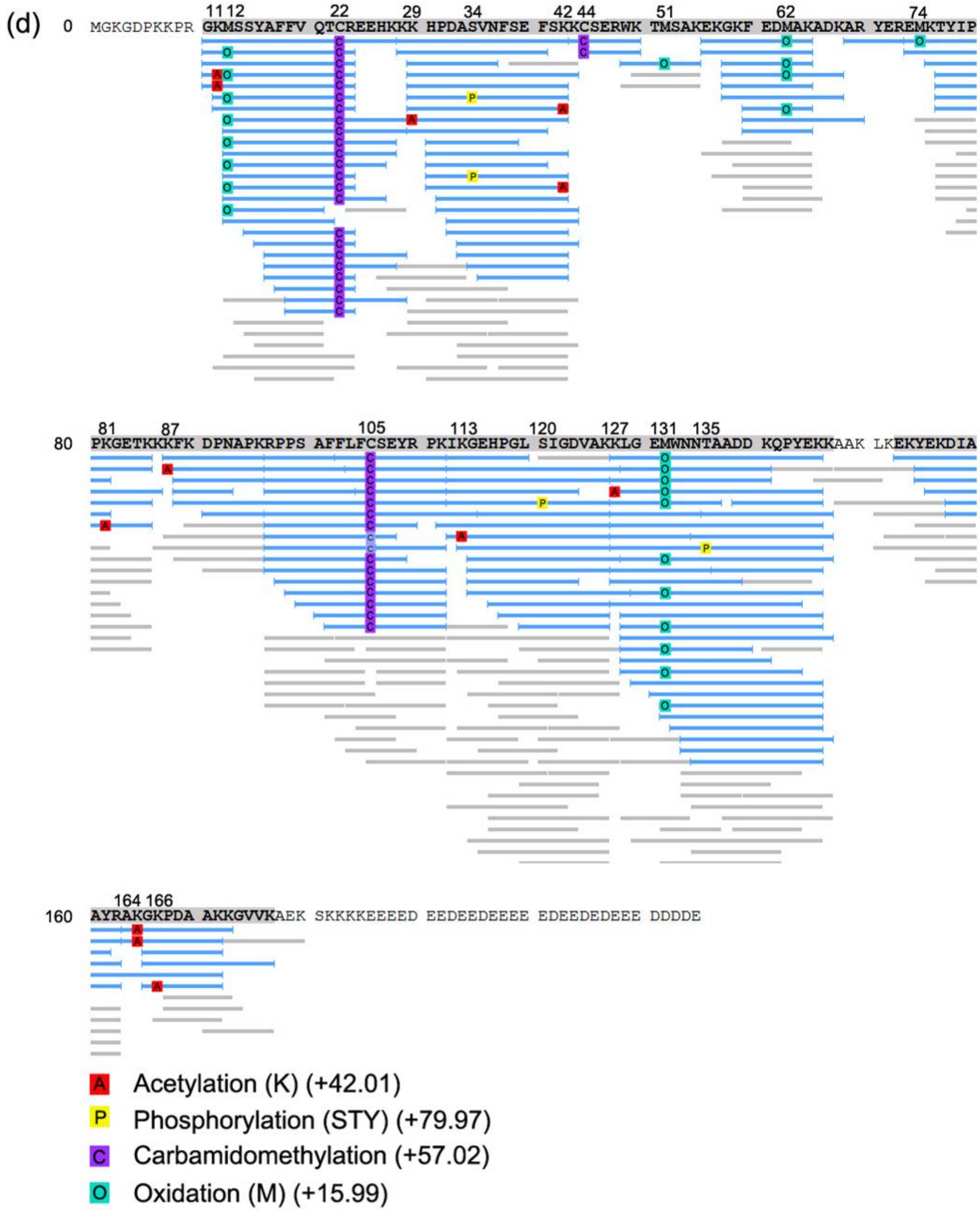

Figure S8. Graphic presentation of sequence assignment for LC-MS/MS data. HMGB1 PTM isomer A(a), B(b), C(c), D(d) are separated by isoelectric focusing and digested into peptides by trypsin respectively.

2.6 Raw LC-MS/MS data for PTM profiling of HMGB1 subform A

Peptide	Unique	-10lgP	Mass	ppm	m/z	Z	RT	PTM
K.RPPSAFFLFC(+57.02)SEYRPK.I	Y	132.56	2000.993	1.9	501.2565	4	72.48	Y
R.GKM(+15.99)SSYAFFVQTC(+57.02)REEHK.K	Y	115.25	2220.009	0.2	741.0106	3	34.9	Y
K.HPDASVNFSEFSKKC(+57.02)SER.W	Y	107.73	2123.97	0	531.9996	4	34.22	Y
K.KLGEMWNNTAADDKQPYEK.K	Y	105.97	2237.042	2.9	746.6901	3	54.5	N

K.M(+15.99)SSYAFFVQTC(+57.02)REEHK.K	Y	105.4	2034.893	1.4	1018.455	2	47.72	Y
K.LGEMWNNTAADDKQPYEK.K	Y	104.97	2108.947	1.5	703.9907	3	43.98	N
K.KLGEM(+15.99)WNNTAADDKQPYEK.K	Y	102.91	2253.037	6.4	564.2701	4	40.21	Y
K.LGEM(+15.99)WNNTAADDKQPYEK.K	Y	101.49	2124.942	0.5	709.3217	3	36.73	Y
K.MSSYAFFVQTC(+57.02)R.E	Y	98.98	1495.659	1.6	748.8379	2	68.77	Y
K.GEHPGLSIGDVAKK.L	Y	97.29	1406.752	0.9	704.3837	2	27.07	N
P.PSAFFLFC(+57.02)SEYRPK.I	Y	95.81	1747.839	2.8	583.6219	3	68.23	Y
R.GKM(+15.99)SSYAFFVQTC(+57.02)R.E	Y	94.08	1696.77	0.3	849.3926	2	49.84	Y
K.HPDASVNFSEFSK.K	Y	93.15	1463.668	-0.5	732.8409	2	60.01	N
L.GEMWNNTAADDKQPYEKK.A	Y	92.89	2123.958	-0.9	708.9927	3	27.04	N
K.KHPDASVNFSEFSKK.C	Y	92.61	1719.858	1.4	430.9724	4	31.59	N
R.GKMSSYAFFVQTC(+57.02)R.E	Y	92.14	1680.775	0.9	841.3957	2	55.6	Y
K.KHPDASVNFSEFSK.K	Y	91.64	1591.763	2	531.596	3	73.85	N
K.M(+15.99)SSYAFFVQTC(+57.02)REEHKK.K	Y	91.15	2162.988	-1.1	541.7536	4	36.91	Y
R.PPSAFFLFC(+57.02)SEYRPK.I	Y	90.73	1844.892	3.4	615.9733	3	69.27	Y
K.GEHPGLSIGDVAK.K	Y	90.47	1278.657	-0.6	640.3353	2	27.19	N
K.IKGEHPGLSIGDVAKK.L	Y	90.23	1647.931	2	412.9908	4	22.87	N
G.EMWNNTAADDKQPYEK.K	Y	90.17	1938.842	-0.6	647.2875	3	33.98	N
K.MSSYAFFVQTC(+57.02)REEHK.K	Y	89.75	2018.898	1.9	673.9745	3	51.96	Y
H.PDASVNFSEFSKK.C	Y	87.6	1454.704	1.3	485.9093	3	43.04	N
K.IKGEHPGLSIGDVAK.K	Y	87.53	1519.836	0.8	507.6196	3	36.72	N
K.M(+15.99)SSYAFFVQTC(+57.02)R.E	Y	87.33	1511.654	2.5	756.836	2	77.01	Y
K.IK(+42.01)GEHPGLSIGDVAK.K	Y	86.37	1561.846	0	781.9304	2	41.97	Y
K.EKYEKDIAAYR.A	Y	85.65	1384.699	0.2	693.3567	2	24.71	N
K.KPRGKMSSYAFFVQTC(+57.02)R.E	Y	85.22	2062.024	1.2	688.3494	3	41.99	Y
S.SYAFFVQTC(+57.02)REEHK.K	Y	84.41	1800.825	-0.1	901.4199	2	41.8	Y
K.MSSYAFFVQTC(+57.02)REEHKK.K	Y	84.03	2146.993	2.2	716.6731	3	54.25	Y
K.KLGEMWNNTAADDK.Q	Y	83.98	1591.73	-0.5	796.8718	2	34.6	N
N.NTAADDKQPYEK.K	Y	83.74	1378.637	-0.7	690.325	2	11.17	N
R.EMKTYIPPKGETK.K	Y	82.75	1520.791	-1.4	761.4016	2	19.62	N
N.NTAADDKQPYEKK.A	Y	82.59	1506.731	-6.4	754.3682	2	10.06	N
K.TYIPPKGETK.K	Y	82.37	1132.613	-0.9	567.3132	2	74.38	N
K.LGEM(+15.99)WNNTAADDKQPYEKK.A	Y	82.21	2253.037	0.9	752.0203	3	26.61	Y
S.AFFLFC(+57.02)SEYRPK.I	Y	81.71	1563.754	0.2	782.8846	2	64.88	Y
G.KMSSYAFFVQTC(+57.02)R.E	Y	81.59	1623.754	2	542.2596	3	55.28	Y
E.MWNNTAADDKQPYEK.K	Y	81.38	1809.799	-0.9	905.906	2	27.04	N
K.FKDPNAPKRPPSAFFLFC(+57.02)SEYRPK.I	Y	81.27	2898.464	1.4	725.6243	4	62.66	Y
G.EHPGLSIGDVAK.K	Y	81.24	1221.635	1	611.8255	2	34.87	N
K.LGEMWNNTAADDK.Q	Y	81.13	1463.635	0.5	732.8252	2	40.22	N
K.LGEM(+15.99)WNNTAADDK.Q	Y	80.84	1479.63	0.2	740.8224	2	29.32	Y
K.YEKDIAAYRAK.G	Y	79.15	1326.693	3.2	664.356	2	19.94	N
G.EHPGLSIGDVAKK.L	Y	78.77	1349.73	0.9	675.873	2	27.14	N
K.KLGEM(+15.99)WNNTAADDK.Q	Y	78.16	1607.725	0.6	804.8702	2	26.65	Y

N.TAADDKQPYEKK.A	Y	77.63	1392.689	-0.5	697.3511	2	9.91	N
R.EM(+15.99)KTYIPPKGETK.K	Y	77.44	1536.786	0.8	769.4008	2	14.11	Y
S.SYAFFVQTC(+57.02)R.E	Y	77.22	1277.586	1	639.8011	2	55.91	Y
R.GKMSSYAFFVQTC(+57.02)REEHK.K	Y	77.2	2204.014	1.3	552.0115	4	46.63	Y
W.NNTAADDKQPYEKK.A	Y	76.77	1620.774	-0.1	541.2653	3	10.15	N
K.LGEMWNNTAADDKQPYEKK.A	Y	76.71	2237.042	1	560.2684	4	35.76	N
K.YEKDIAAYR.A	Y	76.17	1127.561	0.5	376.8611	3	25.24	N
Y.AFFVQTC(+57.02)REEHKK.K	Y	74.44	1678.825	-2.3	840.4178	2	21.96	Y
H.PDASVNFSEFSK.K	Y	73.86	1326.609	3.8	664.3143	2	51.38	N
K.TYIPPK.G	Y	73.69	717.4061	0.3	359.7104	2	19.38	N
K.RPPSAFFLFC(+57.02)SEYRP.K	Y	72.65	1872.898	2.5	625.3082	3	69.93	Y
M.WNNTAADDKQPYEK.K	Y	72.62	1678.759	3.1	840.3892	2	22.39	N
G.KM(+15.99)SSYAFFVQTC(+57.02)R.E	Y	72.18	1639.749	1.2	547.5908	3	49.6	Y
G.EM(+15.99)WNNTAADDKQPYEK.K	Y	72.14	1954.837	1.3	652.6204	3	26.53	Y
Y.AFFVQTC(+57.02)R.E	Y	71.76	1027.491	0	514.7527	2	38.37	Y
P.SAFFLFC(+57.02)SEYRPK.I	Y	71.75	1650.786	-2.4	826.3984	2	65.42	Y
K.FEDMAKADKAR.Y	Y	71.62	1280.618	-2.1	641.3151	2	15.84	N
L.GEMWNNTAADDKQPYEK.K	Y	71.04	1995.863	6	666.299	3	34.21	N
G.EM(+15.99)WNNTAADDKQPYEKK.A	Y	70.94	2082.932	3.2	695.32	3	22.75	Y
K.TYIPPKGETKK.K	Y	68.39	1260.708	0.3	316.1843	4	16.23	N
K.K(+42.01)HPDASVNFSEFSKK.C	Y	67.87	1761.869	-1.2	441.4739	4	34.9	Y
K.K(+42.01)LGEM(+15.99)WNNTAADDKQPYEK.K	Y	67.4	2295.048	-0.6	766.0227	3	40.61	Y
M.SSYAFFVQTC(+57.02)R.E	Y	67.02	1364.618	1.2	683.3172	2	56.12	Y
G.EMWNNTAADDKQPYEKK.A	Y	66.36	2066.937	0.9	1034.477	2	27	N
S.VNFSEFSK.K	Y	65.9	956.4603	0	479.2374	2	41.6	N
S.YAFFVQTC(+57.02)R.E	Y	65.67	1190.554	0.8	596.2849	2	54.19	Y
K.MSSYAFFVQTC(+57.02)REEH.K	Y	65.51	1890.803	2.6	946.4111	2	56.29	Y
Y.AFFVQTC(+57.02)REEHK.K	Y	65.37	1550.73	-2	776.3707	2	25.4	Y
M.WNNTAADDKQPYEKK.A	Y	65.3	1806.854	1.1	904.4351	2	18.43	N
P.DASVNFSEFSKK.C	Y	65.04	1357.651	2.1	679.8344	2	27.91	N
K.RPPSAFFLFC(+57.02).S	Y	64.37	1240.606	-0.1	621.3103	2	72.11	Y
P.KRPPSAFFLFC(+57.02)SEYRPK.I	Y	63.84	2129.088	0.7	710.7037	3	61.6	Y
D.ASVNFSEFSK.K	Y	63.4	1114.529	1.3	558.2727	2	46.21	N
L.GEM(+15.99)WNNTAADDKQPYEK.K	Y	63.27	2011.858	0	671.6266	3	26.86	Y
F.FLFC(+57.02)SEYRPK.I	Y	62.96	1345.649	1.6	673.8328	2	49.26	Y
K.DIAAYR.A	Y	62.59	707.3602	1.2	354.6878	2	21.45	N
K.GKFEDMAK.A	Y	62.45	924.4375	0.5	309.1533	3	13.69	N
K.KKHPDASVNFSE.F	Y	61.98	1357.663	0.1	679.8386	2	19.49	N
K.K(+42.01)HPDASVNFSEFSK.K	Y	61.96	1633.774	-1.1	545.5978	3	42.31	Y
K.KHPDASVNF.S	Y	61.9	1013.493	1.4	507.7545	2	23.2	N
F.LFC(+57.02)SEYRPK.I	Y	61.84	1198.58	0.5	600.2978	2	26.99	Y
K.FKDPNAPK.R	Y	61.45	915.4814	1.8	458.7488	2	21.25	N
T.AADDKQPYEK.K	Y	61.17	1163.546	0.2	582.7803	2	10.77	N

K.RPPSAF.F	Y	60.71	673.3547	0.9	337.6849	2	19.12	N
P.DASVNFSEFSK.K	Y	60.35	1229.556	1	615.7861	2	75.4	N
K.GKFEDMAKADK.A	Y	60	1238.596	1.5	413.8734	3	19.61	N
K.EKGKFEDMAK.A	Y	59.94	1181.575	-1	591.7941	2	12.26	N
D.ASVNFSEFSKK.C	Y	59.52	1242.624	-1.2	622.3187	2	35.92	N
A.FFLFC(+57.02)SEYRPK.I	Y	59.47	1492.717	-1.2	747.365	2	65.03	Y
F.FVQTC(+57.02)REEHKK.K	Y	58.47	1460.719	0.3	487.9138	3	9.3	Y
A.SVNFSEFSKK.C	Y	57.87	1171.587	-0.1	586.8008	2	34.56	N
A.SVNFSEFSK.K	Y	57.85	1043.492	0.2	522.7535	2	43.94	N
K.EKGKFEDM(+15.99)AK.A	Y	57.76	1197.57	-0.3	599.7921	2	10.99	Y
K.FEDMAKADK.A	Y	57.71	1053.48	0.6	527.7476	2	16.08	N
K.DPNAPK.R	Y	57.14	640.318	-0.8	321.166	2	12.39	N
K.FEDMAK.A	Y	56.91	739.321	-0.7	370.6675	2	16.84	N
K.IKGEHPGLSIGD.V	Y	56.8	1221.635	0.5	611.8252	2	27.06	N
K.FK(+42.01)DPNAPK.R	Y	54.99	957.4919	1.7	479.754	2	19.89	Y
K.DIAAYRAKGKPDAAK.K	Y	54.38	1573.858	1.6	787.9373	2	15.78	N
K.KFKDPNAPK.R	Y	54.3	1043.576	-1.9	348.8654	3	12.35	N
H.PGLSIGDVAK.K	Y	54.16	955.5338	1.7	478.775	2	40.83	N
K.GKFEDM(+15.99)AKADK.A	Y	54.05	1254.591	2.3	628.3044	2	11.18	Y
L.FC(+57.02)SEYRPK.I	Y	53.7	1085.496	1	543.756	2	19.03	Y
K.KLGEMWNNTAADDKQ.P	Y	53.67	1719.789	-1.1	860.9006	2	34.53	N
M.SSYAFFVQTC(+57.02)REEHKK.K	Y	53.63	2015.952	-0.5	504.9951	4	34.57	Y
K.KKHPDASVNFSEF.S	Y	53.55	1504.731	0.5	502.5845	3	39.73	N
K.KHPDASVNFSEF.S	Y	53.07	1376.636	1.5	689.3263	2	50.09	N
K.KHPDASVNFSEFSK(+42.01).K	Y	52.77	1633.774	-0.2	545.5983	3	47.19	Y
K.KKHPDASVNFSEFSK.K	Y	52.25	1719.858	2.2	574.2945	3	25.44	N
E.HPGLSIGDVAK.K	Y	51.63	1092.593	0.3	547.3038	2	31.23	N
S.YAFFVQTC(+57.02)REEHKK.K	Y	51.52	1841.888	-0.9	614.9694	3	27.55	Y
N.FSEFSKK.C	Y	50.65	871.4439	0.8	436.7296	2	25.33	N
N.TAADDKQPYEK.K	Y	50.22	1264.594	-0.6	633.3036	2	38.51	N
K.RPPSAFFLFC(+57.02)SE.Y	Y	50.14	1456.681	0.7	729.3482	2	71.1	Y
T.AADDKQPYEKK.A	Y	49.42	1291.641	-1.2	646.8268	2	33.44	N
R.WKTMSAK.E	Y	49.4	850.4371	0.3	426.226	2	13.4	N
K.KLGEMWNN.T	Y	48.9	990.4593	1.4	496.2376	2	36.7	N
F.FVQTC(+57.02)R.E	Y	48.53	809.3854	-0.2	405.6999	2	11.68	Y
K.KLGEMWNNTAA.D	Y	48.53	1233.581	0.8	617.7983	2	42.7	N
K.LGEMWNNTAADDKQPY.E	Y	48.41	1851.81	0.3	926.9124	2	52.67	N
G.LSIGDVAK.K	Y	47.5	801.4596	1.2	401.7375	2	35.08	N
K.GVVKAEK.S	Y	47.09	729.4385	1.5	365.7271	2	8.88	N
V.NFSEFSKK.C	Y	46.01	985.4869	-0.2	493.7506	2	20.07	N
K.KLGEMWN.N	Y	45.85	876.4164	0.8	439.2158	2	38.31	N
T.YIPPKGETKK.K	Y	45.66	1159.66	0.7	387.5609	3	10.06	N
K.GKPDAAK.K	Y	45.29	685.3759	0.9	343.6955	2	2.9	N

K.M(+15.99)SSYAFFVQ.T	Y	45.27	1094.474	0.6	548.2447	2	63.56	Y
K.C(+57.02)SERWKTM(+15.99)SAK.E	Y	44.68	1398.638	1	700.3271	2	11.15	Y
K.RPPSAFFLFC(+57.02)S.E	Y	44.48	1327.638	1.8	664.8276	2	71.2	Y
I.KGEHPGLSIGDVAK.K	Y	43.87	1406.752	0.5	469.9247	3	28.56	N
K.KKEEEEDEEDEEDE.E	Y	43.62	1780.665	0.3	891.3398	2	11.04	N
L.SIGDVAK.K	Y	43.23	688.3755	1.1	345.1954	2	34.86	N
K.FEDM(+15.99)AK.A	Y	43.06	755.316	-1.3	378.6648	2	10.9	Y
K.HPDASVNFSEFSKK.C	Y	43	1591.763	-5	796.8848	2	28.12	N
K.GKPDAAKK.G	Y	42.98	813.4708	1.5	407.7433	2	2.76	N
K.RPPSAFFLF.C	Y	42.8	1080.576	1.1	541.2957	2	74.64	N
K.HPDASVNFSEF.S	Y	42.58	1248.541	0.5	625.2781	2	56.76	N
K.YEKDIAAY.R	Y	42.57	971.46	0.4	486.7375	2	27.12	N
H.PGLSIGDVAKK.L	Y	41.8	1083.629	2	542.8228	2	26.14	N
M.SSYAFFVQTC(+57.02)REEHK.K	Y	41.72	1887.857	1.6	630.2941	3	51.39	Y
K.KLGEMWNNTAAD.D	Y	41.69	1348.608	0.3	675.3115	2	42.18	N
F.FVQTC(+57.02)REEHK.K	Y	41.62	1332.624	-0.6	445.2151	3	10.19	Y
K.KLGEM(+15.99)WNNTAADD.K	Y	41.3	1479.63	0.7	740.8228	2	31.43	Y
K.C(+57.02)SERWKTMSAK.E	Y	40.95	1382.643	0.6	692.3294	2	17.24	Y
K.MSSYAFFVQT.C	Y	40.95	1179.527	0	590.7708	2	67.46	N
K.KHPDASVN.F	Y	40.53	866.4246	1.3	434.2201	2	9.06	N
K.GKFEDM.A	Y	40.13	725.3054	-1.2	726.3118	1	20.41	N
W.NNTAADDKQPYEK.K	Y	39.83	1492.679	-0.2	747.3467	2	11.22	N
R.AKGKPDAAK.K	Y	39.76	884.5079	5.2	443.2635	2	1.18	N
K.GKFEDM(+15.99)AK.A	Y	39.57	940.4324	-1.7	471.2227	2	11	Y
Y.IPPKGETKK.K	Y	38.98	996.5967	-0.5	333.206	3	10.9	N
K.LGEMWNNTAADD.K	Y	38.78	1335.54	1.3	668.7782	2	49.01	N
R.EM(+15.99)K(+42.01)TYIPPKGETK.K	Y	38.1	1578.796	11	527.2785	3	11.13	Y
K.LGEMWNNTAAD.D	Y	37.82	1220.513	2	611.2651	2	48.2	N
K.GEHPGLSIGDVAKKL.G	Y	36.98	1519.836	1	380.9666	4	46.76	N
K.KKEEEEDEEDEED.E	Y	36.83	1651.622	1.1	551.5485	3	10.09	N
I.KGEHPGLSIGDVAKK.L	Y	36.38	1534.847	-0.8	384.7186	4	25.69	N
K.FKDPNA.P	Y	36.08	690.3337	0.6	346.1743	2	12.79	N
R.EEHKKK.H	Y	35.32	797.4395	-0.5	399.7268	2	0.57	N
N.FSEFSK.K	Y	35.12	743.349	2.9	372.6828	2	22.94	N
K.KLGEMWNNTA.A	Y	35.04	1162.544	-0.1	582.2792	2	41.4	N
R.WKTM(+15.99)SAK.E	Y	35.02	866.432	0.6	434.2235	2	10.06	Y
K.KHPDASVNFSE.F	Y	34.97	1229.568	-0.1	615.791	2	23.8	N
K.RPPSAFF.L	Y	34.53	820.4231	1.2	411.2193	2	45.75	N
K.TYIPPK(+42.01)GETK.K	Y	34.34	1174.623	7.1	588.3231	2	18.74	Y
K.KLGEMWNNTAADDKQPY.E	Y	34.19	1979.905	0.5	990.9601	2	48.58	N
K.KC(+57.02)SER.W	Y	34.13	678.3119	1	340.1635	2	0.68	Y
K.RPPSAFFL.F	Y	33.71	933.5072	0.8	467.7613	2	64.65	N
Y.EKDIAAYR.A	Y	33.53	964.4977	1	483.2566	2	16.16	N

R.GK(+42.01)M(+15.99)SSYAFFVQTC(+57.02)R.E	Y	32.91	1738.781	3.6	580.6029	3	46.57	Y
K.IKGEHPGLS(+79.97)IGDVAK.K	Y	32.74	1599.802	-5	800.9043	2	46.27	Y
D.IAAYR.A	Y	31.66	592.3333	0.8	593.341	1	12.08	N
K.M(+15.99)SSYAFFVQTC(+57.02)REEH.K	Y	31.31	1906.798	2.7	954.4088	2	51.86	Y
K.HPDASVNFSEFSK(+42.01).K	Y	31.16	1505.679	1.6	753.8478	2	52.93	Y
K.KHPDASVNFS.E	Y	30.58	1100.525	1.8	551.2708	2	21.88	N
K.KKEEEEDEEDEE.D	Y	30.29	1536.595	0	513.2056	3	10	N
I.PPKGETK.K	Y	29.94	755.4177	0.6	378.7163	2	26.83	N
K.LGEMWNNTAADDKQPYE.K	Y	28.68	1980.852	0.8	991.4342	2	51.31	N
F.KDPNAPK.R	Y	28.46	768.413	0.9	385.2141	2	11.84	N
K.K(+42.01)LGEM(+15.99)WNNT(+79.97)AADDKQPYEK.K	Y	28.39	2375.014	3.2	1188.518	2	43.27	Y
S.VNFSEFSKK.C	Y	28.39	1084.555	0.1	543.285	2	27.87	N
K.IKGEHPGL.S	Y	28.35	849.4708	-0.1	425.7426	2	15.67	N
Y.IPPKGETK.K	Y	28.01	868.5018	0.6	435.2584	2	15.74	N
K.LKEKYEK.D	Y	27.84	936.528	-0.6	313.1831	3	9.33	N
K.EKYEK.D	Y	27.66	695.349	1.3	348.6822	2	4.89	N
K.KLGEM(+15.99)WNNTAAD.D	Y	27.57	1364.603	-0.4	683.3085	2	29.35	Y
K.MSSYAFFVQ.T	Y	27.13	1078.479	0.9	540.2474	2	67.22	N
R.EEHKK.K	Y	26.9	669.3445	0.6	335.6797	2	0.75	N
P.KIK(+42.01)GEHPGLS(+79.97)IGDVAK.K	Y	26.66	1769.908	-15.1	885.9477	2	34.49	Y
P.PKGETK.K	Y	26.32	658.365	-0.3	330.1897	2	10.82	N
K.IKGEHPGLSIGDVA.K	Y	26.27	1391.741	1.8	696.8789	2	41.02	N
G.KPDAAK.K	Y	26.07	628.3544	1.5	315.1849	2	1.31	N
K.KLGEMWNNTAADDK(+42.01)QPYEK.K	Y	25.95	2279.053	-1.6	570.7695	4	45.48	Y
S.IGDVAKK.L	Y	25.87	729.4385	0.2	365.7266	2	26.08	N
K.ARYER.E	Y	25.73	693.3558	0.9	347.6855	2	8.66	N
K.LGEM(+15.99)WNNTAADDK(+42.01)QPYEK.K	Y	25.19	2166.953	1.2	723.3257	3	30.72	Y
K.PDAAKK.G	Y	25	628.3544	1.5	315.1849	2	2.78	N
R.EMKTYIPPK.G	Y	23.83	1105.584	5.6	553.8024	2	22.91	N
K.GKPDAAKKG.V	Y	23.61	870.4922	3.3	436.2548	2	1.04	N
R.YEREM(+15.99)K.T	Y	23.36	870.3905	1.2	436.203	2	6.54	Y
F.SEFSKK.C	Y	23.17	724.3755	1.8	363.1956	2	25.24	N
I.GDVAKK.L	Y	22.88	616.3544	-1.7	309.1839	2	26.09	N
E.KDIAAYR.A	Y	21.95	835.4551	0.4	418.735	2	13.84	N
T.YIPPKGETK.K	Y	21.34	1031.565	0.8	516.7902	2	11.01	N
G.KFEDM(+15.99)AK.A	Y	21.28	883.4109	-0.1	442.7127	2	10.15	Y
A.FFVQTC(+57.02)REEHKK.K	Y	21.05	1607.788	-1.2	402.9537	4	42.03	Y
F.C(+57.02)SEYRPK.I	Y	20.54	938.428	1	470.2217	2	19.22	Y
K.DPNAPKRPPSAFFLFC(+57.02)SEYRPK.I	Y	20.5	2623.301	2.2	875.4427	3	64.38	Y
S.SYAFFVQTC(+57.02)REEHKK.K	Y	20.46	1928.92	0.5	643.981	3	42.02	Y
K.GKFEDMAKAD.K	Y	19.87	1110.502	4.2	556.2603	2	22.89	N
K.HPDASVNFSEFS.K	Y	19.58	1335.573	1.1	668.7946	2	53.28	N
K.IKGEHPGLSIG.D	Y	18.68	1106.608	-1.9	554.3104	2	26.38	N

V.QTC(+57.02)REEHKK.K	Y	18.62	1214.583	3	608.3004	2	35.02	Y
K.RPPSAFFLFC(+57.02)SEYRPKIKG.E	Y	17.85	2299.194	-1.7	767.4039	3	64.34	Y
R.EM(+15.99)K(+42.01)TYIPPK.G	Y	17.5	1163.59	2	582.8032	2	13.28	Y
K.QPYEKK.A	Y	17.21	791.4177	1.3	396.7166	2	6.99	N
F.VQTC(+57.02)REEHKK.K	Y	16.95	1313.651	0.9	438.8913	3	42.49	Y
E.K(+42.01)YEK(+42.01)DIAAYR.A	Y	15.97	1339.677	-10.4	670.8389	2	23.48	Y
A.KGKPDAAK.K	Y	15.71	813.4708	-0.4	407.7425	2	9.86	N
E.KYEKDIAAYR.A	Y	15.52	1255.656	1	419.5597	3	18.81	N
A.FFVQTC(+57.02)REEHK.K	Y	15.29	1479.693	-1.8	740.8524	2	50.89	Y
K.RPPSAFFLFC(+57.02)SEYR.P	Y	14.81	1775.845	1.2	888.931	2	69.11	Y
F.VQTC(+57.02)REEHK.K	Y	14.06	1185.556	2	593.7864	2	51.36	Y
A.ADDKQPYEK.K	Y	13.01	1092.509	-1.1	547.261	2	11.15	N
K.DIAAYRAK.G	Y	12.86	906.4922	0.7	454.2537	2	16.16	N
E.KGKFEDMAK.A	Y	12.05	1052.533	0.2	527.2736	2	14.53	N
A.ADDKQPYEKK.A	Y	11.93	1220.604	0.4	611.3093	2	32.35	N
S.EYRPK.I	Y	11.91	691.3653	-14.1	346.685	2	23.42	N
D.PKKPR.G	Y	11.61	624.4071	1.4	313.2112	2	2.79	N
C.SEYRPK.I	Y	11.48	778.3973	-0.5	390.2057	2	19.11	N
K.TYIPPKGET.K	Y	11.05	1004.518	-0.9	503.2657	2	12.93	N
F.EDMAKADK.A	Y	11.02	906.4116	17.1	454.2208	2	31.22	N
K.TY(+79.97)IPPK.G	Y	10.79	797.3724	-7.3	798.3738	1	57.21	Y
K.LGEM(+15.99)WNNTAADDKQPY.E	Y	10.42	1867.805	0.5	934.91	2	43.98	Y
K.T(+79.97)YIPPKGETK.K	Y	10.15	1212.579	9.6	607.3027	2	16.13	Y
K.KLGEM(+15.99)WNNTAADDKQPY.E	Y	10.09	1995.9	0	998.9571	2	46.53	Y
M.GKGDPK.K	Y	9.83	600.3231	2.5	301.1696	2	0.65	N
Q.TC(+57.02)REEHKK.K	Y	9.44	1086.524	-0.6	544.269	2	9.34	Y
I.KGEHPGLSIGDVAK(+42.01)K.L	Y	9.4	1576.857	0.7	526.6267	3	31.72	Y
K.EKGK(+42.01)FEDMAK.A	Y	7.74	1223.586	-1.9	612.7989	2	11.08	Y
S.VNFSEFSKK(+42.01)C(+57.02)SER.W	Y	7.35	1658.772	-0.5	830.3929	2	54.11	Y
E.M(+15.99)WNNTAADDKQPYEK.K	Y	7.35	1825.794	2.5	913.9066	2	24.58	Y
K.GKPDAAKKGVVK.A	Y	7.34	1196.724	0.1	300.1883	4	9.28	N
K.LGEM(+15.99)WNNTAADDK(+42.01)QPYEKK.A	Y	7.09	2295.048	8.1	574.7738	4	35.82	Y
K.KLGEM(+15.99)WNNTAADDKQ.P	Y	6.4	1735.784	19.2	868.9157	2	25.13	Y
K.KKEEEEDEEDEEDEEEEEDEEDEDEEEDDDD.E	Y	5.95	3876.279	1.8	1293.103	3	28.11	N

2.7 Raw LC-MS/MS data for PTM profiling of HMGB1 subform B

Peptide	Unique	-10lgP	Mass	ppm	m/z	Z	RT	PTM
K.RPPSAFFLFC(+57.02)SEYRPK.I	Y	124.49	2000.993	4.5	501.2578	4	75.22	Y
K.HPDASVNFSEFSKKC(+57.02)SER.W	Y	121.11	2123.97	0.5	531.9999	4	33.43	Y
K.LGEMWNNTAADDKQPYEK.K	Y	112.23	2108.947	0.5	703.9901	3	47.43	N
K.KLGEMWNNTAADDKQPYEK.K	Y	110.25	2237.042	0	560.2678	4	42.87	N

K.M(+15.99)SSYAFFVQTC(+57.02)REEHK.K	Y	110.17	2034.893	0.6	509.7307	4	42.91	Y
K.KLGEM(+15.99)WNNTAADDKQPYEK.K	Y	108.49	2253.037	2.8	564.2681	4	39.91	Y
N.NTAADDKQPYEKK.A	Y	105.65	1506.731	-8.3	754.3667	2	10	N
K.LGEM(+15.99)WNNTAADDKQPYEK.K	Y	101.25	2124.942	-0.3	1063.478	2	40.34	Y
R.GK(+42.01)M(+15.99)SSYAFFVQTC(+57.02)R.E	Y	101.09	1738.781	-1.2	870.3965	2	58.21	Y
K.M(+15.99)SSYAFFVQTC(+57.02)REEHKK.K	Y	100.64	2162.988	-1	1082.5	2	33.98	Y
K.HPDASVNFSEFSK.K	Y	100	1463.668	1.6	732.8425	2	48.13	N
P.PSAFFLFC(+57.02)SEYRPK.I	Y	99.39	1747.839	2.6	583.6218	3	68.31	Y
R.GKM(+15.99)SSYAFFVQTC(+57.02)R.E	Y	97.65	1696.77	-0.1	849.3922	2	49.51	Y
R.GKMSSYAFFVQTC(+57.02)R.E	Y	96.97	1680.775	2.5	841.397	2	55.42	Y
S.AFFLFC(+57.02)SEYRPK.I	Y	96.12	1563.754	0.4	782.8848	2	64.8	Y
K.MSSYAFFVQTC(+57.02)R.E	Y	94.86	1495.659	1.1	748.8375	2	67.5	Y
K.M(+15.99)SSYAFFVQTC(+57.02)R.E	Y	94.15	1511.654	0.2	756.8343	2	65.57	Y
K.GEHPGLSIGDVAKK.L	Y	93.09	1406.752	0.3	469.9246	3	25.45	N
K.KLGEMWNNTAADDK.Q	Y	92.78	1591.73	-1.7	796.8709	2	33.66	N
K.MSSYAFFVQTC(+57.02)REEHKK.K	Y	92.57	2146.993	-0.5	1074.503	2	42.08	Y
K.IKGEHPGLSIGDVAKK.L	Y	92.31	1647.931	1.3	412.9905	4	22.37	N
K.MSSYAFFVQTC(+57.02)REEHK.K	Y	92.25	2018.898	1.5	673.9742	3	51.98	Y
K.RPPSAFFLFC(+57.02)SEYR.P	Y	92.22	1775.845	1.1	592.9564	3	68.97	Y
K.KHPDASVNFSEFSK.K	Y	92.21	1591.763	1.5	398.9486	4	41.43	N
K.YEKDIAAYRAK.G	Y	89.55	1326.693	2	664.3552	2	19.41	N
K.KHPDASVNFSEFSKK.C	Y	89.12	1719.858	2.3	430.9727	4	26.77	N
N.NTAADDKQPYEK.K	Y	88.1	1378.637	-1.5	690.3245	2	10.99	N
R.GK(+42.01)MSSYAFFVQTC(+57.02)R.E	Y	87.32	1722.786	1.3	862.4013	2	64.6	Y
R.GKM(+15.99)SSYAFFVQTC(+57.02)REEHK.K	Y	87.26	2220.009	3.3	556.0114	4	33.85	Y
K.TYIPPKGETK.K	Y	87.23	1132.613	-3.3	567.3118	2	10.86	N
K.HPDASVNFSEFSKK.C	Y	86.92	1591.763	0	531.595	3	32.75	N
L.GEMWNNTAADDKQPYEK.K	Y	86.65	1995.863	-1.1	998.9378	2	33.46	N
K.LGEM(+15.99)WNNTAADDKQPYEKK.A	Y	85.66	2253.037	0	752.0197	3	25.77	Y
S.SYAFFVQTC(+57.02)R.E	Y	85.31	1277.586	3.3	639.8026	2	55.92	Y
K.IKGEHPGLSIGDVAK.K	Y	85.12	1519.836	2	507.6202	3	47.83	N
K.IK(+42.01)GEHPGLSIGDVAK.K	Y	84.75	1561.846	1.3	781.9315	2	41.78	Y
G.EMWNNTAADDKQPYEK.K	Y	84.22	1938.842	1.4	970.4296	2	33.28	N
K.FKDPNAPKRPPSAFFLFC(+57.02)SEYRPK.I	Y	83.94	2898.464	-1	725.6225	4	62.25	Y
K.GEHPGLSIGDVAK.K	Y	83.61	1278.657	-1.5	640.3347	2	26.29	N
R.PPSAFFLFC(+57.02)SEYRPK.I	Y	83.41	1844.892	1.5	615.9721	3	69.23	Y
P.SAFFLFC(+57.02)SEYRPK.I	Y	83.35	1650.786	-3.9	826.3972	2	64.76	Y
G.EMWNNTAADDKQPYEKK.A	Y	81.89	2066.937	-0.6	689.9858	3	26.1	N
P.KRPPSAFFLFC(+57.02)SEYRPK.I	Y	81.77	2129.088	2.1	710.7047	3	61.35	Y
H.PDASVNFSEFSKK.C	Y	80.58	1454.704	-0.3	485.9085	3	42.64	N
K.KLGEM(+15.99)WNNTAADDK.Q	Y	79.52	1607.725	-0.5	804.8693	2	25.57	Y
K.KHPDASVNFSEFSK(+42.01).K	Y	79.02	1633.774	1	545.599	3	47.01	Y
H.PDASVNFSEFSK.K	Y	78.39	1326.609	1	664.3125	2	51.47	N

K.LGEMWNNTAADDK.Q	Y	78.32	1463.635	0.2	732.825	2	40.14	N
E.MWNNTAADDKQPYEK.K	Y	77.99	1809.799	-0.5	905.9064	2	26.13	N
R.EMKTYIPPKGETK.K	Y	77.57	1520.791	0.2	761.4028	2	19.3	N
M.SSYAFFVQTC(+57.02)R.E	Y	77.06	1364.618	4.2	683.3193	2	55.88	Y
K.KPRGKMSSYAFFVQTC(+57.02)R.E	Y	76.98	2062.024	0.9	688.3492	3	41.78	Y
G.EHPGLSIGDVAK.K	Y	76.56	1221.635	-1.4	408.2184	3	33.99	N
K.YEKDIAAYR.A	Y	76.51	1127.561	2.5	564.7892	2	23.08	N
W.NNTAADDKQPYEKK.A	Y	76.29	1620.774	-5.7	811.3898	2	10.31	N
Y.AFFVQTC(+57.02)R.E	Y	76.27	1027.491	0.7	514.7531	2	37.65	Y
K.EKYEKDIAAYR.A	Y	74.54	1384.699	-1.5	693.3555	2	24.1	N
K.YEK(+42.01)DIAAYR.A	Y	74.53	1169.572	-0.3	585.7929	2	38.36	Y
K.LGEM(+15.99)WNNTAADDK.Q	Y	73.99	1479.63	2.4	740.824	2	32.81	Y
G.KMSSYAFFVQTC(+57.02)R.E	Y	73.44	1623.754	2.6	542.2599	3	55.23	Y
K.TYIPPKGETKK.K	Y	72.73	1260.708	-0.9	316.1839	4	10.82	N
K.TYIPPK(+42.01)GETK.K	Y	71.39	1174.623	-0.1	588.3188	2	24.79	Y
S.YAFFVQTC(+57.02)R.E	Y	71.19	1190.554	2.3	596.2857	2	54.18	Y
G.EHPGLSIGDVAKK.L	Y	70.36	1349.73	2.3	675.874	2	26.22	N
F.LFC(+57.02)SEYRPK.I	Y	70.31	1198.58	0.6	600.2979	2	26.11	Y
K.DIAAYR.A	Y	70.23	707.3602	-0.1	354.6873	2	25.82	N
N.TAADDKQPYEKK.A	Y	70.22	1392.689	-2.6	697.3497	2	9.91	N
K.RPPSAFFLFC(+57.02).S	Y	69.06	1240.606	1	621.311	2	72.11	Y
L.GEM(+15.99)WNNTAADDKQPYEK.K	Y	68.95	2011.858	-0.6	671.6262	3	25.95	Y
Y.AFFVQTC(+57.02)REEHKK.K	Y	68.82	1678.825	-1	840.4189	2	19.64	Y
M.WNNTAADDKQPYEK.K	Y	68.71	1678.759	0.9	840.3873	2	21.89	N
G.EM(+15.99)WNNTAADDKQPYEK.K	Y	68.43	1954.837	0.2	978.4258	2	25.75	Y
K.FEDMAKADKAR.Y	Y	68.36	1280.618	-3.1	641.3144	2	15.37	N
K.RPPSAFFLFC(+57.02)S.E	Y	68.06	1327.638	1.6	664.8275	2	71.4	Y
K.KKHPDASVNFSEFSK.K	Y	67.58	1719.858	-2.5	860.9341	2	24.71	N
F.C(+57.02)SEYRPK.I	Y	67.56	938.428	-1.9	470.2203	2	9.96	Y
W.NNTAADDKQPYEK.K	Y	67.5	1492.679	-1.3	747.3459	2	11.24	N
K.K(+42.01)LGEM(+15.99)WNNTAADDKQPYEK.K	Y	67.41	2295.048	-0.1	766.0231	3	40.47	Y
K.IKGEHPGLSIGD.V	Y	67.1	1221.635	-0.7	611.8245	2	26.14	N
G.KM(+15.99)SSYAFFVQTC(+57.02)R.E	Y	66.77	1639.749	0.7	547.5905	3	49.25	Y
A.FFLFC(+57.02)SEYRPK.I	Y	66.67	1492.717	-2	747.3644	2	64.78	Y
P.DASVNFSEFSKK.C	Y	66.64	1357.651	0.1	679.833	2	42.04	N
K.GKFEDMAKADK.A	Y	66.04	1238.596	-1.2	620.3047	2	19.29	N
R.AK(+42.01)GKPDAAK.K	Y	65.92	926.5185	-0.9	309.8465	3	9.16	Y
Y.AFFVQTC(+57.02)REEHK.K	Y	65.54	1550.73	0	517.9172	3	24.62	Y
A.SVNFSEFSKK.C	Y	65.41	1171.587	-0.1	586.8008	2	33.73	N
K.FK(+42.01)DPNAPK.R	Y	65.35	957.4919	-0.1	479.7532	2	19.46	Y
F.FLFC(+57.02)SEYRPK.I	Y	64.93	1345.649	0.8	449.5572	3	49.11	Y
S.VNFSEFSK.K	Y	64.28	956.4603	0.4	479.2376	2	41.37	N
K.K(+42.01)HPDASVNFSEFSK.K	Y	64.14	1633.774	-0.2	545.5983	3	42.36	Y

A.FFVQTC(+57.02)R.E	Y	63.49	956.4538	3.1	479.2357	2	31.07	Y
F.FVQTC(+57.02)REEHKK.K	Y	63.17	1460.719	-1.3	731.366	2	9.26	Y
K.RPPSAFFLFC(+57.02)SE.Y	Y	62.91	1456.681	2.3	729.3494	2	70.98	Y
K.RPPSAF.F	Y	62.72	673.3547	0	337.6846	2	18.77	N
K.TYIPPK.G	Y	62.71	717.4061	-1.1	359.7099	2	26.9	N
K.C(+57.02)SERWKTMSAK.E	Y	62.35	1382.643	-0.4	692.3287	2	15.76	Y
K.FEDMAKADK.A	Y	62.35	1053.48	0.3	527.7475	2	15.69	N
P.DASVNFSEFSK.K	Y	62.13	1229.556	3	615.7873	2	52.75	N
K.KFK(+42.01)DPNAPK.R	Y	62.09	1085.587	-1.5	362.869	3	15.42	Y
K.KKHPDASVNFSEF.S	Y	60.57	1504.731	0.4	502.5844	3	39.05	N
K.FKDPNAPK.R	Y	60.29	915.4814	-0.3	458.7478	2	23.3	N
F.FVQTC(+57.02)R.E	Y	59.61	809.3854	-1.5	405.6993	2	11.65	Y
L.FC(+57.02)SEYRPK.I	Y	58.79	1085.496	0.9	543.7559	2	18.87	Y
K.FEDMAK.A	Y	58.33	739.321	0.6	370.668	2	17.07	N
K.GKFEDMAK.A	Y	58.13	924.4375	0.1	309.1531	3	17.47	N
K.KFKDPNAPK.R	Y	58.04	1043.576	-1.6	522.7946	2	11.92	N
T.AADDKQPYEK.K	Y	57.95	1163.546	1.3	582.7809	2	10.71	N
K.DIAAYRAKGKPDAAK.K	Y	57.76	1573.858	1.3	525.6271	3	15.27	N
D.ASVNFSEFSK.K	Y	55.8	1114.529	1.6	558.2729	2	46.93	N
T.AADDKQPYEKK.A	Y	54.88	1291.641	-0.6	646.8273	2	32.32	N
D.ASVNFSEFSKK.C	Y	52.86	1242.624	-0.5	622.3192	2	34.46	N
K.EKGKFEDMAK.A	Y	52.04	1181.575	-1.8	591.7937	2	12.08	N
T.YIPPKGETKK.K	Y	51.69	1159.66	-2.3	387.5597	3	10.86	N
K.LGEM(+15.99)WNNTAADD.K	Y	51.54	1351.535	-0.5	676.7744	2	37.4	Y
K.KHPDASVNFSEF.S	Y	51.28	1376.636	0	689.3253	2	49.95	N
M.WNNTAADDKQPYEKK.A	Y	51.22	1806.854	-1.4	904.4328	2	18.29	N
G.LSIGDVAK.K	Y	50.44	801.4596	1.1	401.7375	2	34.3	N
K.K(+42.01)LGEMWNNTAADDKQPYEK.K	Y	49.86	2279.053	1.4	760.6926	3	51.31	Y
K.HPDASVNFSEF.S	Y	49.34	1248.541	0.5	625.2781	2	56.39	N
K.TMSAK(+42.01)EK.G	Y	49.29	835.4109	-2.8	418.7115	2	10.99	Y
K.YEKDIAAY.R	Y	49.07	971.46	-0.3	486.7371	2	26.2	N
H.PGLSIGDVAK.K	Y	49.05	955.5338	0.5	478.7744	2	40.82	N
N.TAADDKQPYEK.K	Y	48.86	1264.594	-2.9	633.3022	2	30.63	N
K.KHPDASVNF.S	Y	48.85	1013.493	0.9	507.7542	2	22.58	N
N.FSEFSKK.C	Y	48.78	871.4439	-0.2	436.7291	2	24.41	N
K.KLGEMWNNTAAD.D	Y	48.75	1348.608	0.5	675.3117	2	42.18	N
K.KLGEMWNNTAA.D	Y	48.46	1233.581	0.8	617.7983	2	42.38	N
K.M(+15.99)SSYAFFVQ.T	Y	48.25	1094.474	0.9	548.2449	2	63.47	Y
K.KLGEMWNNTAADDKQPY.E	Y	47.93	1979.905	-2.3	990.9573	2	48.8	N
K.EKGKFEDM(+15.99)AK.A	Y	47.14	1197.57	-1.9	599.7911	2	9.88	Y
K.GKPDAAK.K	Y	45.94	685.3759	2.3	343.696	2	1.33	N
R.WKTMSAK.E	Y	45.71	850.4371	-1.3	426.2253	2	13.17	N
K.IKGEHPGLS(+79.97)IGDVAK.K	Y	45.36	1599.802	-7.6	800.9022	2	46.08	Y

K.LGEMWNNTAADDKQPY.E	Y	45.06	1851.81	1.5	926.9135	2	52.64	N
A.SVNFSEFSK.K	Y	44.6	1043.492	1.1	522.754	2	43.68	N
K.RPPSAFFLF.C	Y	44.57	1080.576	1.4	541.2958	2	74.8	N
V.NFSEFSKK.C	Y	44.48	985.4869	-0.5	493.7505	2	24.42	N
K.GKFEDM(+15.99)AK.A	Y	43.81	940.4324	-3.3	314.4837	3	10.4	Y
Y.EKDIAAYR.A	Y	43.44	964.4977	0.5	322.5067	3	15.74	N
G.EM(+15.99)WNNTAADDKQPYEKK.A	Y	43.39	2082.932	0.7	695.3183	3	22.22	Y
K.LGEMWNNTAAD.D	Y	42.99	1220.513	1.7	611.2649	2	48.27	N
K.GK(+42.01)PDAAK.K	Y	42.92	727.3864	-2.1	364.6997	2	10.05	Y
I.KGEHPGLSIGDVAK.K	Y	42.2	1406.752	-0.1	704.3831	2	29.52	N
K.GKPDAAKK.G	Y	41.9	813.4708	1	407.7431	2	2.7	N
K.GVVKAEK.S	Y	41.69	729.4385	2.3	365.7273	2	9.02	N
K.GK(+42.01)FEDMAK.A	Y	41.11	966.4481	13	484.2376	2	22.09	Y
K.K(+42.01)HPDASVNFSEFSKK.C	Y	40.58	1761.869	0.7	588.2972	3	33.88	Y
K.KLGEM(+15.99)WNNTAADD.K	Y	40.55	1479.63	0.7	740.8228	2	31.49	Y
R.GKMSSYAFFVQTC(+57.02)REEHK.K	Y	40.49	2204.014	-0.6	552.0105	4	45.91	Y
L.SIGDVAK.K	Y	40.37	688.3755	-0.5	345.1949	2	33.81	N
K.HPDASVNFSEFSK(+42.01).K	Y	40.34	1505.679	1.6	753.8478	2	52.9	Y
K.KHPDASVNFS.E	Y	40.31	1100.525	0.3	551.27	2	19.64	N
K.MSSYAFFVQ.T	Y	39	1078.479	-0.1	540.2469	2	67.24	N
M.GK(+42.01)GDPKKPR.G	Y	38.65	1023.583	-0.4	512.7983	2	9.15	Y
K.TYIPPKGETK(+42.01)K.K	Y	38.57	1302.718	-1.6	652.3654	2	17.7	Y
Y.RPK(+42.01)IKGEHPGLSIGDVAK.K	Y	38.5	1943.095	-19.8	648.6929	3	26.05	Y
K.TM(+15.99)SAK(+42.01)EK.G	Y	38.28	851.4059	0.2	426.7103	2	9.04	Y
K.FEDM(+15.99)AK.A	Y	38.17	755.316	-2.9	378.6642	2	10.56	Y
K.KLGEMWNNTA.A	Y	37.89	1162.544	-0.3	582.2791	2	41.15	N
K.KLGEMWN.N	Y	37.46	876.4164	1	439.2159	2	37.89	N
K.MSSYAFFVQT.C	Y	37.06	1179.527	1.9	590.7719	2	67.51	N
K.KLGEM(+15.99)WNNTAADDKQPY.E	Y	36.78	1995.9	-0.7	998.9564	2	37.64	Y
K.LGEM(+15.99)WNNTAADDKQPY.E	Y	36.71	1867.805	-1	934.9087	2	44.13	Y
K.C(+57.02)SERWK.T	Y	36.41	864.3912	-2	433.202	2	10.94	Y
Y.IPPKGETKK.K	Y	35.86	996.5967	-3.2	333.2051	3	10.86	N
K.MSSYAFFVQTC(+57.02)REEH.K	Y	35.14	1890.803	-1.4	631.274	3	56.35	Y
K.KLGEMWNNT.A	Y	35.06	1091.507	0.7	546.7611	2	39.42	N
K.RPPSAFFL.F	Y	34.73	933.5072	0.8	467.7612	2	64.52	N
K.RPPSAFF.L	Y	34.47	820.4231	0.9	411.2192	2	45.46	N
K.KC(+57.02)SER.W	Y	34.2	678.3119	-0.2	340.1631	2	0.74	Y
K.IKGEHPGL.S	Y	33.99	849.4708	-1.1	425.7422	2	15.22	N
F.FVQTC(+57.02)REEHK.K	Y	32.83	1332.624	-4.1	667.3167	2	10.31	Y
K.RPPSAFFLFC(+57.02)SEY.R	Y	32.49	1619.744	1.5	810.8806	2	72.74	Y
K.IKGEHPGLSIG.D	Y	32.15	1106.608	-0.7	554.3111	2	25.75	N
H.PGLSIGDVAKK.L	Y	31.69	1083.629	-0.7	362.2166	3	25.17	N
K.C(+57.02)SERWKTM(+15.99)SAK.E	Y	31.07	1398.638	-0.2	700.3264	2	10.98	Y

K.KLGEMWNN.T	Y	30.91	990.4593	-0.9	496.2365	2	36.24	N
M.SSYAFFVQTC(+57.02)REEHKK.K	Y	30.7	2015.952	-0.4	672.9911	3	41.81	Y
K.KHPDASVNFSE.F	Y	30.02	1229.568	-0.1	615.791	2	23.21	N
K.KKEEEEDEEDEED.E	Y	29.77	1651.622	-2.7	826.816	2	10.16	N
N.FSEFSK.K	Y	29.71	743.349	-8.9	744.3497	1	45.41	N
S.IGDVAKK.L	Y	28.63	729.4385	-0.2	365.7264	2	25.12	N
P.PKGETK.K	Y	28.6	658.365	-0.6	330.1896	2	10.83	N
K.LGEMWNNTAADD.K	Y	28.46	1335.54	0.5	668.7776	2	49.02	N
K.LKEKYEK.D	Y	28.13	936.528	-1.8	313.1827	3	9.32	N
G.LSIGDVAKK.L	Y	27.91	929.5546	-2	465.7836	2	24.77	N
R.EEHKKK.H	Y	27.86	797.4395	-1.7	399.7263	2	0.66	N
K.KLGEM(+15.99)WNNTAAD.D	Y	27.11	1364.603	-0.5	683.3084	2	28.96	Y
K.KLGEM(+15.99)WNN.T	Y	26.9	1006.454	-0.5	504.2341	2	24.46	Y
V.NFSEFSK.K	Y	26.76	857.3919	0.8	429.7036	2	43.17	N
K.EKYEK.D	Y	26.7	695.349	-0.1	348.6817	2	6.65	N
R.EEHKK.K	Y	26.61	669.3445	0.4	335.6797	2	3.76	N
A.ADDKQPYEKK.A	Y	26.29	1220.604	-0.8	611.3086	2	31.69	N
K.PDAAKK.G	Y	26.2	628.3544	0.5	315.1846	2	2.71	N
I.KGEHPGLSIGDVAKK.L	Y	26.07	1534.847	-0.9	384.7186	4	25.18	N
D.IAAYR.A	Y	25.85	592.3333	-0.3	593.3403	1	22.74	N
K.QPYEKK.A	Y	25.81	791.4177	-0.3	396.716	2	7.37	N
I.GDVAKK.L	Y	25.73	616.3544	0.4	309.1846	2	25.21	N
F.KDPNAPK.R	Y	25.59	768.413	-2.2	385.2129	2	11.31	N
K.ARYER.E	Y	25.37	693.3558	0.4	347.6853	2	8.8	N
F.SEFSKK.C	Y	25.13	724.3755	0.2	363.1951	2	24.48	N
E.HPGLSIGDVAK.K	Y	24.32	1092.593	0.7	547.304	2	33.77	N
E.KDIAAYR.A	Y	23.54	835.4551	0.3	418.735	2	22.99	N
D.DKQPYEK.K	Y	23.51	906.4446	-6.5	454.2266	2	36.82	N
G.KFEDM(+15.99)AK.A	Y	21.92	883.4109	-2.4	442.7117	2	10.55	Y
E.KYEKDIAAYR.A	Y	21.05	1255.656	-0.3	628.8351	2	18.58	N
R.WKTM(+15.99)SAK.E	Y	20.02	866.432	-2.5	434.2222	2	9.82	Y
K.LGEMWNNTAADDKQPYE.K	Y	18.97	1980.852	4.7	991.438	2	51.23	N
K.LGEMWNNTAADDK(+42.01)QPYEK.K	Y	18.8	2150.958	-12.4	1076.473	2	53.8	Y
K.LGEM(+15.99)WNNTAADDK(+42.01)QPYEK.K	Y	18.72	2166.953	0.6	723.3253	3	32.98	Y
K.IKGEHPGLSIGDVA.K	Y	17.28	1391.741	0.4	696.8779	2	40.78	N
Y.IPPKGETK.K	Y	16.28	868.5018	-0.2	435.2581	2	18.32	N
K.GKPDAAKKG.V	Y	15.36	870.4922	-0.1	436.2534	2	2.74	N
K.KHPDASVN.F	Y	14.46	866.4246	-1.1	434.2191	2	9.19	N
M.GKGDPK.K	Y	13.79	600.3231	0	301.1688	2	0.73	N
S.VNFSEFSKK(+42.01)C(+57.02)SER.W	Y	12.95	1658.772	-0.1	830.3933	2	54.17	Y
R.WKTM(+15.99)SAKEK.G	Y	12.36	1123.57	-1.5	562.7912	2	9.7	Y
K.LKEKYE.K	Y	12.36	808.433	0.2	405.2238	2	10.92	N
R.YEREM(+15.99)K.T	Y	11.99	870.3905	-0.1	436.2025	2	7.27	Y

F.VQTC(+57.02)REEHKK.K	Y	11.83	1313.651	-0.3	657.8326	2	41.81	Y
M.KTYIPPK.G	Y	11.72	845.501	-2.4	423.7567	2	11.04	N
M(+15.99)GKGDPK.K	Y	11.7	747.3585	5	374.6884	2	28.84	Y
R.EMKTYIPPK.G	Y	11.6	1105.584	-17.2	553.7898	2	21.78	N
S.VNFSEFSKK.C	Y	11.59	1084.555	-0.7	543.2845	2	34.93	N
S.SYAFFVQTC(+57.02)REEHK.K	Y	11.25	1800.825	0.2	601.2825	3	51.15	Y
R.WK(+42.01)TMSAK.E	Y	11.19	892.4477	13.1	447.237	2	15.78	Y
I.PPKGETKK.K	Y	10.96	883.5127	-0.6	442.7634	2	10.82	N
A.FFVQTC(+57.02)REEHKK.K	Y	10.74	1607.788	3.9	402.9558	4	41.85	Y
K.YEKDIAAYRA.K	Y	10.68	1198.598	0.5	400.5402	3	23.84	N
K.MSSYAFF.V	Y	10.25	851.3524	1.8	426.6842	2	68.21	N
D.PKKPR.G	Y	10.19	624.4071	0.4	313.2109	2	2.74	N
K.AAKLK(+42.01)EK.Y	Y	9.98	828.5068	6.8	829.5198	1	60.66	Y
K.KLGEMWNNTAADDKQ.P	Y	9.94	1719.789	-0.4	860.9012	2	33.7	N
R.EMKT(+79.97)YIPPK.G	Y	9.53	1185.55	6.2	593.7861	2	51.38	Y
K.GDPK(+42.01)K(+42.01)PR.G	Y	9.32	880.4766	-11.7	441.2404	2	58.78	Y
P.RGKM(+15.99)SSYAFFVQTC(+57.02)R.E	Y	8.92	1852.871	-16.5	927.4276	2	59.45	Y
K.LGEM(+15.99)WNNTAAD.D	Y	8.52	1236.508	11.5	619.2684	2	48.36	Y
K.GKPDAAKKGVVK.A	Y	8.4	1196.724	-0.9	399.9149	3	9.15	N
P.KIK(+42.01)GEHPGLS(+79.97)IGDVAK.K	Y	7.83	1769.908	-15.8	590.9671	3	56.97	Y
I.KGEHPGLSIGDVAK(+42.01)K.L	Y	7.25	1576.857	-1.6	526.6255	3	31.45	Y
K.PDAAKKGVVK.A	Y	7.08	1011.608	-0.2	506.8109	2	9.14	N
R.EMK(+42.01)TYIPPK.G	Y	6.69	1147.595	-12.9	383.5339	3	9.61	Y
K.TMSAKEKG.K	Y	6.59	850.4218	0.4	426.2183	2	5.63	N
E.KGKFEDMAK.A	Y	6.49	1052.533	-0.7	527.2731	2	14.17	N
T.YIPPKGETK.K	Y	6.2	1031.565	11.5	516.7957	2	25.19	N
A.ADDKQPYEK.K	Y	5.61	1092.509	2.1	547.2628	2	10.8	N
K.DPNAPKR.P	Y	5.34	796.4191	-15.1	399.2108	2	11.1	N

2.8 Raw LC-MS/MS data for PTM profiling of HMGB1 subform C

Peptide	Unique	-10lgP	Mass	ppm	m/z	Z	RT	PTM
K.LGEMWNNTAADDKQPYEK.K	Y	130.47	2108.9473	-0.7	1055.4802	2	44.19	N
K.RPPSAFFLFC(+57.02)SEYRPK.I	Y	129.88	2000.993	0.2	501.2556	4	70.02	Y
K.KLGEMWNNTAADDKQPYEK.K	Y	112.31	2237.0422	1.5	560.2687	4	41.43	N
R.GK(+42.01)M(+15.99)SSYAFFVQTC(+57.02)R.E	Y	109.96	1738.7806	-0.4	870.3973	2	61.27	Y
K.LGEM(+15.99)WNNTAADDKQPYEK.K	Y	108.82	2124.9421	0.3	709.3215	3	41.94	Y
K.KLGEM(+15.99)WNNTAADDKQPYEK.K	Y	105.72	2253.0371	0.6	564.2669	4	33.51	Y
K.M(+15.99)SSYAFFVQTC(+57.02)R.E	Y	105.28	1511.6537	0.1	756.8342	2	75.39	Y
K.MSSYAFFVQTC(+57.02)R.E	Y	99.33	1495.6588	-0.7	748.8361	2	72.43	Y
K.KHPDASVNFSEFSK.K	Y	99.08	1591.7631	1.4	531.5957	3	41.5	N
R.GKMSSYAFFVQTC(+57.02)R.E	Y	97.72	1680.7753	2	841.3966	2	55.71	Y

K.HPDASVNFSEFSK.K	Y	95.16	1463.6681	3.4	488.8983	3	47.21	N
K.K(+42.01)LGEMWNNTAADDKQPYEK.K	Y	95.03	2279.0527	-1.8	760.6902	3	48.98	Y
K.RPPSAFFLFC(+57.02)SEYR.P	Y	94.91	1775.8453	2.4	592.9571	3	69.14	Y
K.LGEMWNNTAADDKQPYEKK.A	Y	94.07	2237.0422	0.3	746.6882	3	39.4	N
K.M(+15.99)SSYAFFVQTC(+57.02)REEHKK.K	Y	92.96	2162.9878	1.4	722.0042	3	41.65	Y
K.KKHPDASVNFSEFSK.K	Y	90.12	1719.8579	0.6	574.2936	3	37.32	N
K.RPPSAFFLFC(+57.02)S.E	Y	88.68	1327.6383	2.3	664.8279	2	71.16	Y
K.KHPDASVNFSEFSKK.C	Y	88.54	1719.8579	1.1	430.9722	4	37.1	N
R.GK(+42.01)MSSYAFFVQTC(+57.02)R.E	Y	88.39	1722.7858	1.5	862.4014	2	65.02	Y
S.SYAFFVQTC(+57.02)R.E	Y	87.53	1277.5863	-0.5	639.8001	2	56.22	Y
K.KLGEMWNNTAADDK.Q	Y	87.19	1591.73	0.5	531.5842	3	38.3	N
M.SSYAFFVQTC(+57.02)R.E	Y	86.07	1364.6183	1.7	683.3176	2	56.13	Y
R.GKM(+15.99)SSYAFFVQTC(+57.02)R.E	Y	84.71	1696.7701	1.1	849.3933	2	50.73	Y
K.TYIPPKGETK.K	Y	83.91	1132.6128	2.7	567.3152	2	29.35	N
K.LGEMWNNTAADDK.Q	Y	83.49	1463.6351	8.6	732.8311	2	42.19	N
K.GEHPGLSIGDVAKK.L	Y	82.33	1406.7517	2.2	352.696	4	33.13	N
K.IKGEHPGLSIGDVAK.K	Y	81	1519.8358	2.1	507.6203	3	37.4	N
H.PDASVNFSEFSK.K	Y	80.34	1326.6091	1	664.3125	2	51.56	N
K.MSSYAFFVQTC(+57.02)REEHK.K	Y	80.07	2018.8978	2.7	673.975	3	52.58	Y
G.KMSSYAFFVQTC(+57.02)R.E	Y	78.54	1623.7538	1.9	542.2596	3	55.37	Y
K.TYIPPK(+42.01)GETK.K	Y	76.61	1174.6233	2.2	588.3202	2	30.18	Y
K.LGEM(+15.99)WNNTAADDK.Q	Y	75.36	1479.63	-0.2	740.8221	2	38.15	Y
S.YAFFVQTC(+57.02)R.E	Y	74.46	1190.5542	1.1	596.285	2	54.49	Y
K.KHPDAS(+79.97)VNFSEFSK.K	Y	74	1671.7294	-1.2	558.2497	3	44.21	Y
K.MSSYAFFVQTC(+57.02)REEHKK.K	Y	73.28	2146.9927	1.5	716.6726	3	48.4	Y
K.M(+15.99)SSYAFFVQTC(+57.02)REEHK.K	Y	72.9	2034.8927	2.1	679.3062	3	47.55	Y
R.AK(+42.01)GKPDAAK.K	Y	72.46	926.5185	-1.4	309.8463	3	10.01	Y
G.KM(+15.99)SSYAFFVQTC(+57.02)R.E	Y	70.26	1639.7487	1.4	547.5909	3	50.52	Y
K.GEHPGLSIGDVAK.K	Y	70.16	1278.6567	1.4	427.2268	3	38.59	N
Y.AFFVQTC(+57.02)R.E	Y	69.06	1027.491	1.4	514.7535	2	42.04	Y
K.KLGEM(+15.99)WNNTAADDK.Q	Y	68.54	1607.725	0.5	804.8701	2	29.68	Y
G.EHPGLSIGDVAK.K	Y	67.33	1221.6353	0.6	408.2193	3	38.99	N
S.VNFSEFSK.K	Y	67.14	956.4603	1	479.2379	2	43.79	N
F.C(+57.02)SEYRPK.I	Y	66.3	938.428	2	470.2222	2	11.92	Y
A.FFVQTC(+57.02)R.E	Y	66.25	956.4538	0.6	479.2344	2	36.26	Y
K.RPPSAFFLFC(+57.02)SEY.R	Y	66.11	1619.7441	-1.2	810.8784	2	72.6	Y
K.MSS(+79.97)YAFFVQTC(+57.02)R.E	Y	65.89	1575.6251	0.8	788.8204	2	62.3	Y
F.FLFC(+57.02)SEYRPK.I	Y	65.57	1345.6488	1.4	673.8326	2	50.57	Y
K.YEKDIAAYR.A	Y	64.57	1127.561	1.4	564.7886	2	27.6	N
K.IKGEHPGLSIGDVAKK.L	Y	64.49	1647.9308	2	550.3186	3	32.37	N
T.AADDKQPYEK.K	Y	64.31	1163.5458	0.9	582.7807	2	12.5	N
F.LFC(+57.02)SEYRPK.I	Y	64.19	1198.5804	1.6	600.2985	2	32.21	Y
K.HPDAS(+79.97)VNFSEFSK.K	Y	64.11	1543.6344	2.2	772.8262	2	49.52	Y

K.YEK(+42.01)DIAAYR.A	Y	63.88	1169.5717	-1	585.7925	2	40.8	Y
K.KLGEMWNNT.A	Y	63.6	1091.507	1.5	546.7615	2	43.07	N
K.DIAAYR.A	Y	63.16	707.3602	0.3	354.6875	2	23.83	N
K.RPPSAFFLFC(+57.02).S	Y	62.4	1240.6062	-0.2	621.3102	2	71.99	Y
P.DASVNFSEFSKK.C	Y	62.18	1357.6514	-0.5	679.8326	2	44.29	N
D.ASVNFSEFSKK.C	Y	61.7	1242.6244	0.6	622.3198	2	40.56	N
N.TAADDKQPYEK.K	Y	61.08	1264.5935	2.2	633.3054	2	13.41	N
K.RPPSAFFLFC(+57.02)SE.Y	Y	60.9	1456.6809	-1.2	729.3469	2	70.98	Y
K.TMS(+79.97)AKEK.G	Y	60.34	873.3667	0.6	437.6909	2	9.22	Y
K.TYIPPK.G	Y	60.26	717.4061	0.8	359.7106	2	19.79	N
K.FKDPNAPK.R	Y	58.23	915.4814	1.3	306.1681	3	15.54	N
P.DASVNFSEFSK.K	Y	57.7	1229.5564	2.7	615.7871	2	51.67	N
K.TYIPPKGETKK.K	Y	56.85	1260.7078	0.7	631.3616	2	14.35	N
D.ASVNFSEFSK.K	Y	56.83	1114.5294	3.1	558.2737	2	47.86	N
P.KIKGEHPGLSIGDVAK.K	Y	56.13	1647.9308	1.1	412.9904	4	33.62	N
F.FVQTC(+57.02)R.E	Y	54.66	809.3854	1.7	405.7006	2	14.87	Y
K.FEDMAK.A	Y	54.19	739.321	0.7	370.668	2	20.09	N
K.KFKDPNAPK.R	Y	54.17	1043.5763	0	522.7954	2	14.34	N
K.RPPSAF.F	Y	53.87	673.3547	1.8	337.6852	2	23.25	N
G.LSIGDVAK.K	Y	53.22	801.4596	2.4	401.738	2	37.9	N
N.NTAADDKQPYEK.K	Y	52.76	1378.6365	1.3	690.3264	2	13.72	N
K.LGEM(+15.99)WNNTAADDKQPY.E	Y	52.44	1867.8047	-0.7	934.9089	2	44.91	Y
K.EKGKFEDMAK.A	Y	52.4	1181.575	-0.3	591.7946	2	20.64	N
K.GKFEDMAK.A	Y	51.95	924.4375	1.3	309.1535	3	21.61	N
L.FC(+57.02)SEYRPK.I	Y	50.75	1085.4963	0.7	543.7558	2	21.85	Y
K.IKGEHPGLSIGD.V	Y	49.85	1221.6353	0.9	611.8254	2	32.11	N
K.KLGEMWNNTAADDKQPY.E	Y	49.4	1979.9047	0.9	990.9604	2	49.19	N
K.HPDASVNFSEF.S	Y	49.37	1248.541	-0.6	625.2774	2	56.72	N
K.FEDM(+15.99)AK.A	Y	48.03	755.316	-0.9	378.6649	2	12.43	Y
K.LGEMWNNTAADD.K	Y	47.53	1335.5402	1.3	668.7782	2	49.14	N
K.TMSAK(+42.01)EK.G	Y	47.12	835.4109	-2.4	418.7117	2	13.02	Y
K.KHPDASVNFSEF.S	Y	47.03	1376.636	1.5	689.3263	2	51.06	N
M.WNNTAADDKQPYEK.K	Y	45.95	1678.7587	1.5	840.3878	2	25	N
K.IKGEHPGLSIG.D	Y	45.76	1106.6084	-0.3	554.3113	2	31.78	N
L.SIGDVAK.K	Y	44.93	688.3755	0.2	345.1951	2	17.05	N
K.KLGEMWNNTAAD.D	Y	44.7	1348.6082	-0.7	675.3109	2	44.25	N
K.GKFEDM(+15.99)AK.A	Y	44.46	940.4324	1.3	471.2241	2	12.88	Y
K.DPNAPK.R	Y	44.31	640.318	0.2	321.1663	2	9.58	N
K.KFK(+42.01)DPNAPK.R	Y	43.88	1085.5869	-0.8	362.8693	3	18.81	Y
K.KLGEMWNN.T	Y	42.51	990.4593	0.2	496.237	2	40.88	N
K.M(+15.99)SSYAFFVQ.T	Y	42.32	1094.4742	0.2	548.2445	2	63.69	Y
K.GEHPGLSIGD.V	Y	41.87	980.4563	-1	491.2349	2	33.71	N
K.RPPSAFFL.F	Y	41.87	933.5072	1.5	467.7616	2	64.82	N

K.LGEMWNNTAADDKQPY.E	Y	41.74	1851.8097	2.4	926.9144	2	52.64	N
K.KLGEM(+15.99)WNNTAAD.D	Y	41.33	1364.603	4.6	683.312	2	33.44	Y
K.KHPDASVNFSE.F	Y	41.3	1229.5676	2.5	615.7926	2	28.22	N
K.KLGEMWNNTA.A	Y	40.84	1162.5441	2.1	582.2805	2	43.83	N
K.KLGEMWN.N	Y	40.39	876.4164	1	439.2159	2	42.29	N
K.FKDPN.A	Y	39.9	619.2966	-2.3	310.6548	2	13.1	N
K.KLGEM(+15.99)WNNTAADDKQPY.E	Y	39.87	1995.8997	0.8	666.3077	3	40.48	Y
K.KHPDASVNFS.E	Y	39.48	1100.525	-0.1	551.2697	2	25	N
K.KGVVK(+42.01)AEK.S	Y	38.84	899.5439	-0.1	450.7792	2	12.16	Y
K.GKPDAAK.K	Y	38.22	685.3759	2	343.6959	2	4.93	N
I.KGEHPGLSIGDVAK.K	Y	38.2	1406.7517	0.3	704.3834	2	36.28	N
K.GK(+42.01)PDAAK.K	Y	37.68	727.3864	7.8	364.7033	2	11.95	Y
K.GVVK(+42.01)AEK.S	Y	36.13	771.449	1.3	386.7323	2	14.33	Y
D.DKQPYEK.K	Y	35.5	906.4446	-0.1	454.2296	2	11.2	N
E.HPGLSIGDVAK.K	Y	35.49	1092.5928	0.1	547.3037	2	36.86	N
K.RPPSAFFLF.C	Y	35.29	1080.5756	-1	541.2945	2	73.37	N
N.FSEFSK.K	Y	35.15	743.349	2.1	372.6826	2	26	N
K.KHPDASVN.F	Y	34.45	866.4246	0.6	434.2198	2	10.15	N
K.LGEM(+15.99)WNNTAADDK(+42.01)QPYEK.K	Y	33.5	2166.9529	4.1	723.3279	3	33.39	Y
K.RPPSAFF.L	Y	33.31	820.4231	2.1	411.2197	2	49.81	N
K.IK(+42.01)GEHPGLSIGDVAK.K	Y	33.27	1561.8463	-2.3	781.9286	2	44.21	Y
K.GKPDAAKK.G	Y	33.01	813.4708	0.2	407.7428	2	5.36	N
K.KHPDASVNF.S	Y	32.96	1013.493	3.5	507.7556	2	28.59	N
A.DDKQPYEK.K	Y	32.48	1021.4716	-0.5	511.7428	2	12.28	N
G.KFEDMAK.A	Y	31.28	867.416	0.2	434.7154	2	21.18	N
K.LGEMWNNTAADDKQPYE.K	Y	30.94	1980.8523	3.2	991.4366	2	51.5	N
K.KC(+57.02)SER.W	Y	30.29	678.3119	-1.8	340.1626	2	3.72	Y
K.HPDASVNFSEFS.K	Y	30.26	1335.5731	-0.5	668.7935	2	53.5	N
K.QPYEK.K	Y	29.94	663.3228	-1.5	332.6682	2	10.02	N
K.KLGEM(+15.99)WNNTAADD.K	Y	29.82	1479.63	-1.8	740.8209	2	34.13	Y
K.LGEM(+15.99)WNNTAAD.D	Y	29.63	1236.5081	2.3	619.2627	2	38.07	Y
K.KHPDASVNFSEFSK(+42.01)K.C	Y	29.12	1761.8685	-0.6	441.4741	4	43.32	Y
K.LKEKYEK.D	Y	29.07	936.528	-0.7	469.2709	2	11.48	N
K.EKYEK.D	Y	28.81	695.349	-0.4	348.6816	2	7.42	N
R.EEHKK.K	Y	28.78	669.3445	-2.1	335.6788	2	1.88	N
K.HPDASVNFS.E	Y	28.14	972.4301	-2.2	487.2212	2	29.92	N
F.FVQTC(+57.02)REEHK.K	Y	27.39	1332.6244	1.8	667.3207	2	12.53	Y
K.K(+42.01)LGEM(+15.99)WNNTAADDKQPYEK.K	Y	27.02	2295.0476	0.6	766.0236	3	32.15	Y
P.RGKM(+15.99)SSYAFFVQTC(+57.02)R.E	Y	26.8	1852.8712	-18.2	927.426	2	60.88	Y
Y.EKDIAAYR.A	Y	24.95	964.4977	1.5	483.2568	2	27.32	N
K.MSSYAFFVQT.C	Y	24.54	1179.527	2	590.7719	2	67.58	N
L.GEMWNNTAADDKQPYEK.K	Y	24.3	1995.8633	9.2	666.3011	3	37.15	N
K.PDAAKK.G	Y	24.12	628.3544	-0.8	315.1842	2	4.06	N

F.KDPNAPK.R	Y	23.93	768.413	0.6	385.214	2	15.5	N
K.QPYEKK.A	Y	23.68	791.4177	-0.8	396.7158	2	8.78	N
K.ARYER.E	Y	23.59	693.3558	-0.2	347.6851	2	9.52	N
M.GK(+42.01)GDPK.K	Y	23.53	642.3337	-0.4	322.174	2	9.18	Y
K.MSSYAFFVQ.T	Y	23.5	1078.4794	0.7	540.2473	2	67.33	N
K.IKGEHPGL.S	Y	22.61	849.4708	1.7	425.7434	2	19.42	N
K.LGEM(+15.99)WNNTAADDKQPYEKK.A	Y	22.53	2253.0371	-0.8	752.019	3	30.71	Y
W.NNTAADDKQPYEK.K	Y	21.75	1492.6793	4	747.3499	2	14.08	N
K.HPDASVNFSEFSK(+42.01)K.C	Y	20.51	1633.7736	0.7	817.8947	2	49.62	Y
R.EMKTYIPPK.G	Y	19.12	1105.5841	-14.7	553.7912	2	28.66	N
K.KLGEM(+15.99)WNNTAADDK(+42.01)QPYEK.K	Y	18.49	2295.0476	3.8	574.7714	4	31.05	Y
K.TYIPPKGETK(+42.01)K.K	Y	18.13	1302.7183	5	652.3696	2	20.76	Y
Y.IPPKGETK.K	Y	17.8	868.5018	1.1	435.2586	2	19.01	N
K.KKHPDASVNFSEF.S	Y	15.42	1504.731	1.4	753.3738	2	45.36	N
K.K(+42.01)HPDASVNFSEFSKK.C	Y	14.56	1761.8685	-16.6	881.9269	2	57.97	Y
K.HPDASVNFS(+79.97)EFSK.K	Y	14.17	1543.6344	4.6	772.828	2	57.89	Y
K.MSSYAFFVQTC(+57.02)REEH.K	Y	14.04	1890.8029	-0.1	631.2748	3	56.4	Y
K.EKGKFEDM(+15.99)AK.A	Y	14.04	1197.5699	1.5	599.7932	2	14.22	Y
R.AK(+42.01)GKPDAAKK.G	Y	13.85	1054.6134	1.5	528.3148	2	9.92	Y
R.EM(+15.99)K(+42.01)TYIPPK.G	Y	13.43	1163.5896	5.8	582.8054	2	32.53	Y
K.K(+42.01)LGEM(+15.99)WNNTAADDK(+42.01)QPYEK.K	Y	12.91	2337.0583	0.4	585.2721	4	33.26	Y
P.K(+42.01)RPPS(+79.97)AFFLFC(+57.02)SEYRPK.I	Y	12.9	2251.0649	-3.5	751.3596	3	31.9	Y
K.LKEK(+42.01)YEK.D	Y	12.9	978.5386	-3.7	490.2747	2	16.11	Y
K.KC(+57.02)SERWK.T	Y	12.28	992.4861	-3.8	497.2484	2	43.49	Y
K.RPPS(+79.97)AFFLFC(+57.02)SEYRPK.I	Y	11.86	2080.9592	5.5	1041.4927	2	67.87	Y
E.KDIAAYR.A	Y	10.77	835.4551	0.4	418.735	2	26.97	N
D.PKKPR.G	Y	10.21	624.4071	-0.2	313.2108	2	4.11	N
Y.EREMK(+42.01)TYIPPK.G	Y	10.1	1432.7384	-13.5	717.3668	2	32.71	Y
R.WK(+42.01)T(+79.97)MSAK.E	Y	7.94	972.414	-3.3	487.2127	2	20.78	Y
K.FEDMAKADKAR.Y	Y	7.69	1280.6183	-4.6	641.3135	2	50.83	N
E.KY(+79.97)EKDIAAYR.A	Y	6.5	1335.6223	0.3	668.8186	2	37.3	Y
W.NNTAADDKQPYEKK.A	Y	6.5	1620.7743	18.2	811.4092	2	11.71	N
R.EMK(+42.01)TYIPPKGETK.K	Y	5.78	1562.8014	-0.2	782.4078	2	31.52	Y
M.SAKEK(+42.01)GK.F	Y	5.72	788.4392	1.6	395.2275	2	17.05	Y
K.TYIPPKGET(+79.97)K.K	Y	5.69	1212.5791	-6.4	607.2929	2	41.88	Y
K.YEKDIAAYRA.K	Y	5.49	1198.5981	2.5	600.3079	2	28.27	N

2.9 Raw LC-MS/MS data for PTM profiling of HMGB1 subform D

Peptide	Unique	-10lgP	Mass	ppm	m/z	Z	RT	PTM
K.RPPSAFFLFC(+57.02)SEYRPK.I	Y	142.9	2000.993	2.8	501.2569	4	68.16	Y
K.RPPSAFFLFC(+57.02)SEYR.P	Y	125.47	1775.845	3.7	592.9579	3	68.99	Y

K.LGEMWNNTAADDKQPYEK.K	Y	112.33	2108.947	-1.9	1055.479	2	43.47	N
K.KHPDASVNFSEFSK.K	Y	110.47	1591.763	1.5	398.9486	4	41.22	N
K.MSSYAFFVQTC(+57.02)R.E	Y	110.4	1495.659	0.2	748.8369	2	68.78	Y
K.M(+15.99)SSYAFFVQTC(+57.02)REEHK.K	Y	109.57	2034.893	0.5	679.3052	3	45.88	Y
K.HPDASVNFSEFSK.K	Y	109.09	1463.668	2.4	732.8431	2	75.4	N
P.PSAFFLFC(+57.02)SEYRPK.I	Y	107.54	1747.839	2.4	583.6217	3	68.25	Y
K.M(+15.99)SSYAFFVQTC(+57.02)REEHKK.K	Y	107.52	2162.988	-1.4	541.7535	4	37.91	Y
K.LGEMWNNTAADDKQPYEKK.A	Y	103.95	2237.042	-0.7	1119.528	2	37.49	N
K.M(+15.99)SSYAFFVQTC(+57.02)R.E	Y	103.62	1511.654	0.1	756.8342	2	65.71	Y
K.KLGEM(+15.99)WNNTAADDKQPYEK.K	Y	103.34	2253.037	7.8	564.2709	4	41.08	Y
K.LGEM(+15.99)WNNTAADDKQPYEK.K	Y	103.02	2124.942	0	1063.478	2	41.57	Y
R.GK(+42.01)M(+15.99)SSYAFFVQTC(+57.02)R.E	Y	101.9	1738.781	-0.5	870.3972	2	57.85	Y
R.PPSAFFLFC(+57.02)SEYRPK.I	Y	99.94	1844.892	-0.3	615.971	3	69.24	Y
R.GKMSSYAFFVQTC(+57.02)R.E	Y	99.41	1680.775	2.2	841.3967	2	55.39	Y
K.KLGEMWNNTAADDKQPYEK.K	Y	99.16	2237.042	-3.5	746.6854	3	43.38	N
K.KHPDASVNFSEFSKK.C	Y	98.42	1719.858	-0.1	430.9717	4	31.26	N
R.GKM(+15.99)SSYAFFVQTC(+57.02)R.E	Y	94.26	1696.77	-0.1	849.3923	2	50.08	Y
K.IKGEHPGLSIGDVAK.K	Y	93.55	1519.836	-0.7	760.9246	2	29.29	N
K.GEHPGLSIGDVAKK.L	Y	93.42	1406.752	1.3	352.6956	4	30.52	N
S.AFFLFC(+57.02)SEYRPK.I	Y	92.64	1563.754	-1.2	782.8835	2	64.82	Y
R.GK(+42.01)MSSYAFFVQTC(+57.02)R.E	Y	92.51	1722.786	-9.2	862.3922	2	64.62	Y
K.TYIPPK.G	Y	91.97	717.4061	0.7	359.7106	2	20.62	N
P.SAFFLFC(+57.02)SEYRPK.I	Y	91.15	1650.786	-1.8	826.3989	2	64.92	Y
R.GKMSSYAFFVQTC(+57.02)REEHK.K	Y	90.57	2204.014	0.9	552.0113	4	47.38	Y
G.EMWNNTAADDKQPYEK.K	Y	90.27	1938.842	-0.6	647.2875	3	35.27	N
K.FKDPNAPKRPPSAFFLFC(+57.02)SEYRPK.I	Y	88.54	2898.464	1.6	580.7009	5	62.41	Y
K.TYIPPKGETK.K	Y	87.74	1132.613	1.7	567.3146	2	19.3	N
N.NTAADDKQPYEK.K	Y	85.44	1378.637	3.3	690.3278	2	12.45	N
G.KMSSYAFFVQTC(+57.02)R.E	Y	85.23	1623.754	2.5	542.2599	3	54.96	Y
L.GEMWNNTAADDKQPYEK.K	Y	83.83	1995.863	2.4	998.9413	2	35.72	N
K.LGEMWNNTAADDK.Q	Y	82.64	1463.635	2.2	732.8264	2	41.3	N
H.PDASVNFSEFSKK.C	Y	81.12	1454.704	-0.7	485.9083	3	43.95	N
K.MSSYAFFVQTC(+57.02)REEHK.K	Y	80.92	2018.898	4.5	505.734	4	51.59	Y
G.EHPGLSIGDVAK.K	Y	80.71	1221.635	0.7	408.2193	3	37.13	N
K.MSSYAFFVQTC(+57.02)REEHKK.K	Y	80.3	2146.993	2.5	1074.506	2	46.86	Y
A.FFLFC(+57.02)SEYRPK.I	Y	79.89	1492.717	1.4	498.5804	3	64.61	Y
K.IKGEHPGLSIGDVAKK.L	Y	79.15	1647.931	-0.1	550.3174	3	29.44	N
R.EMKTYIPPKGETK.K	Y	76.91	1520.791	-0.6	761.4022	2	20.91	N
K.HPDASVNFSEFSK(+42.01).K	Y	76.67	1505.679	4.5	753.85	2	52.71	Y
P.DASVNFSEFSK.K	Y	76.49	1229.556	5.4	615.7888	2	52.18	N
S.YAFFVQTC(+57.02)R.E	Y	75.61	1190.554	3	596.2861	2	54.22	Y
K.KKHPDASVNFSEFSK.K	Y	75.61	1719.858	0.4	574.2935	3	33.36	N
E.MWNNTAADDKQPYEK.K	Y	75.53	1809.799	0.1	905.907	2	29.81	N

W.NNTAADDKQPYEK.K	Y	75.48	1492.679	2.3	747.3486	2	12.49	N
K.KLGEM(+15.99)WNNTAADDK.Q	Y	75.13	1607.725	3.1	536.9172	3	27.99	Y
H.PDASVNFSEFSK.K	Y	74.74	1326.609	3.1	664.3139	2	51.31	N
K.YEKDIAAYR.A	Y	74.6	1127.561	1.7	564.7888	2	25.9	N
N.TAADDKQPYEKK.A	Y	74.41	1392.689	15.2	697.3621	2	10.54	N
K.LGEM(+15.99)WNNTAADDK.Q	Y	73.42	1479.63	0.3	740.8225	2	36.29	Y
P.DASVNFSEFSKK.C	Y	72.46	1357.651	-3.1	679.8309	2	43.45	N
P.KIKGEHPGLSIGDVAK.K	Y	72.2	1647.931	1.8	412.9907	4	30.53	N
K.IK(+42.01)GEHPGLSIGDVAK.K	Y	71.5	1561.846	-3.7	781.9275	2	42.9	Y
Y.AFFVQTC(+57.02)REEHK.K	Y	71.08	1550.73	-0.7	776.3717	2	29.14	Y
K.RPPSAFFLFC(+57.02).S	Y	69.94	1240.606	0.3	621.3105	2	72.18	Y
K.FKDPNAPK.R	Y	69.69	915.4814	0.7	306.1679	3	11.24	N
M.WNNTAADDKQPYEK.K	Y	69.69	1678.759	1.7	840.388	2	23.67	N
K.GEHPGLSIGDVAK.K	Y	69.06	1278.657	0.9	640.3362	2	37.63	N
Y.AFFVQTC(+57.02)R.E	Y	68.8	1027.491	1.1	514.7533	2	40.27	Y
N.NTAADDKQPYEKK.A	Y	67.57	1506.731	2.5	503.2523	3	10.96	N
F.C(+57.02)SEYRPK.I	Y	67.52	938.428	1.4	470.2219	2	10.87	Y
K.IKGEHPGLS(+79.97)IGDVAK.K	Y	67.23	1599.802	-6.7	800.903	2	35.96	Y
K.RPPSAFFLFC(+57.02)SE.Y	Y	66.53	1456.681	4.4	729.3509	2	70.98	Y
W.NNTAADDKQPYEKK.A	Y	66.45	1620.774	1.9	811.3959	2	11.11	N
K.RPPSAFFLFC(+57.02)SEYRPKIKG.E	Y	65.23	2299.194	2.4	767.407	3	64.26	Y
F.FLFC(+57.02)SEYRPK.I	Y	65.14	1345.649	1.2	449.5574	3	59.2	Y
D.ASVNFSEFSKK.C	Y	64.75	1242.624	0	415.2154	3	37.87	N
G.KM(+15.99)SSYAFFVQTC(+57.02)R.E	Y	64.66	1639.749	2.5	547.5915	3	50.07	Y
F.LFC(+57.02)SEYRPK.I	Y	64.3	1198.58	1.6	600.2985	2	29.7	Y
E.HPGLSIGDVAK.K	Y	63.62	1092.593	-2	547.3026	2	33.5	N
K.RPPSAFFLFC(+57.02)SEY.R	Y	63.58	1619.744	1.1	810.8802	2	72.52	Y
K.DIAAYR.A	Y	62.94	707.3602	1.7	354.688	2	22.24	N
L.GEM(+15.99)WNNTAADDKQPYEK.K	Y	62.72	2011.858	2.9	1006.939	2	28.82	Y
R.AK(+42.01)GKPDAAK.K	Y	62.55	926.5185	1.7	309.8473	3	9.22	Y
R.EM(+15.99)KTYIPPKGETK.K	Y	62.08	1536.786	-1.7	769.3988	2	15.46	Y
K.FEDMAK.A	Y	60.75	739.321	2.1	370.6686	2	17.88	N
A.FFVQTC(+57.02)R.E	Y	60.67	956.4538	1.6	479.2349	2	33.24	Y
K.GEHPGLSIGDVAKKL.G	Y	60.63	1519.836	0	380.9662	4	47.16	N
E.M(+15.99)WNNTAADDKQPYEK.K	Y	60.36	1825.794	0.8	913.905	2	26.54	Y
S.SYAFFVQTC(+57.02)R.E	Y	60.35	1277.586	1.2	639.8012	2	60.46	Y
K.C(+57.02)SERWK.T	Y	59.75	864.3912	2.3	433.2039	2	12.49	Y
K.GKFEDMAKADK.A	Y	59.19	1238.596	14.1	413.8786	3	37.38	N
K.DPNAPK.R	Y	58.97	640.318	2.3	321.167	2	9.15	N
K.EKGKFEDMAK.A	Y	58.94	1181.575	-0.2	394.8655	3	14.1	N
K.TYIPPK(+42.01)GETK.K	Y	58.6	1174.623	1.6	588.3198	2	27.8	Y
K.KLGEMWNNT(+79.97)AADDKQPYEK.K	Y	58.33	2317.009	-3.7	773.3406	3	41.09	Y
H.PGLSIGDVAK.K	Y	57.84	955.5338	1	478.7747	2	41.81	N

K.EKGKFEDM(+15.99)AK.A	Y	57.81	1197.57	2.2	599.7936	2	12.46	Y
K.K(+42.01)FKDPNAPK.R	Y	57.69	1085.587	1.9	543.8018	2	17.06	Y
K.GKFEDMAK.A	Y	57.56	924.4375	1.7	309.1536	3	15.8	N
K.KLGEMWNNTAADDKQPY.E	Y	57.35	1979.905	0.9	660.9761	3	48.52	N
K.K(+42.01)HPDASVNFSEFSK.K	Y	57.33	1633.774	-0.7	545.5981	3	44.07	Y
F.FVQTC(+57.02)R.E	Y	57.01	809.3854	0.6	405.7002	2	13.37	Y
K.M(+15.99)SSYAFFVQTC(+57.02)REEH.K	Y	56.91	1906.798	5	636.6097	3	52.04	Y
K.FEDMAKADKAR.Y	Y	56.43	1280.618	5	641.3196	2	17	N
K.KFKDPNAPK.R	Y	56.37	1043.576	-0.8	522.795	2	13.96	N
D.ASVNFSEFSK.K	Y	55.72	1114.529	1.3	558.2727	2	47.66	N
K.IKGEHPGLSIGD.V	Y	55.72	1221.635	0.6	611.8253	2	30.14	N
K.TYIPPKGETKK.K	Y	54.39	1260.708	1.4	421.2438	3	12.05	N
G.LSIGDVAK.K	Y	54.02	801.4596	0.8	401.7374	2	36.48	N
K.KHPDASVNFSEF.S	Y	53.87	1376.636	1.1	689.326	2	50.62	N
A.SVNFSEFSK.K	Y	53.17	1043.492	1.6	522.7543	2	44.91	N
K.GEHPGLSIGD.V	Y	52.9	980.4563	1.2	491.236	2	31	N
K.KHPDAS(+79.97)VNFSEFSK.K	Y	52.85	1671.729	-3	836.8694	2	41.86	Y
K.EKYEKDIAAYR.A	Y	52.51	1384.699	14.9	693.3669	2	27.28	N
K.MSSYAFFVQTC(+57.02)REEH.K	Y	52.43	1890.803	2.6	631.2766	3	56.28	Y
I.KGEHPGLSIGDVAK.K	Y	51.79	1406.752	0.1	469.9246	3	32.78	N
K.LGEM(+15.99)WNNTAADDKQPY.E	Y	50.72	1867.805	0.2	934.9098	2	48.1	Y
K.KKHPDASVNFSEF.S	Y	50.6	1504.731	0.2	502.5844	3	43.19	N
K.DIAAYRAKGKPDAAK.K	Y	50.22	1573.858	2.7	525.6279	3	16.87	N
K.RPPSAFFL.F	Y	49.68	933.5072	1.3	467.7615	2	64.5	N
S.VNFSEFSK.K	Y	49.01	956.4603	1.4	479.2381	2	42.57	N
R.WKTM(+15.99)SAK.E	Y	48.93	866.432	0.4	434.2234	2	10.73	Y
K.HPDASVNFSEF.S	Y	48.16	1248.541	0.6	625.2781	2	56.17	N
K.GKPDAAKKGVVK.A	Y	48.05	1196.724	0.7	599.3697	2	9.38	N
L.FC(+57.02)SEYRPK.I	Y	48	1085.496	1.9	362.8401	3	19.94	Y
K.GKFEDM(+15.99)AKADK.A	Y	47.58	1254.591	5	628.3061	2	12.43	Y
K.HPDAS(+79.97)VNFSEFSK.K	Y	47.53	1543.634	6.2	772.8293	2	49.16	Y
A.DDKQPYEK.K	Y	47.41	1021.472	0.4	511.7433	2	10.96	N
K.K(+42.01)LGEM(+15.99)WNNTAADDKQPYEK.K	Y	46.68	2295.048	2.5	574.7706	4	28.18	Y
K.LGEM(+15.99)WNNTAADD.K	Y	46.47	1351.535	1.1	676.7755	2	37.59	Y
K.GK(+42.01)PDAAK.K	Y	45.96	727.3864	1.7	364.7011	2	10.96	Y
K.M(+15.99)SSYAFFVQ.T	Y	45.71	1094.474	1.4	548.2452	2	63.65	Y
F.FVQTC(+57.02)REEHKK.K	Y	44.95	1460.719	0.3	731.3671	2	9.84	Y
K.KLGEMWNN.T	Y	44.63	990.4593	0.1	496.237	2	38.79	N
K.ARYER.E	Y	44.29	693.3558	9	347.6883	2	8.76	N
R.AK(+42.01)GKPDAAKK.G	Y	44.24	1054.613	3.5	352.5463	3	8.85	Y
K.KHPDASVNFSEFSK(+42.01).K	Y	43.45	1633.774	1	817.8949	2	42.91	Y
K.KLGEMWNNTAAD.D	Y	43.44	1348.608	-0.6	675.3109	2	43.25	N
K.GKPDAAK.K	Y	43.28	685.3759	1.1	343.6956	2	5.52	N

Y.AFFVQTC(+57.02)REEHKK.K	Y	43.23	1678.825	2.3	840.4216	2	24.37	Y
T.AADDKQPYEK.K	Y	43.19	1163.546	-6.3	582.7765	2	11.44	N
K.RPPSAF.F	Y	42.52	673.3547	0.4	337.6848	2	20.98	N
K.KHPDASVN.F	Y	42.26	866.4246	2.2	434.2206	2	9.16	N
K.KC(+57.02)SERWK.T	Y	42	992.4861	0.4	497.2505	2	10.53	Y
K.RPPSAFFLF.C	Y	41.95	1080.576	0.7	541.2954	2	73.34	N
C.SEYRPK.I	Y	41.8	778.3973	0.8	390.2062	2	9.6	N
K.YEKDIAAY.R	Y	41.63	971.46	1.1	486.7378	2	29.74	N
M.KTYIPPK.G	Y	41.19	845.501	0.6	423.7581	2	14.04	N
K.KLGEMWNNT.A	Y	41.15	1091.507	1.9	546.7618	2	41.2	N
K.FEDM(+15.99)AK.A	Y	40.94	755.316	0.6	378.6655	2	11.94	Y
K.FKDPN.A	Y	40.8	619.2966	-0.5	310.6554	2	11.92	N
Y.EKDIAAYR.A	Y	40.48	964.4977	1	483.2566	2	17.8	N
K.RPPSAFF.L	Y	39.79	820.4231	1.8	411.2195	2	48.13	N
K.GKFEDM(+15.99)AK.A	Y	39.6	940.4324	0.2	471.2235	2	16.55	Y
K.MSSYAFFVQT.C	Y	37.83	1179.527	2.3	590.7721	2	67.46	N
K.KLGEMWN.N	Y	37.4	876.4164	1.4	439.2161	2	40.22	N
K.KLGEM(+15.99)WNNTA.A	Y	37.09	1178.539	-2.3	590.2755	2	30.8	Y
K.IKGEHPGL.S	Y	36.79	849.4708	1.4	425.7433	2	17.15	N
K.HPDASVNFS.E	Y	36.79	972.4301	-0.4	487.2221	2	28.4	N
K.LGEM(+15.99)WNNTAADDKQPYEKK.A	Y	36.07	2253.037	2.4	752.0214	3	33.32	Y
L.SIGDVAK.K	Y	35.46	688.3755	0.6	345.1953	2	15.55	N
K.IKGEHPGLSIGDVA.K	Y	35.43	1391.741	1.1	696.8784	2	42.92	N
K.KHPDASVNFSE.F	Y	35.1	1229.568	0.3	615.7913	2	26.27	N
K.IKGEHPGLSIG.D	Y	34.45	1106.608	-0.2	554.3113	2	29.41	N
N.FSEFSK.K	Y	34.33	743.349	3.2	372.683	2	24.75	N
K.LGEMWNNTAADDKQPY.E	Y	34.18	1851.81	4	926.9158	2	52.55	N
K.LGEMWNNTAADD.K	Y	33.94	1335.54	2	668.7787	2	49.01	N
K.KGVVK(+42.01)AEK.S	Y	33.24	899.5439	0.7	450.7796	2	11	Y
G.EHPGLSIGDVAKK.L	Y	33.11	1349.73	2.3	450.9184	3	30.68	N
K.LKEKY(+79.97)EK.D	Y	33.09	1016.494	2.3	509.2556	2	28.85	Y
D.DKQPYEK.K	Y	32.79	906.4446	0.3	454.2297	2	10.17	N
K.M(+15.99)SSYAFF.V	Y	32.7	867.3473	1.6	434.6816	2	64.39	Y
K.KKHPDASVNF.S	Y	32.37	1141.588	1	571.8018	2	20.72	N
K.HPDASVNFSEFS.K	Y	31.41	1335.573	2.4	668.7954	2	53.25	N
K.KLGEM(+15.99)WNNTAADDKQPY.E	Y	30.78	1995.9	-1	998.9561	2	39.08	Y
K.IKGEHPGLSIGDVAK(+42.01).K	Y	30.65	1561.846	0.8	521.6231	3	36.36	Y
K.LGEM(+15.99)WNNTAAD.D	Y	30.34	1236.508	1.7	619.2623	2	37.21	Y
K.MSSYAFFVQ.T	Y	29.83	1078.479	-1	540.2464	2	67.28	N
K.GVVKAEK.S	Y	29.5	729.4385	1.6	365.7271	2	9.08	N
K.QPYEK.K	Y	29.35	663.3228	1.6	332.6692	2	9.43	N
Y.IPPKGETK.K	Y	29.27	868.5018	0.1	435.2582	2	22.2	N
K.RPPSAFFLFC(+57.02)S.E	Y	28.83	1327.638	-0.2	664.8263	2	71.27	Y

K.M(+15.99)SSYAFFVQT.C	Y	28.03	1195.522	0.7	598.7687	2	64.44	Y
K.TYIPPKG.E	Y	28.03	774.4276	0.6	388.2213	2	17.52	N
R.EEHKK.K	Y	27.61	669.3445	0	335.6795	2	3.63	N
K.EKYEK.D	Y	27.53	695.349	1.1	348.6821	2	5.67	N
F.KDPNAPK.R	Y	26.63	768.413	-0.2	385.2137	2	13.36	N
K.M(+15.99)SSYAF.F	Y	26.36	720.2789	-1.2	721.2853	1	43.44	Y
K.QPYEKK.A	Y	26.26	791.4177	0.8	396.7165	2	7.19	N
K.KHPDASVNFS.E	Y	26.2	1100.525	1.3	551.2705	2	23.67	N
K.LKEKYEK.D	Y	25.86	936.528	0.3	469.2714	2	9.88	N
K.KLGEMWNNTAADDKQ.P	Y	24.96	1719.789	-1.1	574.2695	3	36.16	N
R.WKTMSAK.E	Y	24.39	850.4371	2.9	851.4469	1	33.01	N
G.KPDAAK.K	Y	23.94	628.3544	-0.2	315.1844	2	3.7	N
L.SIGDVAKK.L	Y	23.71	816.4705	2.7	409.2436	2	30.65	N
R.EM(+15.99)K(+42.01)TYIPPK.G	Y	23.31	1163.59	6.4	582.8058	2	16.67	Y
E.KDIAAYR.A	Y	20.97	835.4551	1.9	418.7356	2	15.84	N
K.K(+42.01)HPDASVNFSEFSKK.C	Y	20.83	1761.869	1.1	881.9425	2	41.95	Y
V.QTC(+57.02)REEHK.K	Y	20.36	1086.488	1.7	544.252	2	11.2	Y
K.LGEM(+15.99)WNNTAADDK(+42.01)QPYEK.K	Y	20.32	2166.953	6.2	542.7488	4	23.29	Y
G.KFEDM(+15.99)AK.A	Y	19.63	883.4109	-0.8	442.7124	2	11.28	Y
K.FKDPNA.P	Y	19.54	690.3337	1.2	346.1745	2	14.15	N
S.VNFSEFSKK.C	Y	18.82	1084.555	-0.4	543.2847	2	31.05	N
K.FEDMAKADKA.R	Y	18.81	1124.517	2.9	563.2675	2	17.75	N
K.KLGEM(+15.99)WNNTAADDK(+42.01)QPYEK.K	Y	18.35	2295.048	5.3	574.7722	4	20.83	Y
K.KLGEMWNNTA.A	Y	18.07	1162.544	-0.7	582.2789	2	42.63	N
K.T(+79.97)YIPPKGETK.K	Y	17.79	1212.579	16	405.2068	3	17.31	Y
K.DPNAPKRPPSAFFLFC(+57.02)SEYRPK.I	Y	17.5	2623.301	3.3	875.4437	3	64.13	Y
K.QPYEKKAAK.L	Y	17.04	1061.587	6.3	531.8041	2	12.67	N
A.ADDK(+42.01)QPYEK.K	Y	16.94	1134.519	-2.1	568.2657	2	34.68	Y
D.KQPYEK.K	Y	15.94	791.4177	1.7	396.7168	2	9.39	N
M.GK(+42.01)GDPK.K	Y	15.9	642.3337	5.1	322.1758	2	8.82	Y
S.EYRPK.I	Y	15.08	691.3653	-14	346.6851	2	25.2	N
V.NFSEFSKK.C	Y	14.81	985.4869	1.5	493.7515	2	30.98	N
K.GKPDAAKK.G	Y	14.72	813.4708	11.6	407.7474	2	1.01	N
K.YEKDIAAYRA.K	Y	14.41	1198.598	1.7	600.3074	2	26.65	N
R.WKTM(+15.99)SAKEK.G	Y	14.06	1123.57	0.3	562.7922	2	10.06	Y
K.KKHPDASVN.F	Y	13.35	994.5195	5	498.2695	2	8.56	N
K.KHPDASVNFSEFS(+79.97)K(+42.01)K.C	Y	12.6	1841.835	-16.4	921.9095	2	42.05	Y
I.KGEHPGLSIGDVAK(+42.01)K.L	Y	12.02	1576.857	1.4	526.6271	3	36.05	Y
G.EM(+15.99)WNNTAADDKQPYEK.K	Y	11.47	1954.837	1.4	978.4269	2	28.34	Y
A.SVNFSEFSKK.C	Y	11.34	1171.587	-1.8	586.7999	2	43.28	N
P.PS(+79.97)AFFLFC(+57.02)SEYRPK(+42.01)IK.G	Y	11.17	2110.995	17.4	1056.523	2	75.77	Y
P.PKGETK.K	Y	10.56	658.365	0	330.1898	2	15.14	N
Y.IPPKGETKK.K	Y	9.8	996.5967	1.7	499.3065	2	12.09	N

T.AADDKQPYEKK.A	Y	9.34	1291.641	-0.6	646.8273	2	30.53	N
D.PKKPR.G	Y	9.25	624.4071	0.2	313.2109	2	3.27	N
N.FSEFSKK(+42.01)C(+57.02)SER.W	Y	8.22	1445.661	2.1	723.8392	2	52.84	Y
K.KLGEM(+15.99)WNNTAADDKQ.P	Y	8.19	1735.784	0.7	868.8997	2	27.83	Y
K.KHPDASVNFSEFS(+79.97)K.K	Y	8.08	1671.729	-2.2	836.8701	2	49.04	Y
R.WK(+42.01)TMSAKEK.G	Y	8.03	1149.585	8.1	575.8045	2	27.49	Y
R.EMKTY(+79.97)IPPK.G	Y	7.43	1185.55	-19.5	396.183	3	14.32	Y
K.GKPDAAK(+42.01)KG.V	Y	7.15	912.5028	14.9	457.2655	2	9.16	Y
M.KT(+79.97)YIPPKGETK.K	Y	6.79	1340.674	0.8	671.3448	2	42.82	Y
K.HPDASVNFSEFSK(+42.01)K.C	Y	6.32	1633.774	12.2	817.9041	2	52.58	Y
S.VNFSEFSKK(+42.01)C(+57.02)SER.W	Y	6.23	1658.772	0.6	830.3939	2	54.04	Y
K.DPNAP.K	Y	5.93	512.223	3.3	513.232	1	16.14	N
K.KFK(+42.01)DPNAPK.R	Y	5.68	1085.587	10.8	543.8066	2	56.3	Y
R.GKM(+15.99)SSYAFF.V	Y	5.39	1052.464	3.7	527.2411	2	55.71	Y
P.KIK(+42.01)GEHPGLSIGDVAK.K	Y	5.18	1689.941	-4.8	845.9739	2	61.81	Y

2.10 Acetylated HMGB1 interacts with p53 and its phosphorylated forms

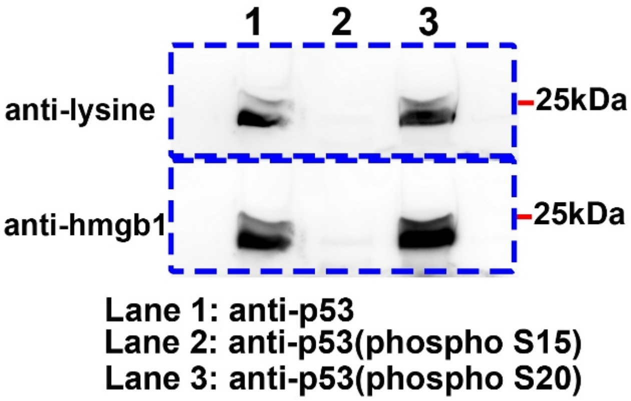

Figure S9. Co-immunoprecipitation assay of HMGB1 protein with antibody of p53 and its phosphorylated forms. Proteins from Co-IP are first immunobloted with anti-lysine antibody. Then the membrane is stripped with strip buffer (1.5% SDS, 20 mM glysin, pH 2.0). The stripped membrane is immunoblotted with anti-HMGB1 under the same condition. Three antibodies were used in this assay. P53 antibody recognizes total cellular p53 proteins. Anti-p53 (phospho S15) and anti-p53 (phospho S20) only immunoreact with p53 proteins which are specifically phosphorylated on serine 15 and serine 20.
